# Advanced PROTAC and Quantitative Proteomics Strategy Reveals Bax Inhibitor-1 as a Critical Target of Icaritin in Burkitt Lymphoma

**DOI:** 10.3390/ijms252312944

**Published:** 2024-12-02

**Authors:** Peixi Zhang, Ziqing Zhang, Jie Li, Meng Xu, Weiming Lu, Ming Chen, Jiaqi Shi, Qiaolai Wang, Hengyuan Zhang, Shi Huang, Chenlei Lian, Jia Liu, Junjie Ma, Jieqing Liu

**Affiliations:** School of Medicine, Huaqiao University, Quanzhou 362021, China; 21013071029@stu.hqu.edu.cn (P.Z.);

**Keywords:** icaritin, PROTACs, quantitative proteomics, target identification, mechanism of action, anti-tumor

## Abstract

Understanding the molecular targets of natural products is crucial for elucidating their mechanisms of action, mitigating toxicity, and uncovering potential therapeutic pathways. Icaritin (ICT), a bioactive flavonoid, demonstrates significant anti-tumor activity but lacks defined molecular targets. This study employs an advanced strategy integrating proteolysis targeting chimera (PROTAC) technology with quantitative proteomics to identify ICT’s key targets. A library of 22 ICT-based PROTAC derivatives were synthesized, among which **LJ-41** exhibited a superior IC50 of 5.52 μM against Burkitt lymphoma (CA-46) cells. Then, differential proteomic analysis identified Bax inhibitor-1 (BI-1) as a potential target. Target validation techniques, including cellular thermal shift assay (CETSA), drug affinity responsive target stability (DARTS) assay, surface plasmon resonance (SPR) assay, and molecular docking, confirmed LJ-41’s high specificity for BI-1. Mechanistic investigations revealed that **LJ-41** induces apoptosis through BI-1 degradation, triggering endoplasmic reticulum stress and activating inositol-requiring enzyme 1 α (IRE1α), activating transcription factor 6 (ATF6), and nuclear factor erythroid 2-related factor transcription factor heme oxygenase 1 (NRF2-HO-1) signaling pathways. This study establishes a refined methodological framework for natural product target discovery and highlights ICT-PROTAC derivatives’ potential for clinical application in Burkitt lymphoma treatment.

## 1. Introduction

Natural products exhibit a diverse range of pharmacological and biological activities, encompassing anti-tumor, anti-inflammatory, anti-oxidant, anti-fibrotic, and more [1]. As a consequence, research on lead compounds derived from natural products and the development of novel therapeutic agents has garnered significant attention [2]. Clarifying the specific mechanisms underlying the actions of active natural products not only facilitates a profound understanding of drug mechanisms, the identification of potential toxicities, and the mitigation of possible resistance mechanisms but also holds the potential to unveil novel disease-specific targets, representing a pivotal stride in the advancement of innovative drug development [3]. In recent years, researchers have been fervently exploring innovative target discovery methods, leading to a proliferation of diverse approaches. Among these, one of the most intriguing and cutting-edge techniques involves the integration of emerging proteolysis targeting chimera technology with differential proteomics to unveil the targets of natural products. This approach has been ingeniously applied in the discovery and validation of anti-inflammatory targets for lathyrane diterpenoids [4]. Moreover, in a recent study, researchers successfully employed this method to identify the target of artemisinin [5].

While this method of identifying natural product targets has been robustly demonstrated, PROTAC technology excels in amplifying the distinctions in target protein alterations through protein degradation and analyzing protein differences via differential proteomics, enabling the discovery of potential targets. Furthermore, it has gained widespread acceptance and implementation among numerous researchers [5]. However, certain limitations persist within this method. For instance, during its initial proposal, researchers typically established only the PROTAC and control groups, a practice that has endured in subsequent research applications, warranting our scrutiny. There is still room for improvement in terms of the grouping design of this method. To address this, we have refined the method by introducing a natural product group, enhancing the criteria for screening potential target proteins, and selecting the natural product ICT as our experimental subject to elucidate its role in the anti-Burkitt lymphoma effect. These enhancements have notably advanced the methodology.

Icaritin, the primary bioactive compound found in Epimedium, is widely employed in the treatment of cardiovascular diseases [6,7], osteoporosis [8,9], and it exhibits neuroprotective effects [10] by mitigating inflammation and oxidative damage associated with neurological diseases [11]. Furthermore, ICT has demonstrated significant inhibitory effects on the proliferation of various malignant tumor cells, including hematological malignancies such as leukemia [12], myeloma [13], liver cancer [14,15], breast cancer [16,17], prostate cancer [18], and neurologically related cancers [19]. Recent years have witnessed substantial advancements in our understanding of ICT’s mechanisms of action against tumors. Studies have unveiled its ability to induce tumor cell apoptosis through the Janus kinase 2 (JAK2)/signal transducer and activator of transcription 3 (STAT3)/cyclin-dependent kinase inhibitory protein-1 (p21) pathway [12] and the JNK1 signaling pathway [20]. Notably, ICT, marketed as “Icaritin Soft Capsules”, received approval from the National Medical Product Administration (NMPA) on 12 January 2022, for the treatment of hepatocellular carcinoma (HCC) [21]. However, the precise anti-tumor targets of ICT remain undisclosed, posing potential risks for the subsequent use of ICT capsules. Given the considerable anti-tumor potential of ICT, we believe that identifying its specific targets is imperative to comprehensively grasp its molecular mechanisms in anti-tumor therapy and ensure the drug’s safety.

BI-1 exhibits endoribonuclease inhibitor activity and binds to ubiquitin-protein ligases. It plays a crucial role in multiple biological processes, including the negative regulation of RNA metabolic processes, the negative regulation of the intrinsic apoptotic signaling pathway, and the response to L-glutamate. BI-1 functions upstream of, or within, the negative regulation of calcium ion transport into the cytosol. This protein is located in the endoplasmic reticulum membrane and mitochondrial membrane, and it serves as a biomarker for cervical squamous cell carcinoma and prostate carcinoma. While downregulation of BI-1 has been shown to induce apoptosis in human nasopharyngeal carcinoma cell lines [22,23] and reduce cell viability in prostate cancer cells [24], no small-molecule drugs specifically targeting BI-1 have been identified thus far.

The mechanism related to BI-1 is mostly directed to the endoplasmic reticulum stress (ERS) pathway. The endoplasmic reticulum (ER) is an important membrane organelle with a unique structure, and the functions of the ER include protein synthesis, folding, transport, calcium homeostasis, and lipid biosynthesis, and the disruption of the ER homeostasis due to exogenous stimuli, such as drugs and ultraviolet rays, will lead to the accumulation of misfolded and unfolded proteins, which triggers ERS and triggers the unfolded protein response (UPR) [25]. ERS affects cell survival or death. The activation of the UPR transmits stress signals from the ER to the nucleus, leading to the regulation of relevant genes. Ultimately, this process restores endoplasmic reticulum homeostasis, and promotes cell survival by reducing protein synthesis, promoting proper protein folding, and accelerating the degradation of misfolded and unfolded proteins. Whereas the continued application of pharmacological stimuli during ERS will lead to cell death, ERS has been shown to be an additional regulatory pathway for apoptosis [26]. ERS mediates apoptosis through IRE1α, protein kinase R-like endoplasmic reticulum kinase (PERK), ATF6, and Ca^2+^. When ERS is activated, IRE1α/tumor necrosis factor receptor-associated factor 2 (TRAF2) and IRE1α/X-Box binding protein 1(XBP1)/C/EBP homologous protein (CHOP) signaling pathways mediate apoptosis; PERK induces apoptosis by activating downstream CHOP through the activating transcription factor 4 (ATF4) signaling pathway, and ATF6 activates its downstream CHOP to activate apoptosis. In addition, caspase9-dependent induction of apoptosis by regulating the Ca^2+^-induced translocation pathway from the ER to the mitochondria.

In this study, we have refined the method for target discovery through the combination of PROTAC technology and differential proteomics. This optimization enabled us to identify the anti-tumor targets of ICT. The enhancement of the method involved an expansion in the number of experimental groups. We evaluated the anti-tumor efficacy of a range of synthetic ICT-PROTACs using CA-46 cells and utilized differential proteomics to identify proteins exhibiting distinctions between the PROTAC-treated and natural product-treated groups, rendering them potential targets. To verify the precision of these potential targets, we employed a combination of assays, including DARTS assay, CETSA, competitive assay, and SPR assay, to elucidate the specific target of ICT in the context of human Burkitt lymphoma and to elucidate their subsequent pathways of action based on target degradation.

## 2. Results

### 2.1. Design and Synthesis of ICT-PROTACs

A series of 22 ICT-based PROTAC compounds were synthesized (Scheme S1–S8), featuring a combination of ICT derivatives as the protein of interest (POI) ligands, linkers of varying lengths and compositions, and E3 ligands such as lenalidomide or Von Hippel–Lindau (VHL) ligands (Figure 1, Table 1) (Figures S1–S26).

### 2.2. ICT-PROTACs Inhibited the Tumor Cells’ Proliferation

Among the synthesized PROTACs, Compound **10** (**LJ-41**) exhibited excellent anti-tumor activity in CA-46 cells, with an IC_50_ of 5.52 μM, surpassing ICT’s IC_50_ by more than tenfold. Compound 8 also demonstrated strong activity but was excluded due to limited solubility. These findings established **LJ-41** as the lead compound for further evaluation (Figure 2, Table 2).

### 2.3. Proteomic Profiling Identifies BI-1 as a Potential Target of **LJ-41**

To investigate potential targets of ICT against human Burkitt Lymphoma cells (CA-46 cells), we utilized ICT in combination with lenalidomide to form a tertiary complex known as **LJ-41**, featuring a 3-PEG linker (Figure 3A). A tandem mass tagging (TMT)-based quantitative proteomics approach was employed to delve deeper into the targets impacted by **LJ-41** degradation and the changes in protein folding following a 35 μM **LJ-41** treatment of CA-46 cells for 48 h (Figure 3). In this experiment, a total of 6518 quantitative proteins were identified (see Figure S27). The heatmap of differential proteins revealed that the expression of these proteins was influenced by treatments with **1a** and PROTAC, displaying differential expression in each group (Figure 3G). By considering the functionality of these proteins, we identified those associated with tumorigenesis and development that exhibited significant differences in protein levels, as depicted in a volcano diagram (Figure 3, Table 3 and Table 4).

Kyoto Encyclopedia of Genes and Genomes (KEGG) pathway enrichment analysis revealed that the proteins with notable alterations in their expression levels were predominantly associated with pathways related to glutathione metabolism, the P53 signaling pathway, protein processing in the ER, and more (Figure 3). These findings can serve as a valuable reference for subsequent mechanistic studies, which is further supported by Protein–Protein Interaction Network Analysis (see Figure S28).

### 2.4. **LJ-41** Induces BI-1 Degradation Via the Ubiquitin-Proteasome System

Western blot analysis validated the degradation of BI-1 by **LJ-41** in a dose- and time-dependent manner (Figure 4A,B) (Figure S29). To further investigate whether **LJ-41** induces BI-1 degradation through the ubiquitin-proteasome system, we employed the proteasome inhibitor MG132 to impede the degradation effect. Western blotting analysis revealed that the degradation of BI-1 was effectively blocked in a dose-dependent manner after pre-treatment with MG132. Furthermore, to elucidate the competitive binding dynamics, both **1a** and Lenalidomide were administered concomitantly to contest the interaction of **LJ-41** with BI-1 and the E3 ubiquitin ligase. Both **1a** and Lenalidomide hindered the degradation of BI-1 mediated by **LJ-41** (Figure 4). These findings strongly support the conclusion that the degradation of BI-1 facilitated by **LJ-41** is reliant on the ubiquitin-proteasome system and confirm BI-1 as a novel target of ICT-based PROTAC.

### 2.5. Direct Binding of **LJ-41** to BI-1

To further establish BI-1 as a direct target of **1a** (Figure 4), we utilized SPR assay, CETS) and DARTS assay to assess the binding affinity of **1a** and **LJ-41** with BI-1. In the SPR assay, we examined the direct binding affinity between **LJ-41** and BI-1 protein. As depicted in Figure 5A, **LJ-41** exhibited a robust binding affinity to BI-1 with an equilibrium dissociation constant (KD) value of 7.970 × 10^−5^ M. In the CETSA, **1a** enhanced cellular thermal stability, increasing the tolerance temperature of BI-1 protein up to 67 °C (Figure 5B). In the DARTS assay, the degradation of BI-1 protein in cell lysates induced by pronase was substantially diminished after treatment with **1a** (Figure 5C). These results unequivocally confirm BI-1 as a direct target of **1a** and **LJ-41**.

### 2.6. Molecular Docking Analysis

Subsequently, our investigation encompassed the execution of molecular docking analyses employing the AutoDock Vina software. The docking outcomes underscored the stability of the binding dynamics. Specifically, the 7-OH modification of ICT engendered no discernible alteration in its binding site with BI-1. Remarkably, both ICT and **1a** exhibited an exceptional affinity towards the target proteins, evidenced by their notably favorable binding energies of −7.1 kcal/mol and −7.0 kcal/mol, respectively (Figure 6A,B).

Concomitantly, molecular docking procedures unveiled an intricate network of interactions. Notably, ICT formed hydrophobic engagements with four key amino acids within BI-1 protein: PHE118, PHE218, LEU99, and MET38 (Figure 6C). Additionally, a distinct Pi–Pi stacking interaction transpired with PHE39. Impressively, ICT PROTAC **LJ-41** showcased a parallel pattern of molecular interaction, forging hydrophobic alliances with eight amino acid residues—MET38, PHE39, ALA42, TYR46, LEU99, LEU103, LEU114, and PHE118 (Figure 6D)—within the protein structure. Noteworthy, **LJ-41** outperformed its precursor, demonstrating a superior binding force with a compelling binding energy of −7.8 kcal/mol. The findings elucidated above offer an intuitive clarity: both ICT and ICT PROTACs LJ41 hold the inherent potential to foster more extensive interactions with BI-1 proteins.

### 2.7. **LJ-41**-Induced Apoptosis Via ERS Pathways

The functional consequence of BI-1 degradation was explored by assessing apoptosis markers. Western blot analysis revealed that **LJ-41** treatment led to the activation of several apoptosis-related proteins. Specifically, the activation of IRE1α and its downstream signaling through TRAF2 and Caspase-12 was observed, along with increased levels of phosphorylated ATF6 and CHOP, indicating the induction of apoptosis Via the ER stress response (Figure 7). Additionally, the NRF2-HO-1 pathway was activated, suggesting that oxidative stress contributed to apoptosis. These findings confirm that the degradation of BI-1 by **LJ-41** induces apoptosis in CA-46 cells through multiple ER stress-related pathways.

## 3. Discussion

In this study, we refined the integration of PROTAC and differential proteomics to identify BI-1 as the anti-tumor target of ICT. The target was validated through DARTS, CETSA, SPR, and molecular docking to confirm its precision. Our findings indicate that the optimized method of PROTAC combined with differential proteomics enhances the accuracy of target protein discovery, offering a novel approach to target identification in natural products. This elucidation of the ICT target lays a solid foundation for future research endeavors.

### 3.1. Enhanced PROTACs for Improved Anti-Tumor Efficacy

**Figure 1:**
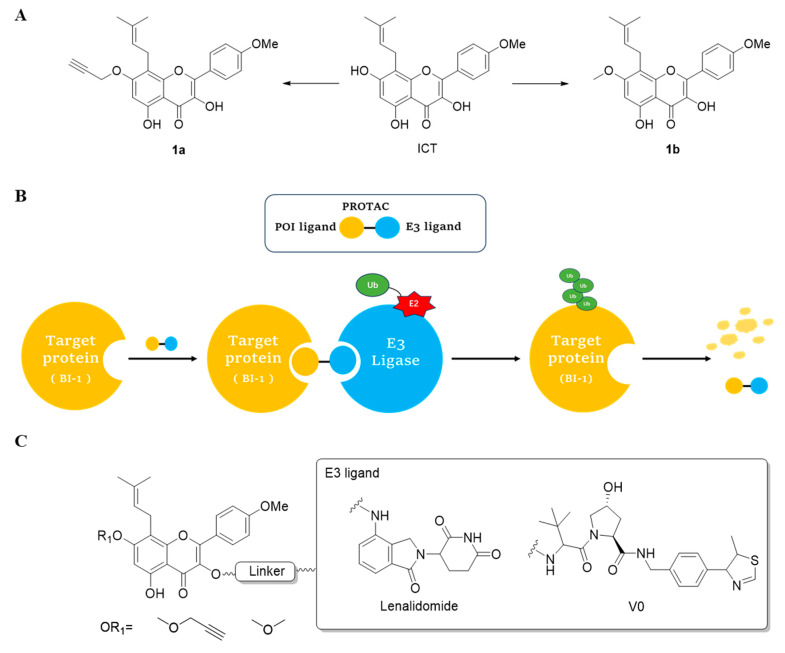
The design of ICT PROTACs. (**A**) Chemical structure diagram of ICT and POI ligands designed on its basis. (**B**) Diagram of the mechanism of PROTACs-induced degradation of target proteins. (**C**) The chemical structure of each component of ICT-PROTACs.

**Figure 2:**
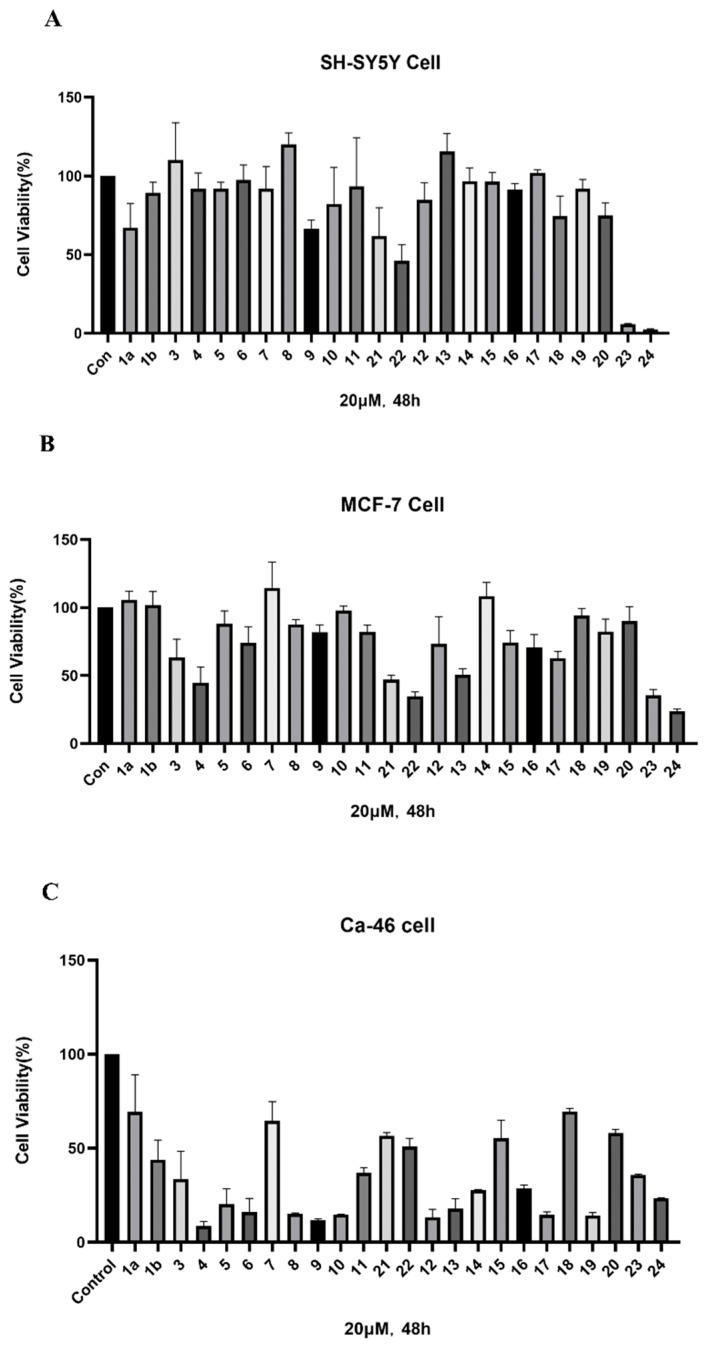
Cell viability (%) after compound action. (**A**) The effect of compounds on the viability of SH-SY5Y cells. (**B**) Effects of compounds on the activity of MCF-7 cells. (**C**) The effect of compounds on the viability of CA-46 cells. Data are presented as mean ± SEM.

**Figure 3:**
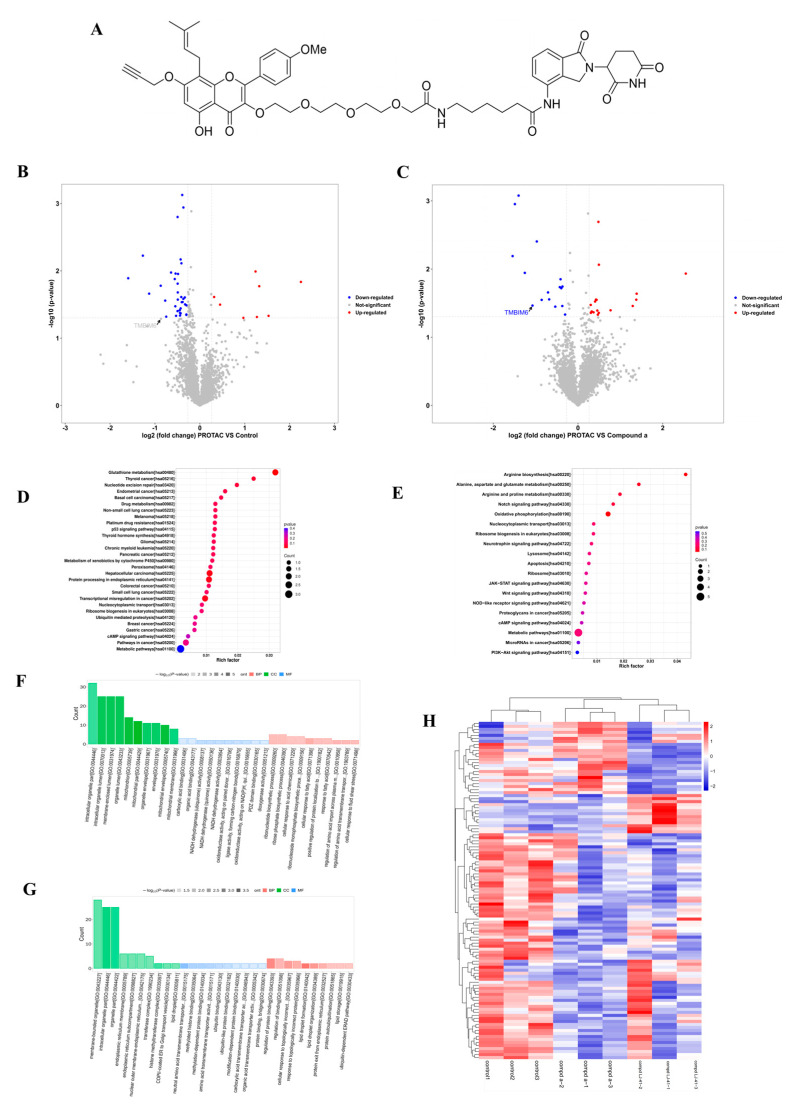
TMT differential proteomics reveals that BI-1 is a potential target of anti-CA-46 cell action for **LJ-41**. (**A**) Structure of compound **LJ-41**. (**B**) The downregulated (blue) and upregulated (red) proteins associated with the tumor were plotted as fold change (**LJ-41**/Control) versus −Log10 of the *p*-value (*t*-test). (**C**) The downregulated(blue) and upregulated (red) proteins associated with the tumor were plotted as fold change (**LJ-41**/**1a**) versus −Log10 of the *p*-value (*t*-test). (**D**) The fold change in Kyoto Encyclopedia of Genes and Genomes (KEGG) pathway enrichment after 48 h treatment of **LJ-41** or DMSO control. (**E**) The fold change in Kyoto Encyclopedia of Genes and Genomes (KEGG) pathway enrichment after 48 h treatment of **LJ-41** or **1a**. (**F**) Classification histogram for GO enrichment analysis of differentially expressed proteins after 48 h treatment of **LJ-41** or DMSO control. (**G**) Classification histogram for GO enrichment analysis of differentially expressed proteins after 48 h treatment of **LJ-41** or **1a**. (**H**) Differentially expressed proteins were analyzed by hierarchical clustering and visualized by heat maps to visualize the changing patterns of differences found between experimental groups of differentially expressed proteins.

**Figure 4:**
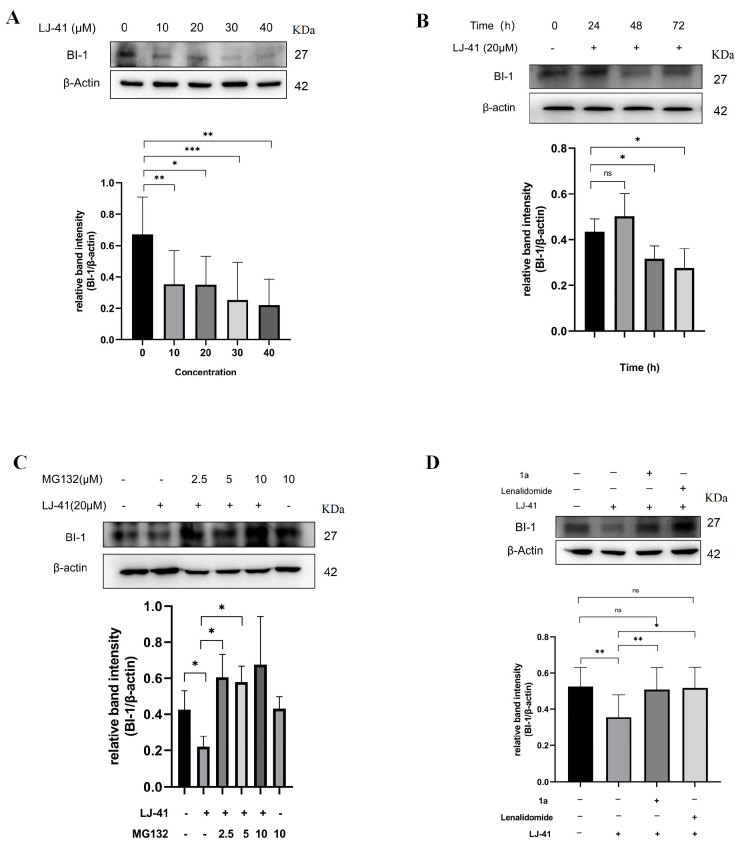
BI-1 is a degradation target of **LJ-41**. (**A**) Immunoblot analysis of concentration-dependent degradation of BI-1 protein in CA-46 cells. (**B**) Immunoblot analysis of time-dependent degradation of BI-1 protein in CA-46 cells. (**C**) Immunoblot analysis of BI-1 protein in CA-46 cells pre-treated with MG132 for 12 h and subsequently treated with either DMSO or **LJ-41** for 48 h. (**D**) Immunoblot analysis of BI-1 protein in CA-46 cells treated by **LJ-41** together with either 1a or Lenalidomide for 48 h. Immunoblot analysis data were normalized with β-Actin. Data are presented as mean ± SEM. ^ns^ *p* > 0.05, * *p* < 0.05, ** *p* < 0.005, *** *p* < 0.001 compared with the 0 μM control group.

**Figure 5:**
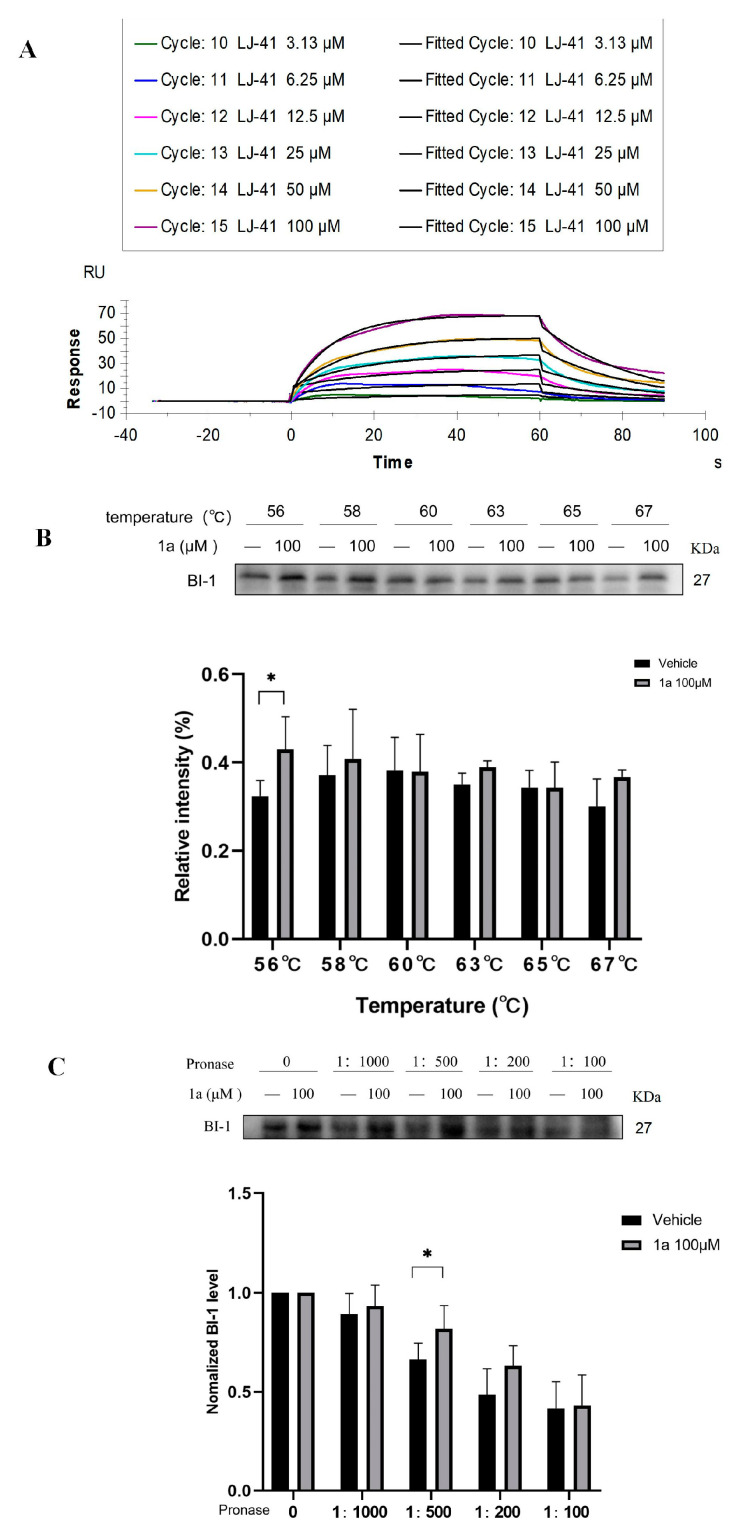
**1a** directly binds to BI-1. (**A**) Fitted plot of affinity determination of **LJ-41** with BI-1. (**B**) CETSA to detect the thermal stability of cellular BI-1 protein. (**C**) DARTS assay to test the stability of cellular BI-1 protein under enzyme degradation. Data are presented as mean ± SEM. * *p* < 0.05 compared with the control group.

**Figure 6:**
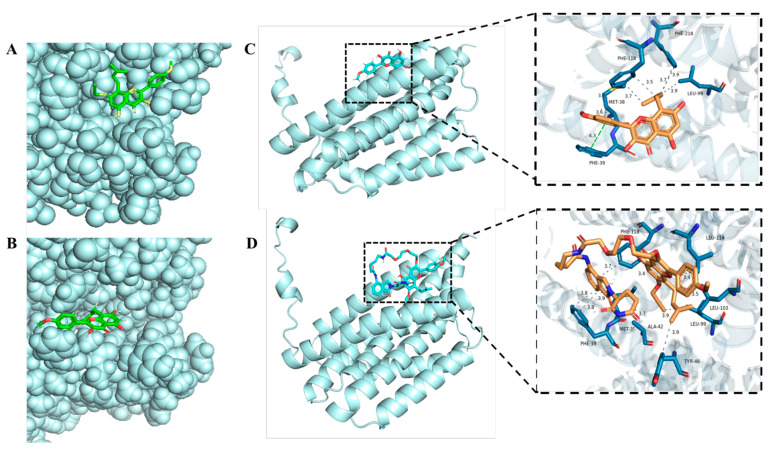
Molecular docking simulation. (**A**) Binding pattern of compound a with the target protein BI-1 (UniProtKB/Swiss-Prot: P55061), compound a is a green rod-like structure. (**B**) Diagram of the binding pattern of ICT to the target protein BI-1, ICT is a blue rod-like structure. (**C**) Schematic simulation of molecular docking between ICT and BI-1, and ICT is represented as an orange rod-like structure, the green solid line indicates Pi–Pi stacking, and the gray dashed line represents hydrophobic interactions. (**D**) Schematic diagram of molecular docking simulation of ICT-PROTAC (**LJ-41**) with BI-1, and ICT-PROTAC (**LJ-41**) is represented as an orange rod-like structure, the green solid line indicates Pi–Pi stacking, and the gray dashed line represents hydrophobic interactions.

PROTACs comprise a triadic ensemble, embracing a protein of interest ligand, a linker, and an E3 ligand. Our approach entailed the individualized design of PROTACs for each of these intricate components. The inaugural stage involved the meticulous conception and subsequent synthesis of POI ligands. Elucidative insights gleaned from the investigation by Fangqing Zhang and Zhenwei Wu et al. [27]. underscored the divergent impacts of 3-OH and 7-OH modifications upon ICT’s bioactivity. Specifically, derivatives bearing a 7-OH substitution exhibited a marked attenuation in their inhibitory effects against MCF-7, MDA-MB-231, and A549 cells, whereas the converse held true for 3-OH-substituted ICT derivatives wherein the activity witnessed a notable augmentation. Notably, the introduction of a larger moiety at the 3-OH terminus did not precipitate a concomitant decline in activity, further substantiating its strategic utility. Importantly, the concurrent presence of 7-OH and 3-OH was found to be inconducive to the rational design and synthesis of PROTAC compounds. Consequently, we embarked on a structural metamorphosis centered on the 7-position of ICT, culminating in the ingenious design and successful synthesis of ICT derivatives (Figure 1A).

Subsequently, our focus shifted to the meticulous design and systematic refinement of the Linker component. Drawing upon the current landscape of the literature pertinent to PROTAC technology, Linkers exhibit a tripartite structural classification: lipophilic aliphatic alkane chains, hydrophilic polyethylene glycol chains (often embracing hydrophilic appendages, e.g., piperazines), and rigid linker moieties characterized by the integration of inherently inflexible structures, including benzene rings, imidazoles, triazoles, among others [28]. Furthermore, acknowledging the pivotal role that linker chain length assumes in shaping the efficacy of PROTAC molecules, we embarked on the adoption of aliphatic alkane chains within this framework [29]. This selection was primarily aimed at delineating an optimal linker length (or atom count) in an initial pursuit to enhance the relative efficiency of the resultant constructs. After determining the approximate length, given ICT’s inherent water insolubility, our strategic focus extended to augmenting the overall molecule’s aqueous solubility. To achieve this, we embarked on the integration of hydrophilic polyethylene glycol (PEG) chains as Linkers. Distinctly, we selected diethylene glycol and trimethylene glycol as foundational materials to synthesize PEG (n = 2) and PEG (n = 3) Linkers. Both types of Linkers, encompassing alkane chains and PEG-alkane chains, underwent systematic synthesis (Table 1).

Regarding the E3 ligand aspect, lenalidomide, a derivative akin to thalidomide with enhanced water solubility and reduced molecular weight was judiciously chosen as the initial E3 ligand based on the pertinent literature [30]. Subsequent to the creation of PROTACs featuring alkane chain and PEG-integrated alkane chain architectures, a comprehensive survey of pertinent research underscored the potential merits of incorporating a VHL-type ligand to elevate the antitumor activity within PROTAC designs [27]. In light of this insight, a third iteration of ICT PROTACs was synthesized, where the E3 ligand lenalidomide was seamlessly substituted with the VHL-type ligand V0 (Figure 1B). This strategic adaptation sought to capitalize on the advantageous properties associated with VHL-type ligands, thus enhancing the anti-tumor activity of PROTAC (Figure 1C).

### 3.2. Methodological Advancements in Target Identification

Target identification is a pivotal endeavor in the development of novel drugs and the elucidation of pharmacological effects, especially in the context of natural products with unclear mechanisms of action. Consequently, researchers have increasingly dedicated their efforts to enhance the precision and convenience of drug target discovery. Notably, Yanli Wu, Yueying Yang et al. [4]. were pioneers in this field, employing PROTAC technology combined with differential proteomics. They successfully identified the confirmed target, MAFF, for the treatment of inflammation in lathyrane diterpenoids isolated from the seeds of *Euphorbia lathyris* by comparing the downregulated proteins in the PROTAC group vs. the control group [4]. Similarly, Yan Li et al. also employed a similar approach to discover the anti-tumor target, PCLAF, in Artemisinin by analyzing the downregulated proteins in the PROTAC group vs. the control group [5].

Unlike affinity-based target identification, PROTAC molecules do not necessitate deepen embeddings within the highly active regions, such as surface binding pockets, grooves, and slots, of the target protein. Instead, they establish only transient adhesion with the protein’s surface. This implies that the degradation of the target protein can be achieved through specific intermolecular forces, hydrogen bonding, and other weak binding interactions with low binding energies [31]. In essence, the degradation process relies on the formation of target-PROTAC-E3 ligase triple complexes [32,33]. The combination of PROTAC and differential proteomics serves to amplify differences stemming from target proteins. It involves degrading the action target proteins of natural products (aNPs) through PROTACs and subsequently analyzing these distinctions through differential proteomics to uncover potential targets.

In previous studies conducted by researchers using the techniques described above, the experimental design was limited to PROTAC-treated and control groups. These studies validated potential targets by analyzing differential proteins and subsequently confirming them [4]. It is crucial to acknowledge that their research primarily revolved around the difference between proteins in the PROTAC-treated and control groups, and this approach has certain limitations, as illustrated below.

Firstly, it is important to consider that if a small molecule has the capability to independently degrade the target protein, it can impact the abundance of interacting or downstream proteins, resulting in multiple differential protein abundances [34]. Consequently, the differential proteins identified through the comparison between the PROTAC-treated and control groups may not necessarily be the precise target proteins but rather the downstream proteins influenced by the target proteins. Secondly, it is widely acknowledged that PROTACs can degrade proteins with weak binding affinities, which can lead to the emergence of multiple differential proteins. This influx of potential target proteins can impose a significant workload on subsequent studies.

Furthermore, it is crucial to recognize that small molecules directly targeting the binding pocket of target proteins or small molecule ligands binding to corresponding protein receptors may inhibit the proteins’ function, potentially causing a compensatory elevation in their expression. In this context, it would be an intriguing discovery to compare small molecules with small molecule-based synthetic PROTAC groups. Thus, considering the aforementioned challenges, we enhanced the experimental design in our study. During the differential proteomics experiment, we incorporated three groups: the control group, the pre-compound group, and the PROTAC group. The determination of target proteins was based on an analysis of differential proteins in the pre-compound group and PROTAC group. By including the pre-compound group, we aimed to ensure that potential target proteins were not overlooked, as analyzing only the control group and PROTAC group could result in missed target proteins.

### 3.3. BI-1 as a Novel Target for Burkitt Lymphoma

Following the differential protein comparison between the pre-compound group and the PROTAC group, a number of proteins were notably downregulated, including BI-1, NLR Family Pyrin Domain Containing 13(NLRP13), CXXC1, TECPR2, GRIA3, and DENND4C. Notably, NLRP13 is primarily localized in the cytoplasm and belongs to the NLRP protein family, comprising a total of 13 proteins. While current research has primarily focused on NLRP3, particularly regarding inflammation-related mechanisms, other family members like NLRP7 and NLRP13 were initially linked to reproductive genes. These proteins are recognized for their involvement in processes related to protein expression during pregnancy, oocyte development, differentiation, and various other functions [35].

A recent study showed that the knockdown of GRIA3 significantly reduced the proliferation and migration of tumor cells and enhanced apoptosis [36]. DENND4C is expressed mainly in the cytoplasm. Diseases associated with DENND4C include Alkuraya–Kucinskas Syndrome and Spastic Paraplegia 39, Autosomal Recessive. Among its related pathways are vesicle-mediated transport and Rab regulation of trafficking. In recent years there have been studies showing that the knockdown of circDENND4C suppressed proliferation, migration, and glycolysis of colorectal cancer cells [37]. BI-1 is located in the ER. BI-1 has been reported to act as an important anti-tumor protein, which is upregulated in lung cancer [38,39], breast cancer [40,41], prostate cancer [24], and nasopharyngeal cancer [22,23], whereas downregulation of BI-1 induces cancer cell death in both prostate cancer and nasopharyngeal cancer [22,23,24]. For what it is worth, the E3 ligand for PROTAC in our study was lenalidomide, and in the study by Luke M Simpson et al. [42], PROTAC with lenalidomide as the E3 ligand was able to degrade ER proteins more efficiently, so based on the cellular location of these candidate target proteins and their correlation with tumor development, we finally selected BI-1 as a potential target. However, this does not exclude the fact that the above proteins may also be the potential targets of ICT against Burkitt Lymphoma cells, only that nowadays the research of the above proteins, such as NLRP7 and so on, concerning the tumor is not yet very comprehensive, but we cannot deny the possibility of it as a potential target. In addition, there are some other proteins with significant changes, such as TMEM18, which has been associated with obesity in both adults and children. It is one of the most conserved human obesity genes [43], the expression of which can be reduced by **LJ-41**, which may suggest that after ICT-PROTACs are tested in vivo, it may target this protein and cause lethargy, anorexia, and other drug-induced adverse effects. This may provide a new idea for the discovery of adverse drug reactions as well as new applications of compounds.

Furthermore, BI-1 has been studied in several pathways related to apoptosis. First, it can directly inhibit the IRE1α signaling pathway and the splicing of downstream XBP1 mRNA by competitively competing with BAX/BAK for their binding sites for IRE1α [44,45]. Secondly, as BI-1 is located on the ER membrane, it is involved in the regulation of [Ca^2+^] in the ER lumen. BI-1 overexpressing cells have lower resting ER Ca^2+^ levels and higher ER Ca^2+^ leakage, whereas when BI-1 is downregulated, it leads to an increase in mitochondrial [46] Ca^2+^ uptake, which causes apoptosis of the cells due to Ca^2+^ overload. Finally, BI-1 may protect against ROS accumulation under ERS conditions by interacting with microsomal monooxygenase (MMO) members NPR and CYP2E1 [47,48,49], and BI-1 may also counteract ROS by promoting anti-oxidant responses through NRF2 and heme oxygenase 1 [50]. The pathway studies based on BI-1 indeed offer valuable insights into potential mechanisms. The degradation of BI-1 by **LJ-41** may disrupt the formation of BI-1 and IP3R protein complexes, resulting in Ca^2+^ overload and ERS that ultimately leads to cell death. Additionally, **LJ-41** could interfere with the interaction between IRE1α and BI-1, triggering ERS and, consequently, cell death. These proposed mechanisms shed light on how **LJ-41** could exert its effects on cells, particularly in the context of anti-tumor activity and therapeutic targeting of hematomas [51,52], and provide a foundation for further research in this area.

### 3.4. Mechanistic Insights into ER Stress-Induced Apoptosis

ERS facilitated apoptosis through IRE1α, PERK, ATF6, and Ca^2+^. When ERS was activated, IRE1α/tumor necrosis factor receptor-associated factor 2 (TRAF2) and IRE1α/XBP1/CHOP signaling pathways mediated apoptosis, PERK induced apoptosis by activating downstream CHOP through the ATF4 signaling pathway, and ATF6 activated its downstream CHOP to activate apoptosis. In addition, Caspase9 induces apoptosis by regulating the Ca^2+^ induced translocation pathway from the ER to the mitochondria. Our previous results have demonstrated that compound **LJ-41** can bind to the intracellular target protein BI-1 and play a degrading role, leading to a decrease in BI-1 content. This decrease may act as a source of stress, causing ERS in cells. In response to ERS, cells produce an unfolded protein response and an endoplasmic reticulum overload response. These responses help reduce protein synthesis and promote the proper folding of proteins. However, prolonged ERS can lead to overstress and activate the corresponding apoptotic pathway. Simultaneously, ERS can also activate the MMO system of the ER, leading to an increase in intracellular reactive oxygen species, which ultimately triggers oxidative stress and the activation of the NRF2-HO-1 pathway to promote apoptosis in cancer cells.

**Figure 7:**
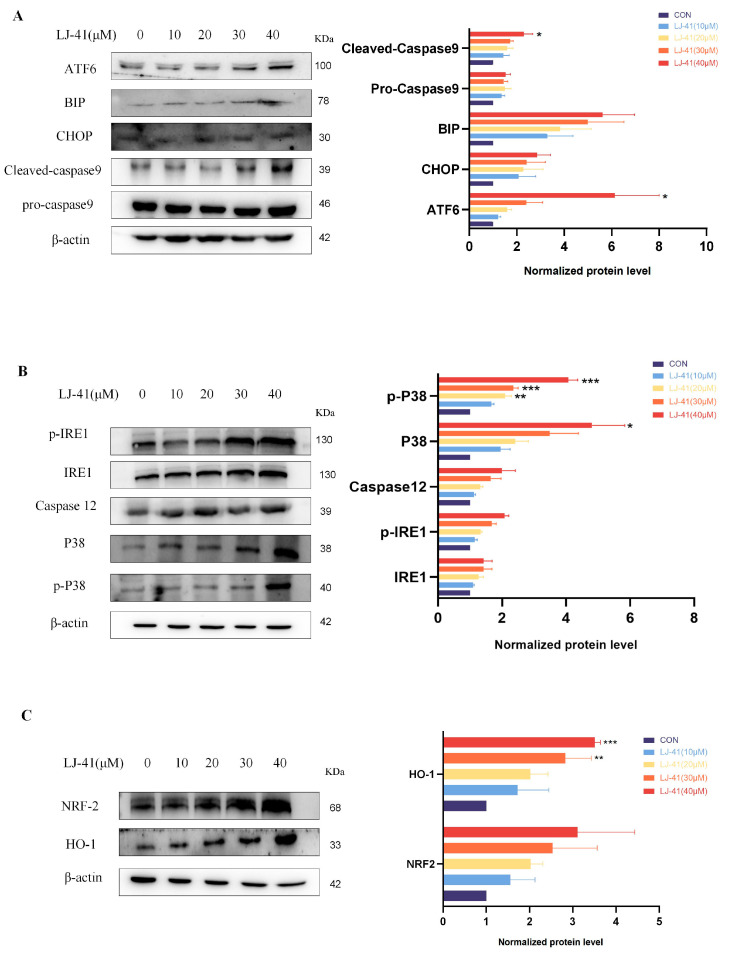
**LJ-41** promotes apoptosis by degrading the BI-1 induced ERS pathway. (**A**) Immunoblot analysis of ATF6, BIP, CHOP, Caspase-9 and Cleaved-caspase 9. (**B**) Immunoblot analysis of P-IRE1, IRE1, Caspase 12 and P38. (**C**) Immunoblot analysis of NRF2 and HO-1. Data are presented as mean ± SEM. * *p* < 0.05, ** *p* < 0.005, *** *p* < 0.001 compared with the control group.

**Figure 8:**
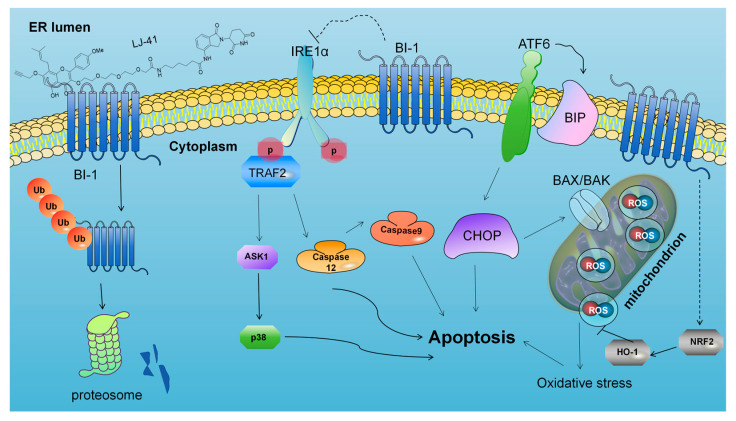
Mechanism of **LJ-41**-induced CA-46 cell death through BI-1 degradation.

Not surprisingly, after the degradation of BI-1 by **LJ-41**, IRE1α first undergoes phosphorylation, forms dimers, and displays increased expression of P-IRE1α with rising drug concentrations. This increase can promote apoptosis through the signaling pathway mediated by TRAF2-catalyzed P38 and Caspase-12. Elevated levels of phosphorylated IRE1α, P38, and Caspase-12 were observed. In addition to the IRE1α-based apoptotic pathway, the ATF6 pathway is also altered, where ATF6, after separation from GRP78/BiP, falls out of the ER membrane and runs to the Golgi apparatus to be cleaved by proteases, generating the transcription factor ATF6p50. This factor enters the nucleus and alleviates endoplasmic reticulum stress at multiple levels by initiating a series of gene transcriptions. However, when ERS become too severe, ATF family proteins also initiate the transcription of CHOP, which initiates a pro-apoptotic program to promote cell apoptosis. The results of the study showed that with increasing concentrations of **LJ-41**, the expression of ATF6 and the content of BiP were upregulated, leading to the activation of Caspase-9 and cell death, as well as an increase in CHOP expression, which promotes apoptosis. Futhermore, the ERS can also activate the MMO system of the ER, leading to an increase in intracellular reactive oxygen species, oxidative stress and activation of the NRF2-HO-1 pathway, which promotes cell apoptosis. Experimental results showed elevated levels of both NRF2 and HO-1 (Figure 7 and Figure 8).

In summary, our study has addressed the need for improved target discovery methods, particularly in the context of identifying and validating targets for the natural product ICT. The enhanced method we have developed overcomes the limitations of previous approaches and ensures that important targets are not missed. The discovery of BI-1 as a target for ICT has significant implications for the development and application of ICT-related drugs, and specifically, the ICT PROTACs, with **LJ-41** as a prominent example, may serve as promising candidates for Burkitt lymphoma treatment. Meanwhile, we investigated the subsequent mechanism of action based on the degradation of BI-1 by **LJ-41**. In specific, the degradation of BI-1 by **LJ-41** activated the IRE1 pathway, the ATF6 pathway, and the NRF2-HO-1 pathway that promoted apoptosis. This work has the potential to advance the field of natural product-based drug discovery and improve the development of treatments for Burkitt lymphoma.

## 4. Materials and Methods

### 4.1. Cell Culture and Chemicals

Human CA-46 cells were obtained from Procell Life Science&Technology Co., Ltd. (Wuhan, China) and grown in Roswell Park Memorial Institute (RPMI) 1640 medium (RPMI-1640, GIBCO, Grand Island, NY, USA) complemented with 20% fetal bovine serum (FBS, SERANA, Berlin, Germany), 100 U/mL penicillin, and 100 μg/mL streptomycin (Solarbio, Beijing, China) and at 37 °C in a humidified atmosphere with 5% CO_2_. For all PROTAC treatment, unless otherwise stated, cells were incubated with chemicals for 48 h. For proteasome inhibition assay, cells were pretreated with MG132 (RHAWN, #R019005, Berlin, Germany) for 2 h followed by PROTAC for 48 h. For the ligand competition experiment, cells were administrated with PROTAC together with Equivalent A or Lenalidomide for 48 h.

### 4.2. CCK-8 Assay

The cells with a density of 8 × 10^4^ cells per mL of CA-46 and 5 × 10^4^ cells per mL of MCF-7 were seeded into a 96-well plate. When cells reached the confluence of 80–90%, 0, 2.5, 5, 10, 20, 40, or 80 µM **1a** (in DMSO), 0, 2.5, 5, 10, 20, 40, or 80 µM **LJ-41** (in DMSO) were added to each well. After 48 h treatment, cell survival and proliferation were determined with the application of CCK-8 (Beyotime, #C0039, Shanghai, China) assay. The OD450 value was quantified under a plate reader.

### 4.3. Mass Spectrometry for Proteomics

Incubate CA-46 cells with **LJ-41**, **1a** or DMSO for 48 h, collect and resuspend in RIPA working solution for 2 min under ice bath ultrasound, fully lyse the sample, centrifuge at 12,000× *g* at 4 °C for 15 min, and quantify protein concentration using BCA assay. Take 100 μg of total protein from each sample and dilute it to 1 mg/mL with H_2_O. Precool with Acetone to −20 °C, add 5 times the volume of acetone to the sample, mix well, and precipitate overnight at −20 °C at 12,000 rpm. Centrifuge at 4 °C for 10 min to remove the supernatant. Add 200 μL of pre-cooled 80% acetone to wash and precipitate twice, centrifuge at 12,000 rpm, and carefully remove the supernatant. Add 100 μL of protein complex solution, dissolve the protein precipitate in a water bath for 3 min, add DTT to 5 mM, oscillate at 55 °C for 20 min, reduce disulfide bonds, cool the sample to room temperature, add IAA to 15 mM, and react in a dark environment for 30 min. Dissolve Trypsin to 0.5 μg/μL in a Resuspension buffer and incubate at room temperature for 5 min. Mix Trypsin with the sample thoroughly in the ratio of Trypsin: protein = 1:50. After simple centrifugation, incubate at 37 °C and 1000 rpm overnight. The sample is centrifuged at high speed and an equal amount of protein is taken into a new EP tube. Label according to the instructions of Thermo’s TMT labeling kit (Waltham, MA, USA) and mix each group of labeled samples in equal amounts. After SDC removal and peptide desalination treatment, the peptide sample was subjected to overnight vacuum drying at 4 °C. After freeze-drying, the peptide sample was redissolved in mobile phase A to 50 ° C, allowing RPUPLC to be separated under alkaline conditions. Chromatographic column: 150 mm × 2.1 mm (Waters, XBridge BEH C18 XP Column, Milford, MA, USA); Mobile phase A: 10 mM ammonium acetate aqueous solution, pH = 10; Mobile phase B: 10 mM ammonium acetate, 10% H_2_O, 90% ACN, pH = 10. Liquid phase gradient for 60 min, mobile phase B: 5% for 2 min, 5–30% for 40 min, 30–40% for 10 min, 40–90% for 4 min, 90% for 2 min, and 2% for 2 min. Collect one component every 1 min, cycle collection, merge into 12 components, vacuum dry, and freeze at −80 °C. After separation by the nano UPLC liquid phase system EASY-nLC1200 (Thermo Fisher, Waltham, MA, USA), data were collected using a mass spectrometer equipped with a nanoliter ion source (Q Active HFX) (Thermo Fisher, USA). Chromatographic separation using 100 μ ID × Using a 15 cm reverse phase chromatographic column (Reprosil Pur 120 C18-AQ, 1.9 μm, Dr. Maisch)(Thermo Fisher, USA). The mobile phase adopts an acetonitrile–water–formic acid system, with mobile phase A consisting of 0.1% formic acid 98% aqueous solution (acetonitrile 2%), and B phase consisting of 0.1% formic acid 80% acetonitrile solution (water 20%). After the chromatographic column is equilibrated with 100% A phase equilibrium, the sample is directly loaded into the chromatographic column by the automatic sampler, and then separated by the chromatographic column gradient. The flow rate is 300 nL/min, and the gradient duration is 90 min. Mobile phase B ratio: 2–5% for 2 min, 5–22% for 68 min, 22–45% for 16 min, 45–95% for 2 min, and 95% for 2 min. Mass spectrometry analysis uses data-dependent acquisition (DDA) mode, with a total analysis time of 90 min, and adopts positive ion detection mode. The obtained data were analyzed through database search using Proteome Discoverer software (PD) (version 2.4.0.305, Thermo Fisher Scientific, USA) and the built-in Sequence HT search engine. Using statistical methods to screen differentially expressed proteins, the screening criteria for differentially expressed proteins are *p*-value < 0.05 and fold Change ≤ 0.83 or fold change ≥ 1.2 for Student’s *t*-test.

### 4.4. Protein Extraction and Western Blotting

When the cell growth reached about 80% of the degree of fusion, the corresponding compounds were added in different treatments. After a 48 h continuous culture, the cells were harvested and lysed in the ice-cold lysis buffer (RIPA). After being separated by SDS-polyacrylamide gel electrophoresis (SDS-PAGE), the protein was transferred onto PVDF membranes and then blocked by the blocking solution with the addition of 5% skim milk powder (bioforxx, German). The membranes were then incubated with the primary antibodies of BI-1 and β-actin, respectively, at 4 °C overnight, and subsequently incubated with the horseradish peroxidase (HRP)-conjugated secondary antibody for 1 h at room temperature. The hybridization band was imaged by a Tanon5200 automatic chemiluminescence analyzer (Tanon Science & Technology Co., Ltd., Shanghai, China), and quantified with the imaging software of ImageJ (ij153-win-java8, Media Cybernetics, Rockville, MD, USA).

### 4.5. Cellular Thermal Shift Assay (CETSA)

The ability of compounds to bind with and stabilize the target BI-1 in vitro was assessed by cellular thermal shift assay. Equivalent CA-46 cell proteins were treated with DMSO or **1a** (100 μM) for 2 h after BCA quantification and then aliquot into six PCR tubes and heated for 5 min to 56, 58, 60, 63, 65, or 67 °C. After centrifuged at 14,000× *g* for 10 min at 4 °C, the supernatant was collected for further immunoblot analysis.

### 4.6. Drug Affinity Responsive Target Stability (DARTS) Assay

The cells were collected and total protein was isolated using RIPA lysis buffer (Beyotime, China). BCA quantification stabilizes protein content at 3 mg/mL and then treated with 100 μM of **1a** or DMSO as control. After incubation for 2 h at room temperature, pronase E (RHAWN, #R052076, China) was added to the lysates for a further 30 min at room temperature. Reactions were ceased by adding the sample buffer and boiling for 15 min and then analyzed via Western blotting analysis.

### 4.7. Surface Plasmon Resonance (SPR) Assay

PBS (10X), pH 7.4 was diluted 10-fold with deionized water to prepare running buffer PBS (2 mM KH_2_PO_4_, 137 mM NaCl, 10 mM Na_2_HPO_4_, 2.7 mM KCl, 5% DMSO, pH 7.4), which was filtered through a 0.22 μM membrane and used. The ligand BI-1 was diluted 20-fold with fixation reagent (10 mM sodium acetate, pH 4.0). First, the surface of the CM5 chip was activated with 400 mM EDC and 100 mM NHS for 480 s at a flow rate of 10 μL/min. Second, the diluted ligand BI-1 was injected into the experimental channel (Fc4) at a flow rate of 10 μL/min, respectively, with a fixation volume of about 2600 RU, and the chip was closed with 1 M ethanolamine at 10 μL/min for 480 s. The chip was then activated with 400 mM EDC and 100 mM NHS at a flow rate of 10 μL/min. The reference channel (Fc3) was only activated and closed without coupling the ligand. The analytes were then subjected to multiple cycle analysis, and the results were analyzed using the Biacore T200 Evaluation Software (version 3.2 - Cytiva), and the kinetic 1:1 binding model was chosen for the fitting.

### 4.8. Molecular Docking Assay

The molecular docking was facilitated using the AutoDock Tools1.5.7, accessible at (http://vina.scripps.edu/ (accessed on 25 November 2023)). Ligands and proteins essential for the docking process were prepared. Concerning the target proteins, their crystal structures were procured from the PDB database (https://www.rcsb.org/ (accessed on 29 November 2023)) which underwent necessary pre-processing steps. This involved the elimination of hydrogenation, modified amino acids, energy optimization, and adjustment of force field parameters. The ligand structure was sourced from the PubChem database (https://pubchem.ncbi.nlm.nih.gov/ (accessed on 29 November 2023)) after attaining a low-energy conformation of the ligand structure.

The ensuing molecular docking entailed the juxtaposition of the target structure with the active ingredient’s structure. This intricate task was achieved using the internal Vina PyRx 0.10 (https://pyrx.sourceforge.io/ (accessed on 29 November 2023)) for the actual docking process. Subsequently, the outcomes were subjected to visual analysis, facilitated by Pymol 2.3.0 (https://pymol.org/2/ (accessed on 2 December 2023)) for three-dimensional representations. 

### 4.9. Statistical Analysis

Statistical analyses were performed with GraphPad Prism software 9.1. The significance analysis was conducted using a two-tailed unpaired *t*-test. *p* < 0.05 was considered statistically significant (* *p* < 0.05, ** *p* < 0.01, and *** *p* < 0.001).

## 5. Conclusions

In this study, compounds **LJ-41** and **1a**, synthesized from a precursor derived from ICT derivative **1a**, were used to treat CA-46 cells, aiming to identify the target of action for ICT. After proteasomal degradation, the degraded proteins were identified using a quantitative proteomics approach. Through validation of the possible targets, BI-1 was identified as the primary target of ICT. This finding suggests that ICT exerts its anti-tumor effect by interacting with BI-1. Moreover, it was shown that LJ-41 directly binds to BI-1, which degrades the target protein and leads to apoptosis of CA-46 cells through activation of the IRE1 pathway, the ATF6 pathway, and the NRF2-HO-1 pathway. This results in a pronounced antitumor effect. These findings underscore the excellent anti-tumor activity of ICT and **LJ-41** as potential effector molecules targeting BI-1. The study also suggests that in the technique of target discovery using PROTACs combined with differential proteomics, the inclusion of ligands for the protein of interest (POI) in the therapeutic group is a valuable approach for identifying the correct drug target. This research significantly contributes to our understanding of the mechanisms underlying ICT’s anti-tumor properties.

## Figures and Tables

**Table 1:** Composition of the components of ICT-PROTACs.

Compounds	POI Ligand	Linker	E3 Ligand
**3**	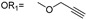	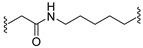	Ledonamide
**4**	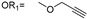	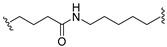	Ledonamide
**5**	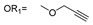	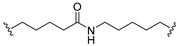	Ledonamide
**6**	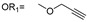	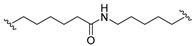	Ledonamide
**7**	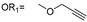	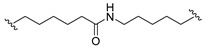	Ledonamide
**8**	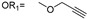	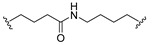	Ledonamide
**9**	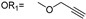	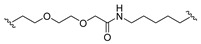	Ledonamide
**10**	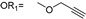	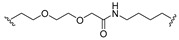	Ledonamide
**11**	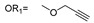	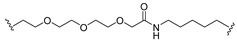	Ledonamide
**12**	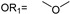	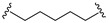	Ledonamide
**13**	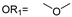	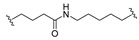	Ledonamide
**14**	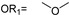	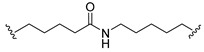	Ledonamide
**15**	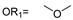	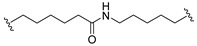	Ledonamide
**16**	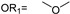	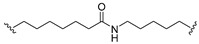	Ledonamide
**17**	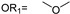	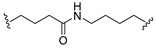	Ledonamide
**18**	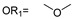	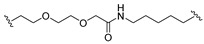	Ledonamide
**19**	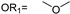	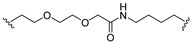	Ledonamide
**20**	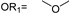	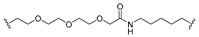	Ledonamide
**21**	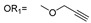	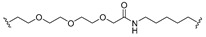	V0
**22**	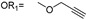	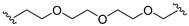	V0
**23**	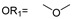	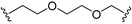	V0
**24**	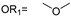	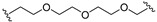	V0

**Table 2:** CA-46 inhibitory activity of the compounds.

Compounds	IC_50_ (μM) ^a^	Compounds	IC_50_ (μM) ^a^	Compounds	IC_50_ (μM) ^a^	Compounds	IC_50_ (μM) ^a^
**ICT**	66.91 ± 0.81	**1a**	48.19 ± 2.31	**1b**	50.68 ± 0.57	**Celastrol ^b^**	0.88 ± 0.14
**3**	5.79 ± 0.38	**4**	6.69 ± 0.34	**5**	7.48 ± 0.26	**6**	13.14 ± 1.61
**7**	>20	**8**	4.60 ± 0.26	**9**	5.81 ± 0.72	**10**	5.52 ± 0.59
**11**	8.71 ± 1.26	**12**	6.92 ± 1.79	**13**	7.46 ± 0.56	**14**	>20
**15**	>20	**16**	9.14 ± 2.53	**17**	5.41 ± 2.11	**18**	>20
**19**	5.82 ± 0.92	**20**	>20	**21**	>20	**22**	>20
**23**	6.09 ± 0.15						

^a^ The blank group was cultured with DMSO treatment. Data were from three independent experiments and are mean ± SD. ^b^ Celastrol for positive control.

**Table 3:** Part of the downregulated differential proteins in the PROTAC group vs. the precursor group.

Gene Name	Accession	Description	Localization	Log_Fold Change	*p*-Value
*NLRP13*	Q86W25	NACHT, LRR, and PYD domains-containing protein 13	cytosol	−1.54	*p* < 0.05
*CXXC1*	Q9P0U4	CXXC-type zinc finger protein 1	cytosol	−1.48	*p* < 0.05
*TECPR2*	O15040	Tectonin beta-propeller repeat-containing protein 2	nucleus	−1.40	*p* < 0.05
*GRIA3*	P42263	Glutamate receptor 3	plasma membrane	−1.25	*p* < 0.05
*TMBIM6*	P55061	Bax inhibitor 1	endoplasmic reticulum	−1.07	*p* < 0.05
*DENND4C*	Q5VZ89	DENN domain-containing protein 4C	cytosol	−0.97	*p* < 0.05
*FSD1*	Q9BTV5	Fibronectin type III and SPRY domain-containing protein 1	nucleus	−0.86	*p* < 0.05
*TMEM18*	Q96B42	Transmembrane protein 18	nucleus	−0.71	*p* < 0.05
*NMD3*	Q96D46	60S ribosomal export protein NMD3	nucleus	−0.68	*p* < 0.05

**Table 4:** Part of the downregulated differential proteins in the PROTAC group vs. the control group.

Gene Name	Accession	Description	Localization	Log_Fold Change	*p*-Value
*NLRP13*	Q86W25	NACHT, LRR, and PYD domains-containing protein 13	cytosol	−1.60	*p* < 0.05
*TECPR2*	O15040	Tectonin beta-propeller repeat-containing protein 2	nucleus	−1.27	*p* < 0.05
*GRIA3*	P42263	Glutamate receptor 3	plasma membrane	−1.13	*p* < 0.05
*NMD3*	Q96D46	60S ribosomal export protein NMD3	nucleus	−0.87	*p* < 0.05
*TMBIM6*	P55061	Bax inhibitor 1	endoplasmic reticulum	−0.87	*p* = 0.051
*NAB2*	Q15742	NGFI-A-binding protein 2	cytosol	−0.77	*p* < 0.05
*UPRT*	Q96BW1	Uracil phosphoribosyltransferase homolog	nucleus	−0.75	*p* < 0.05

## Data Availability

Data is contained within the article and Supplementary Materials.

## Supplementary Materials

The following supporting information can be downloaded at: https://www.mdpi.com/article/10.3390/ijms252312944/s1.

## References

[1] Dong S., Guo X., Han F., He Z., Wang Y. (2022). Emerging role of natural products in cancer immunotherapy. Acta Pharm. Sin. B.

[2] Baell J.B. (2016). Feeling Nature’s PAINS: Natural Products, Natural Product Drugs, and Pan Assay Interference Compounds (PAINS). J. Nat. Prod..

[3] Atanasov A.G., Zotchev S.B., Dirsch V.M., Supuran C.T., Int Nat Prod Sci T. (2021). Natural products in drug discovery: Advances and opportunities. Nat. Rev. Drug Discov..

[4] Wu Y., Yang Y., Wang W., Sun D., Liang J., Zhu M., Li H., Chen L. (2022). PROTAC technology as a novel tool to identify the target of lathyrane diterpenoids. Acta Pharm. Sin. B.

[5] Li Y., Zeng Z.W., Chen D., Gu Z.C., Yan W.L., Yue L.Y., Zhu R.G., Zhao Y.L., Chen L., Zhao Q.J. (2023). Facilitated Drug Repurposing with Artemisinin-Derived PROTACs: Unveiling PCLAF as a Therapeutic Target. J. Med. Chem..

[6] Lei S.W., Cui G., Leung G.P.H., Luk S.C.W., Hoi M.P.M., Wang L., Mahady G.B., Lee S.M.Y. (2015). Icaritin protects against oxidative stress-induced injury in cardiac H9c2 cells via Akt/Nrf2/HO-1 and calcium signalling pathways. J. Funct. Foods.

[7] Zhang W., Xing B., Yang L., Shi J., Zhou X. (2015). Icaritin Attenuates Myocardial Ischemia and Reperfusion Injury Via An-ti-Inflammatory and Anti-Oxidative Stress Effects in Rats. Am. J. Chin. Med..

[8] Yao D., Xie X.-H., Wang X.-L., Wan C., Lee Y.-W., Chen S.-H., Pei D.-Q., Wang Y.-X., Li G., Qin L. (2012). Icaritin, an Exogenous Phytomolecule, Enhances Osteogenesis but Not Angiogenesis-An In Vitro Efficacy Study. PLoS ONE.

[9] Wu T., Shu T., Kang L., Wu J., Xing J., Lu Z., Chen S., Lv J. (2017). Icaritin, a novel plant-derived osteoinductive agent, enhances the osteogenic differentiation of human bone marrow- and human adipose tissue-derived mesenchymal stem cells. Int. J. Mol. Med..

[10] Li Y.-Y., Huang N.-Q., Feng F., Li Y., Luo X.-M., Tu L., Qu J.-Q., Xie Y.-M., Luo Y. (2020). Icaritin Improves Memory and Learning Ability by Decreasing BACE-1 Expression and the Bax/Bcl-2 Ratio in Senescence-Accelerated Mouse Prone 8 (SAMP8) Mice. Evid. Based Complement. Altern. Med..

[11] Wu H., Liu X., Gao Z.-Y., Lin M., Zhao X., Sun Y., Pu X.-P. (2021). Icaritin Provides Neuroprotection in Parkinson’s Disease by Attenuating Neuroinflammation, Oxidative Stress, and Energy Deficiency. Antioxidants.

[12] Zhu S., Xing C., Zhang G., Peng H., Wang Z. (2021). Icaritin induces cellular senescence by accumulating the ROS production and regulation of the Jak2/Stat3/p21 pathway in imatinib-resistant, chronic myeloid leukemia cells. Am. J. Transl. Res..

[13] Yang X.-J., Xi Y.-M., Li Z.-J. (2019). Icaritin: A Novel Natural Candidate for Hematological Malignancies Therapy. BioMed Res. Int..

[14] Yu Z., Guo J., Hu M., Gao Y., Huang L. (2020). Icaritin Exacerbates Mitophagy and Synergizes with Doxorubicin to Induce Immunogenic Cell Death in Hepatocellular Carcinoma. Acs Nano.

[15] Li H., Liu Y., Jiang W., Xue J., Cheng Y., Wang J., Yang R., Zhang X. (2021). Icaritin promotes apoptosis and inhibits proliferation by down-regulating AFP gene expression in hepatocellular carcinoma. Bmc Cancer.

[16] Wang X., Zheng N., Dong J., Wang X., Liu L., Huang J. (2017). Estrogen receptor-alpha 36 is involved in icaritin induced growth inhibition of triple-negative breast cancer cells. J. Steroid Biochem. Mol. Biol..

[17] Yin L., Qi X.-W., Liu X.-Z., Yang Z.-Y., Cai R.-L., Cui H.-J., Chen L., Yu S.-C. (2020). Icaritin enhances the efficacy of cetuximab against triple-negative breast cancer cells. Oncol. Lett..

[18] Wu X., Long X., Yang C., Chen H., Sharkey C., Rashid K., Hu M., Liu Y., Huang Q., Chen Q. (2020). Icaritin reduces prostate cancer progression via inhibiting high-fat diet-induced serum adipokine in TRAMP mice model. J. Cancer.

[19] Li X., Zhang W., Liang L., Duan X., Deng J., Zhou Y. (2020). Natural product-derived icaritin exerts anti-glioblastoma effects by positively modulating estrogen receptor beta. Exp. Ther. Med..

[20] He J., Wang Y., Duan F., Jiang H., Chen M.-F., Tang S.-Y. (2010). Icaritin Induces Apoptosis of HepG2 Cells via the JNK1 Signaling Pathway Independent of the Estrogen Receptor. Planta Medica.

[21] Tao H., Liu M., Wang Y., Luo S., Xu Y., Ye B., Zheng L., Meng K., Li L. (2021). Icaritin Induces Anti-tumor Immune Responses in Hepatocellular Carcinoma by Inhibiting Splenic Myeloid-Derived Suppressor Cell Generation. Front. Immunol..

[22] Zhang M., Li X., Zhang Y., Zhou K. (2010). Bax inhibitor-1 mediates apoptosis-resistance in human nasopharyngeal carcinoma cells. Mol. Cell. Biochem..

[23] Li X.-Y., Lai Y.-K., Zhang J.-F., Luo H.-Q., Zhang M.-H., Zhou K.-Y., Kung H.-F. (2011). Lentivirus-Mediated RNA Interference Targeting Bax Inhibitor-1 Suppresses Ex Vivo Cell Proliferation and In Vivo Tumor Growth of Nasopharyngeal Carcinoma. Hum. Gene Ther..

[24] Grzmil M., Thelen P., Hemmerlein B., Schweyer S., Voigt S., Mury D., Burfeind P. (2003). Bax inhibitor-1 is overexpressed in prostate cancer and its specific down-regulation by RNA interference leads to cell death in human prostate carcinoma cells. Am. J. Pathol..

[25] Szegezdi E., Logue S.E., Gorman A.M., Samali A. (2006). Mediators of endoplasmic reticulum stress-induced apoptosis. Embo Rep..

[26] Dong Y., Chen H., Gao J., Liu Y., Li J., Wang J. (2019). Molecular machinery and interplay of apoptosis and autophagy in coronary heart disease. J. Mol. Cell. Cardiol..

[27] Zhang F., Wu Z., Chen P., Zhang J., Wang T., Zhou J., Zhang H. (2020). Discovery of a new class of PROTAC BRD4 degraders based on a dihydroquinazolinone derivative and lenalidomide/pomalidomide. Bioorganic Med. Chem..

[28] Hu J., Hu B., Wang M., Xu F., Miao B., Yang C.-Y., Wang M., Liu Z., Hayes D.F., Chinnaswamy K. (2019). Discovery of ERD-308 as a Highly Potent Proteolysis Targeting Chimera (PROTAC) Degrader of Estrogen Receptor (ER). J. Med. Chem..

[29] Li B., Ran T., Chen H. (2023). 3D based generative PROTAC linker design with reinforcement learning. Brief. Bioinform..

[30] Li W., Gao C., Zhao L., Yuan Z., Chen Y., Jiang Y. (2018). Phthalimide conjugations for the degradation of oncogenic PI3K. Eur. J. Med. Chem..

[31] Pei H., Peng Y., Zhao Q., Chen Y. (2019). Small molecule PROTACs: An emerging technology for targeted therapy in drug discovery. Rsc Adv..

[32] Bondeson D.P., Smith B.E., Burslem G.M., Buhimschi A.D., Hines J., Jaime-Figueroa S., Wang J., Hamman B.D., Ishchenko A., Crews C.M. (2018). Lessons in PROTAC Design from Selective Degradation with a Promiscuous Warhead. Cell Chem. Biol..

[33] Huang H.-T., Dobrovolsky D., Paulk J., Yang G., Weisberg E.L., Doctor Z.M., Buckley D.L., Cho J.-H., Ko E., Jang J. (2018). A Chemoproteomic Approach to Query the Degradable Kinome Using a Multi-kinase Degrader. Cell Chem. Biol..

[34] Zhou Y., Xiao Y. (2018). Target Identification of Bioactive Natural Products. Acta Chim. Sin..

[35] Li B., Qi Z.-P., He D.-L., Chen Z.-H., Liu J.-Y., Wong M.-W., Zhang J.-W., Xu E.-P., Shi Q., Cai S.-L. (2021). NLRP7 deubiquitination by USP10 promotes tumor progression and tumor-associated macrophage polarization in colorectal cancer. J. Exp. Clin. Cancer Res..

[36] Ripka S., Riedel J., Neesse A., Griesmann H., Buchholz M., Ellenrieder V., Moeller F., Barth P., Gress T.M., Michl P. (2010). Glutamate Receptor GRIA3-Target of CUX1 and Mediator of Tumor Progression in Pancreatic Cancer. Neoplasia.

[37] Zhang Z.J., Zhang Y.H., Qin X.J., Wang Y.X., Fu J. (2020). Circular RNA circDENND4C facilitates proliferation, migration and glycolysis of colorectal cancer cells through miR-760/GLUT1 axis. Eur. Rev. Med. Pharmacol. Sci..

[38] Tanaka R., Ishiyama T., Uchihara T., Inadome Y., Iijima T., Morishita Y., Kano J., Goya T., Noguchi M. (2006). Expression of the Bax inhibitor-1 gene in pulmonary adenocarcinoma. Cancer.

[39] Lu B., Li Y., Li H., Zhang Y., Xu J., Ren L., Fu S., Zhou Y. (2015). Bax inhibitor-1 is overexpressed in non-small cell lung cancer and promotes its progression and metastasis. Int. J. Clin. Exp. Pathol..

[40] Grzmil M., Kaulfuss S., Thelen P., Hemmerlein B., Schweyer S., Obenauer S., Kang T.W., Burfeind P. (2006). Expression and functional analysis of Bax inhibitor-I in human breast cancer cells. J. Pathol..

[41] Hsu C.-F., Sui C.-L., Wu W.-C., Wang J.-J., Yang D.H.A., Chen Y.-C., Yu W.C.Y., Chang H.-S. (2011). Klf10 induces cell apoptosis through modulation of BI-1 expression and Ca2+ homeostasis in estrogen-responding adenocarcinoma cells. Int. J. Biochem. Cell Biol..

[42] Simpson L.M., Glennie L., Brewer A., Zhao J.-F., Crooks J., Shpiro N., Sapkota G.P. (2022). Target protein localization and its impact on PROTAC-mediated degradation. Cell Chem. Biol..

[43] Almen M.S., Jacobsson J.A., Shaik J.H.A., Olszewski P.K., Cedernaes J., Alsio J., Sreedharan S., Levine A.S., Fredriksson R., Marcus C. (2010). The obesity gene, TMEM18, is of ancient origin, found in majority of neuronal cells in all major brain regions and associated with obesity in severely obese children. Bmc Med. Genet..

[44] Lisbona F., Rojas-Rivera D., Thielen P., Zamorano S., Todd D., Martinon F., Glavic A., Kress C., Lin J.H., Walter P. (2009). BAX Inhibitor-1 Is a Negative Regulator of the ER Stress Sensor IRE1 alpha. Mol. Cell.

[45] Bailly-Maitre B., Belgardt B.F., Jordan S.D., Coornaert B., von Freyend M.J., Kleinridders A., Mauer J., Cuddy M., Kress C.L., Willmes D. (2010). Hepatic Bax Inhibitor-1 Inhibits IRE1 alpha and Protects from Obesity-associated Insulin Resistance and Glucose Intolerance. J. Biol. Chem..

[46] Xu C., Xu W., Palmer A.E., Reed J.C. (2008). BI-1 regulates endoplasmic reticulum Ca2+ Homeostasis downstream of bcl-2 family proteins. J. Biol. Chem..

[47] Kim H.-R., Lee G.-H., Cho E.Y., Chae S.-W., Ahn T., Chae H.-J. (2009). Bax inhibitor 1 regulates ER-stress-induced ROS accumulation through the regulation of cytochrome P450 2E1. J. Cell Sci..

[48] Lee G.-H., Oh K.-J., Kim H.-R., Han H.-S., Lee H.-Y., Park K.-G., Nam K.-H., Koo S.-H., Chae H.-J. (2016). Effect of BI-1 on insulin resistance through regulation of CYP2E1. Sci. Rep..

[49] Lee G.-H., Kim H.-R., Chae H.-J. (2012). Bax inhibitor-1 regulates the expression of P450 2E1 through enhanced lysosome activity. Int. J. Biochem. Cell Biol..

[50] Lee G.-H., Kim H.-K., Chae S.-W., Kim D.-S., Ha K.-C., Cuddy M., Kress C., Reed J.C., Kim H.-R., Chae H.-J. (2007). Bax inhibitor-1 regulates endoplasmic reticulum stress-associated reactive oxygen species and heme oxygenase-1 expression. J. Biol. Chem..

[51] Cencini E., Calomino N., Sicuranza A., Gozzetti A., Fabbri A., Bocchia M. (2024). Are small lymphocytic lymphoma and chronic lymphocytic leukemia the same disease? The unsolved dilemma. J. Chemother..

[52] Cencini E., Sicuranza A., Fabbri A., Ferrigno I., Rigacci L., Cox M.C., Raspadori D., Bocchia M. (2019). Study of gene polymorphisms as predictors of treatment efficacy and toxicity in patients with indolent non-hodgkin lymphomas and mantle cell lymphoma receiving bendamustine and rituximab. Br. J. Haematol..

