# Supplementary material for: Advanced PROTAC and Quantitative Proteomics Strategy Reveals Bax Inhibitor-1 as a Critical Target of Icaritin in Burkitt Lymphoma
Source: Int J Mol Sci. 2024 Dec 2;25(23):12944. doi: 10.3390/ijms252312944 (PMC11641208; doi:10.3390/ijms252312944)
Supplement: Supplementary file 1 [file ijms-25-12944-s001.zip › ijms-3332713-supplementary.pdf]

Supporting Information

Contents

Chemical Synthesis 2

Supplementary Figure 1：Chemical structure of POI ligand 2

Supplementary Figure 2: List of final compound structures 2

General information 3

Synthesis of compounds of Icaritin-PROTACs 5

1H and 13C 1D NMR spectra for compounds 1a/b and compounds 3-24 42

Supplementary Figure 3: 1H and 13C spectra of compounds 1a 42

Supplementary Figure 4: 1H and 13C spectra of compound 1b 43

Supplementary Figure 5: 1H and 13C spectra of compounds 3 44

Supplementary Figure 6: 1H and 13C spectra of compounds 4 45

Supplementary Figure 7: 1H and 13C spectra of compounds 5 46

Supplementary Figure 8: 1H and 13C spectra of compounds 6 47

Supplementary Figure 9: 1H and 13C spectra of compounds 7 48

Supplementary Figure 10: 1H and 13C spectra of compounds 8 49

Supplementary Figure 11: 1H and 13C spectra of compounds 9 50

Supplementary Figure 12: 1H and 13C spectra of compounds 10 51

Supplementary Figure 13: 1H and 13C spectra of compounds 11 52

Supplementary Figure 14: 1H and 13C spectra of compounds 12 53

Supplementary Figure 15: 1H and 13C spectra of compounds 13 54

Supplementary Figure 16: 1H and 13C spectra of compounds 14 55

Supplementary Figure 17: 1H and 13C spectra of compounds 15 56

Supplementary Figure 18: 1H and 13C spectra of compounds 16 57

Supplementary Figure 19: 1H and 13C spectra of compounds 17 58

Supplementary Figure 20: 1H and 13C spectra of compounds 18 59

Supplementary Figure 21: 1H and 13C spectra of compounds 19 60

Supplementary Figure 22: 1H and 13C spectra of compounds 20 61

Supplementary Figure 23: 1H and 13C spectra of compounds 21 62

Supplementary Figure 24: 1H and 13C spectra of compounds 22 63

Supplementary Figure 25: 1H and 13C spectra of compounds 23 64

Supplementary Figure 26: 1H and 13C spectra of compounds 24 65

Biological experiment 66

Chemical Synthesis

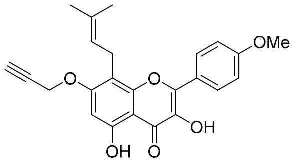

Compound 1a

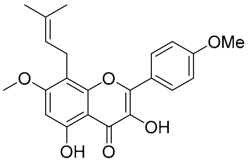

Compound 1b

Supplementary Figure 1：Chemical structure of POI ligand

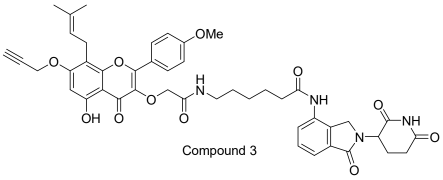

Compound 3

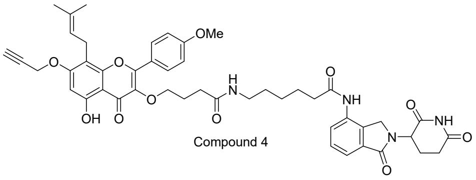

Compound 4

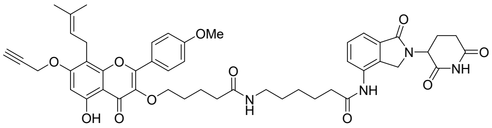

Compound 5

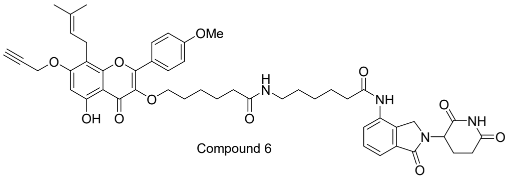

Compound 6

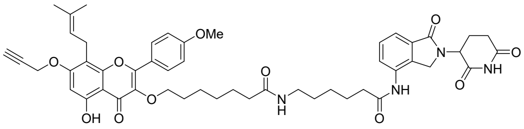

Compound 7

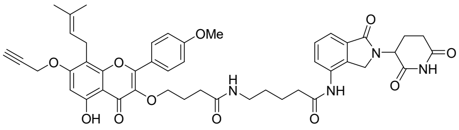

Compound 8

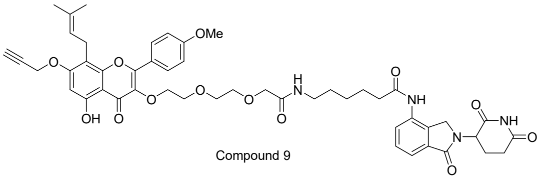

Compound 9

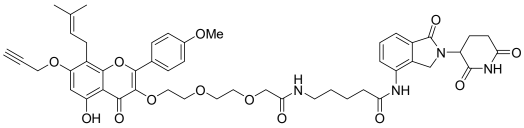

Compound 10 (LJ-41)

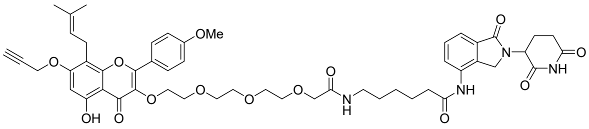

Compound 11

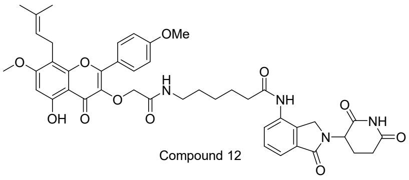

Compound 12

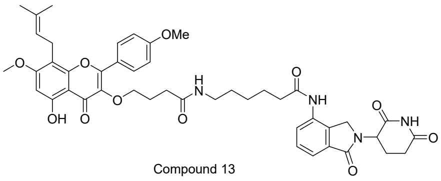

Compound 13

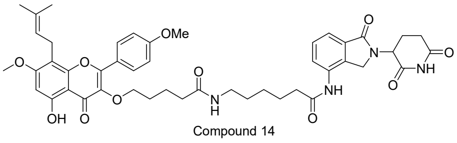

Compound 14

Supplementary Figure 2: List of final compound structures

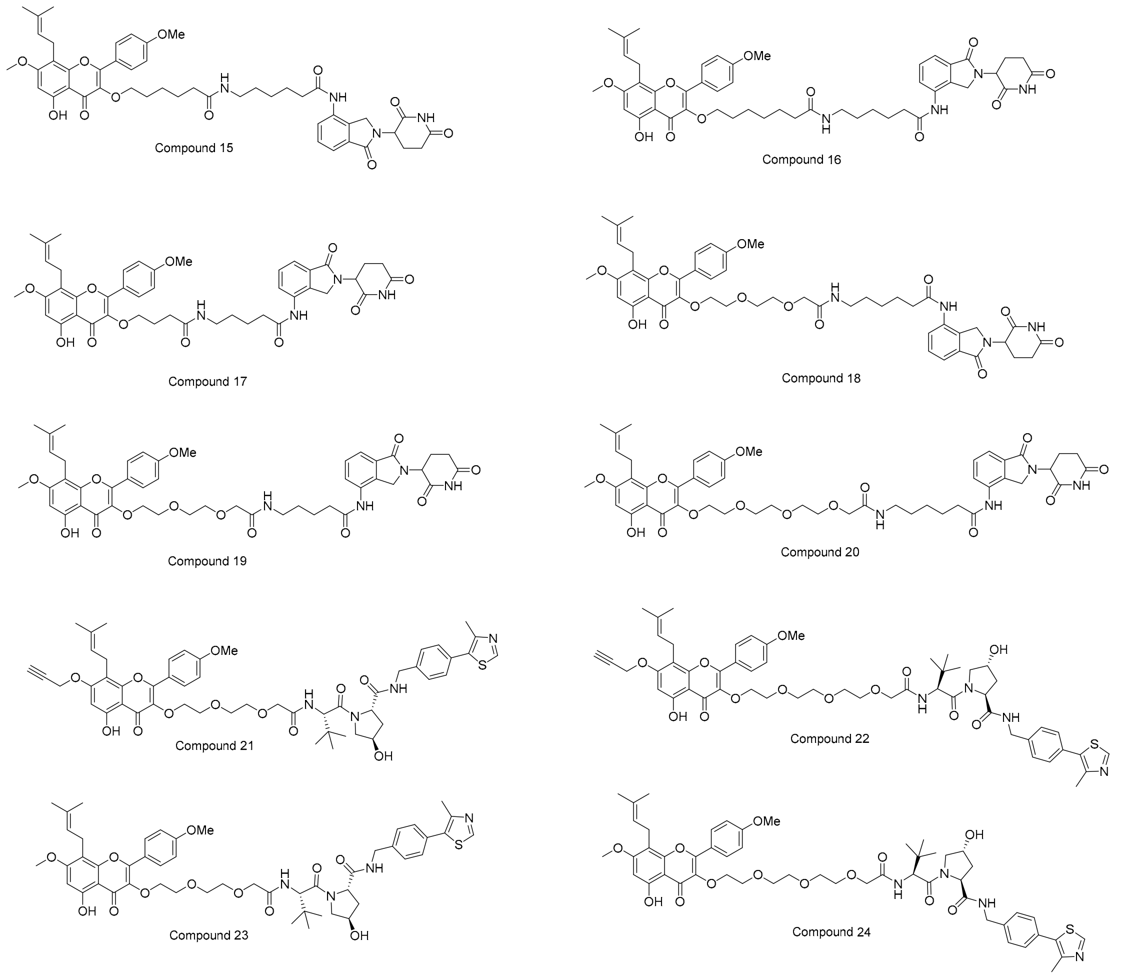

Supplementary Figure 2: List of final compound structures

General information

ESI-MS was measured on an ultra-high performance (pressure) liquid chromatography-quadrupole time-of-flight mass spectrometer (Agilent, 1290-6545); one-dimensional nuclear magnetic resonance (NMR) hydrogen spectra and carbon spectra were measured on a Bruker Avance III-400MHz and Bruker Avance III-500MHz NMR instrument (Bruker, Switzerland), with TMS as the internal standard and δ The common equipments used in the experiments were: rotary evaporator (N-1100, Shanghai Ailang Instrument Co., Ltd.), electrothermal thermostatic water bath (DK-S24, Shanghai Senxin Experimental Equipment Co., Ltd.), collector-type thermostatic magnetic stirrer (DF-101S, Gongyi Iuhua Instrument Co., Ltd.), electronic balance (MP3002, Shanghai Precision Instrument Co., Ltd.), analytical balance (AR301, Shanghai Precision Instrument Co., Ltd.), and analytical balance (AR301, Shanghai Precision Instrument Co. Ltd.), analytical balance (AR224CN, OHAUS Instrument Co., Ltd.), circulating water vacuum pump (SHZ-D (III), Gongyi Iohua Instrument Co., Ltd.), large rotary evaporator (R2003KE, Gongyi Iohua

Instrument Co., Ltd.), and glass chromatography columns (various specifications, Beijing Xinweiell Glass Instrument Co., Ltd.).

The common organic solvents used in the experiments were pyridine, N,N-dimethylformamide and acetone, all of which were dewatered with activated molecular sieves, and the rest of the organic solvents were untreated if not otherwise specified. The organic solvents and reaction reagents used in the experiments were purchased from Sinopharm Group Chemical Reagent Co, Ltd, Shanghai McLean Biochemical Science and Technology Co, Ltd, Xilong Chemical Co, Ltd, Guangdong Guanghua Science and Technology Co, Ltd, Shanghai Aladdin Biochemical Science and Technology Co, Ltd, and West Asia Chemical Technology (Shandong) Co, Ltd. Silica gel (200-300 mesh) for chromatography was produced by Qingdao Ocean Chemical Co., Ltd; silica gel (GF254) for PTLC (Preparative Thin Layer Chromatography) was produced by Qingdao Ocean Chemical Co. The color developer was 10% ethanol sulfate, which was properly heated after dipping.

Synthesis of compounds of Icaritin-PROTACs

1.1 Synthesis of icaritin Derivatives

Supplementary Scheme 1. Synthesis of POI ligands

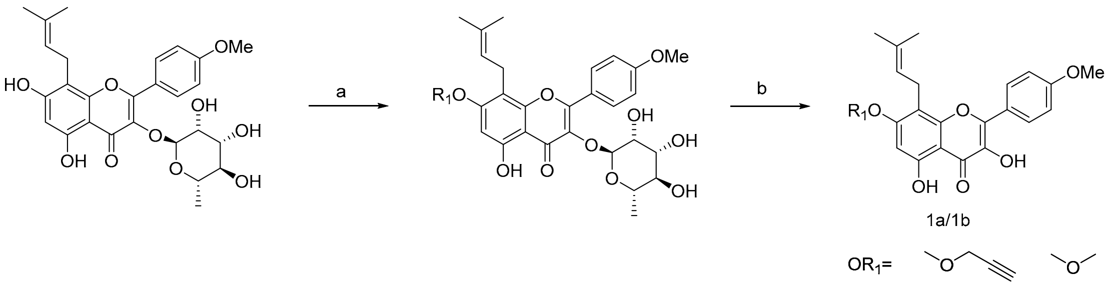

[a]Reagents and conditions: (a) Corresponding bromoalkanes, K_2CO_3, Me_2CO, reflux; (b) concentrated sulfuric acid, 60°C, 8h

1.1.1 1.5-hydroxy-2-(4-methoxyphenyl)-8-(3-methylbut-2-en-1-yl)-7-(prop-2-yn-1-yloxy)-3-(((2S,3R,4R,5R,6S)-3,4,5-trihydroxy-6-methyltetrahydro-2H-pyran-2-yl)oxy)-4H-chromen-4-one (compound T)

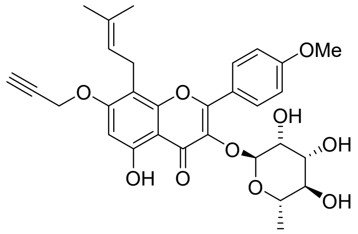

Compound T

General synthesis procedure for ***compound T***. Icariin II (3 g, 5.83 mmol) was dissolved in dry acetone (50 mL), K_2CO_3 (2.01 g, 14.58 mmol) was added, and after stirring for fifteen minutes at room temperature, bromopropargyl (754 μL, 8.75 mmol) was added, and the reaction was monitored by TLC by increasing the temperature to 55 °C at reflux, and the reaction was stopped after about 8 hours. Reaction. The reaction was quenched by adding ice water to the reaction flask, adjusting the pH to 6~7 with 4N HCl, pumping filtration, washing the filter cake with water for three times, drying, and then column chromatography (DCM : MeOH = 50:1) to obtain the **compound T**. The reaction was carried out at room temperature for fifteen minutes and then refluxed.

Compound T（Light yellow solid，3.1 g，96.3%）. ^{1}H NMR (500 MHz, DMSO-d_6) δ: 12.64 (s, 1H), 7.88 (d, 2H), 7.13 (d, 2H), 6.64 (s, 1H), 5.28 (s, 1H), 5.13 (t, J = 7.1, 1.7 Hz, 1H), 4.99 (d, J = 2.4 Hz, 2H), 4.00 (s, 1H), 3.86 (s, 3H), 3.68 (t, J = 2.4 Hz, 1H), 3.51-3.39 (m, 4H), 3.17 – 3.06 (m, 3H), 1.69 (s, 3H), 1.62 (s, 3H), 0.79 (d, J = 6.0 Hz, 3H). ^{13}C NMR (126 MHz, DMSO) δ 178.75, 161.87, 160.80, 159.73, 157.74, 153.37, 135.04, 131.96, 130.97, 122.72, 122.22, 114.57, 108.18, 105.59, 102.50, 96.88, 79.33, 78.99, 71.56, 71.15, 70.75, 70.51, 57.14, 55.98, 25.91, 21.70, 18.27, 17.92. HRMS: calculated for $C_{30}H_{32}O_{10}$ $[M + H]^+$,553.2068.; found, 553.2066.

1.1.2 3,5-dihydroxy-2-(4-methoxyphenyl)-8-(3-methylbut-2-en-1-yl)-7-(prop-2-yn-1-yloxy)-

4H-chromen-4-one (Compound 1a)

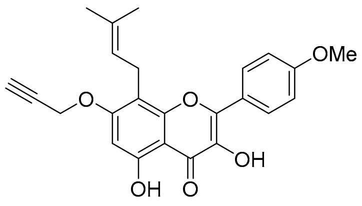

Compound 1a

*General synthesis procedure for **compound 1a***. **Compound T** (3 g, 5.43 mmol) was added to a mixed solution of ethanol and water (V : V = 1:1) (100 mL), and concentrated sulfuric acid (9.5 mL) was added slowly dropwise to the suspension under stirring at room temperature, and after addition, the reaction was warmed up to 60 °C and refluxed for about 10 hours, and the reaction was followed by TLC to the end. The reaction was left to cool, and the pH was adjusted to 6~7 with saturated NaHCO3 solution and 4M NaOH solution under ice bath, the suspension was filtered, and the filter cake was washed with water for three times, and the dried crude was purified by column chromatography (PE : DCM : MeOH= 200 : 50 : 1) to obtain **Compound 1a** for the next step of the reaction.

Compound 1a (bright yellow solid, 84.7%). ^{1}H NMR (500 MHz, DMSO-d_6) δ: 12.50 (s, 1H), 9.63 (s, 1H), 8.14 (d, 2H), 7.13 (d, 2H), 6.60 (s, 1H), 5.15 (t, 1H), 4.97 (d, 2H), 3.85 (s, 3H), 3.67 (t, 1H), 3.46 (d, J = 7.0 Hz, 2H), 1.76 (s, 3H), 1.63 (s, 3H). ^{13}C NMR (126 MHz, DMSO) δ 176.98, 161.10, 160.47, 159.21, 153.08, 147.31, 136.56, 131.94, 129.78, 123.88, 122.42, 114.60, 107.91, 104.51, 96.25, 79.26, 79.10, 57.08, 55.87, 25.93, 21.74, 18.31. HRMS: calculated for $C_{24}H_{22}O_6$ [M + H] $^+$, 407.1489.; found, 407.1488.

1.1.3 5-hydroxy-7-methoxy-2-(4-methoxyphenyl)-8-(3-methylbut-2-en-1-yl)-3-(((2S,3R,4R,5R,6S)-3,4,5-trihydroxy-6-methyltetrahydro-2H-pyran-2-yl)oxy)-4H-chromen-4-one (Compounds I)

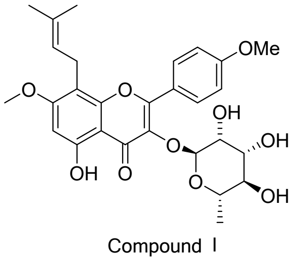

Compound I

1.1.4 3,5-dihydroxy-7-methoxy-2-(4-methoxyphenyl)-8-(3-methylbut-2-en-1-yl)-4H-chromen-4-one (Compounds 1b)

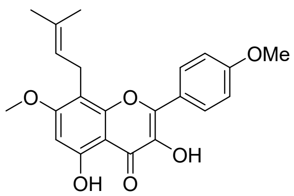

Compound 1b

Compounds I and **compounds 1b** were synthesized with reference to T and 1a.

Intermediate I (yellow powder, 2.96 g, 86.1 %). ^{1}H NMR (400 MHz, DMSO-d_6) δ: 12.67 (s, 1H), 7.89 (d, J = 1.8 Hz, 2H), 7.12 (d, 2H), 6.58 (s, 1H), 5.28 (s, 1H), 5.11 (t, J = 6.0 Hz, 1H), 5.00 (d, J = 4.5 Hz, 1H), 4.74 (s, 1H), 4.67 (s, 1H), 4.11 (q, J = 5.3 Hz, 1H), 4.00 (s, 1H), 3.91 (s, 3H), 3.86 (d, J = 1.6 Hz, 3H), 3.52 – 3.46 (m, 1H), 3.21 – 3.09 (m, 2H), 3.10 – 3.06 (m, 1H), 1.68 (s, 3H), 1.62 (s, 3H), 0.79 (d, J = 5.9 Hz, 3H). ^{13}C NMR (101 MHz, DMSO) δ: 178.73, 162.96, 161.83, 160.12, 157.61, 153.26, 134.91, 131.82, 130.94, 122.76, 122.39, 114.54, 107.50, 105.07, 102.48, 95.66, 71.57, 71.15, 70.77, 70.53, 56.96, 55.96, 25.88, 21.61, 18.19, 17.92. HRMS: calculated for $C_{28}H_{32}O_{10}$ [M + H] $^{+}$, 529.2068.; found, 529.2069.

Compound 1b (yellow powder solid, 1.85 g, 86.4%). ^{1}H NMR (500 MHz, DMSO-d_6) δ :12.52 (s, 1H), 9.58 (s, 1H), 8.13 (d, J = 8.5 Hz, 2H), 7.13 (d, J = 8.5 Hz, 2H), 6.54 (s, 1H), 5.13 (t, 1H), 3.89 (s, 3H), 3.85 (s, 3H), 3.45 (d, J = 6.9 Hz, 2H), 1.75 (s, 3H), 1.63 (s, 3H). ^{13}C NMR (126 MHz, DMSO) δ 176.98, 162.62, 161.05, 159.58, 153.00, 147.12, 136.43, 131.79, 129.74, 123.94, 122.62, 114.59, 107.24, 104.00, 95.07, 56.87, 55.87, 25.89, 21.65, 18.23. HRMS: calculated for $C_{28}H_{32}O_{10}$ [M + H] $^{+}$, 383.1489.; found, 383.1487.

Supplementary Scheme 2. Synthesis of intermediate s3-s6

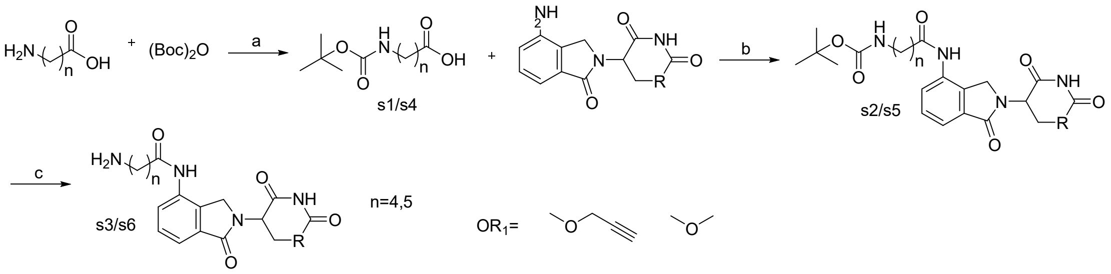

^{a}Reagents and conditions: (a) 1M NaOH, dioxane/H2O, 0°C-RT,3h; (b) HATU, DIPEA, DMF, RT; (c) TFA, DCM, RT

1.2 preparation of E3 ligand (Synthesis of intermediates S1~S6

1.2.1 6-((tert-butoxycarbonyl)amino)hexanoic acid（s1）

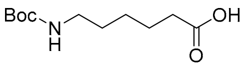

s1

*General synthesis procedure for **s1***. Under an ice bath, 6-aminohexanoic acid (1 g, 7.62 mmol) was dissolved in a mixed solution of Dioxane/H2O (V:V=2:1) (25 mL), 1M NaOH solution (7.65

mL) was added slowly, stirred well, Boc_2O (1.99 mL, 8.66 mmol) was added, removed from the ice bath, and the reaction was warmed up slowly to room temperature, with intermittent recharge of the 1M NaOH solution, so that the pH of the reaction solution was maintained at 8~10, TLC monitoring, the reaction was stopped after about 4 h. 1M HCl solution adjusted the pH to about 2, EA extraction (5 mL × 3), the organic layers were combined, the organic phase was washed with 3% aqueous citric acid and saturated NaCl sequentially, anhydrous Na_2SO_4 drying, filtration and concentration under reduced pressure to obtain **s1** (colorless transparent liquid, 1.49 g, 84.7%), without purification, directly used in the next reaction.

1.2.2 tert-butyl (6-((2-(2,6-dioxopiperidin-3-yl)-1-oxoisoindolin-4-yl)amino)-6-oxohexyl)carbamate (s2)

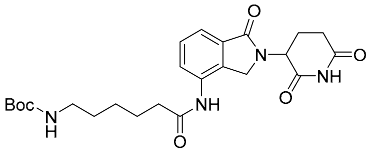

s2

*General synthesis procedure for **s2***. Compound **s1** (1.49 g, 6.44 mmol) was dissolved in dry DMF (20 mL) solution, DIPEA (1.745 l, 10.6 mmol), HATU (1.60 g, 4.22 mmol) were added sequentially at room temperature with stirring, and after the formation of reactive ester was monitored by TLC, lenalidomide (912 mg,3.52 mmol) was added and the reaction was monitored overnight at room temperature by TLC until completion. The reaction was quenched by adding ice water to the reaction solution, a white solid was precipitated, filtered, and the filter cake was washed three times with water and three times with ether, and then dried under vacuum. The crude product was purified by column chromatography (DCM : MeOH= 50 : 1) to give the intermediate **s2**.

Intermediate **s2** (white solid, 1.23 g, 74.1%). ^{1}H NMR (500 MHz, DMSO-d_6) δ : 11.03 (s, 1H), 9.77 (s, 1H), 7.82 (d, J = 7.4, 1.7 Hz, 1H), 7.53 – 7.47 (m, 2H), 6.79 (t, J = 5.8 Hz, 1H), 5.15 (dd, J = 13.3, 5.2 Hz, 1H), 4.38 (q, 2H), 2.97 – 2.85 (m, 3H), 2.62 (d, J = 15.7, 3.1 Hz, 1H), 2.41 – 2.29 (m, 3H), 2.08 – 1.98 (m, 1H), 1.64 – 1.56 (m, 2H), 1.43 – 1.34 (m, 13H). ^{13}C NMR (126 MHz, DMSO) δ 173.29, 171.77, 171.51, 168.29, 156.05, 134.27, 134.15, 133.13, 129.06, 125.69, 119.42, 77.77, 52.02, 46.96, 36.24, 34.09, 31.68, 29.78, 28.74, 26.47, 25.31, 23.11.

1.2.3 6-((2-(2,6-dioxopiperidin-3-yl)-1-oxoisoindolin-4-yl)amino)-6-oxohexan-1-aminium 2,2,2-trifluoroacetate (s3)

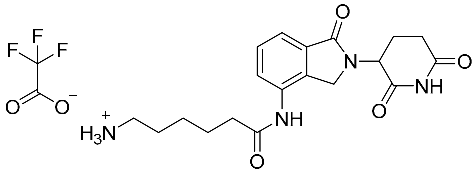

s3

*General synthesis procedure for **s3***. Intermediate s2 (1 g, 2.12 mmol) was dissolved in a mixed solution (10 mL) of DCM/MeOH (V:V=3:1), TFA (3 mL) was added dropwise under an ice bath, and after the addition, it was slowly moved to room temperature for the reaction, and the reaction was monitored by TLC, and the reaction was complete for about 12 hours. After the reaction was stopped, it was concentrated under reduced pressure to obtain the intermediate **s3** (white solid, 988 mg, 96.0%), which was used directly in the next reaction without purification.

Intermediate **s3**. 1H NMR (400 MHz, DMSO-d_6) δ: 11.04 (s, 1H), 9.85 (s, 1H), 7.83 (s, 1H), 7.75 (s, 3H), 7.50 (s, 2H), 5.16 (d, J = 13.3, 4.1 Hz, 1H), 4.35 (q, 2H), 3.00 – 2.88 (m, 1H), 2.79 (s, 2H), 2.61 (d, J = 17.2 Hz, 1H), 2.43 – 2.28 (m, 3H), 2.03 (s, 1H), 1.58 (d, J = 23.3, 8.4, 7.9 Hz, 4H), 1.35 (s, 2H). ^{13}C NMR (101 MHz, DMSO) δ: 173.35, 171.63, 171.55, 168.28, 158.62, 158.32, 158.01, 134.25, 134.09, 133.12, 129.08, 125.65, 119.45, 119.24, 51.95, 46.91, 39.18, 35.98, 31.68, 27.34, 25.97, 25.02, 23.11.

1.2.4 5-((tert-butoxycarbonyl)amino)pentanoic acid (s4)

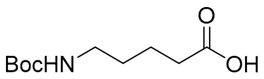

s4

1.2.5 tert-butyl (5-((2-(2,6-dioxopiperidin-3-yl)-1-oxoisoindolin-4-yl)amino)-5-oxopentyl)carbamate (s5)

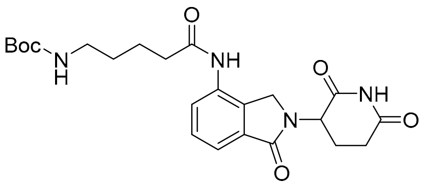

s5

1.2.6 5-((2-(2,6-dioxopiperidin-3-yl)-1-oxoisoindolin-4-yl)amino)-5-oxopentan-1-aminium 2,2,2-trifluoroacetate (s6)

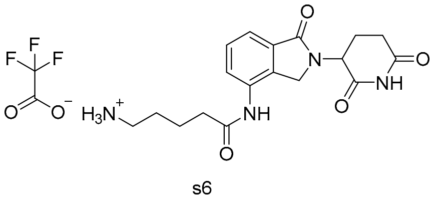

s6

Intermediates **s4~s6** were synthesized similarly to s1~s3.

Intermediate **s4** (anhydrous clear-like liquid, 87.4%).

Intermediate **s5** (white solid, 68.9%).^{1}H NMR (500 MHz, DMSO-d_6) δ: 11.03 (s, 1H), 9.78 (s, 1H), 7.82 (d, J = 7.2, 1.7 Hz, 1H), 7.53 – 7.47 (m, 2H), 6.82 (t, J = 5.7 Hz, 1H), 5.15 (dd, J = 13.3, 5.1 Hz, 1H), 4.38 (q, 2H), 2.97 – 2.88 (m, 3H), 2.61 (d, J = 17.3, 4.4, 2.3 Hz, 1H), 2.40 – 2.30 (m, 3H), 2.07 – 2.00 (m, 1H), 1.62 – 1.54 (m, 2H), 1.46 – 1.40 (m, 2H), 1.37 (s, 9H). ^{13}C NMR (126

MHz, DMSO) δ: 173.29, 171.72, 171.50, 168.29, 156.06, 134.26, 134.15, 133.13, 129.07, 125.69, 119.44, 77.81, 52.01, 46.95, 35.91, 31.68, 29.58, 28.74, 23.12, 22.91.

Intermediate **s6** (white solid, 93.0%).

1.3 Synthesis of end-product compounds 3~8, compounds 12~17

Supplementary Scheme 3. Synthesis of intermediates 3a/b~12a/b

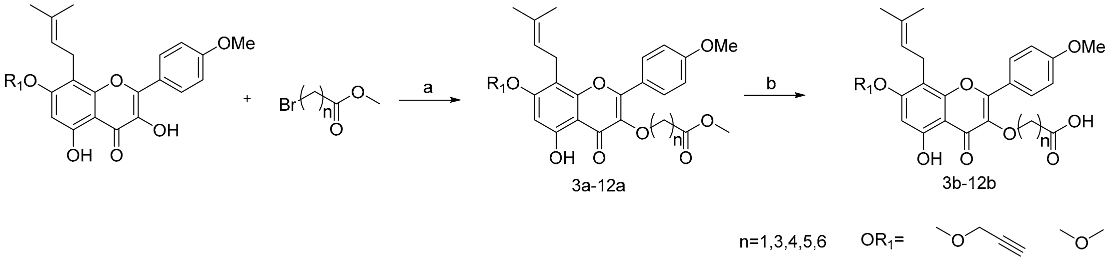

^{a}Reagents and conditions: (a)K_2CO_3, Me_2CO, reflux; (b)LiOH, THF, MeOH;

1.3.1 Synthesis of intermediates 3a~12a

1.3.1.1 methyl 2-((5-hydroxy-2-(4-methoxyphenyl)-8-(3-methylbut-2-en-1-yl)-4-oxo-7-(prop-2-yn-1-yloxy)-4H-chromen-3-yl)oxy)acetate (3a)

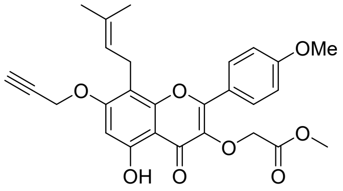

3a

*General synthesis procedure for **3a***. Intermediate **1a** (100 mg, 0.246 mmol), K2CO3 (51 mg, 0.369 mmol) were sequentially added to dry acetone (5 mL) and stirred at room temperature for ten minutes, then methyl 2-bromoacetate (28 l, 0.295 mmol) was added and the reaction was warmed up to 55 °C refluxed and monitored by TLC, and was stopped after about 5 hours. Reaction. At the end of the reaction, ice water was added to quench the stirring, filtration, the filter cake was washed with water for three times and then dried. The crude product was purified by PTLC (DCM : PE : MeOH = 20 : 15 : 1) to give Intermediate **3a** (yellow solid, 103 mg, 87.8%).

Intermediate **3a**. ^{1}H NMR (400 MHz, CHCl3-*d*) δ: 12.59 (s, 1H), 8.20 (s, 2H), 7.04 (s, 2H), 6.52 (s, 1H), 5.22 (s, 1H), 4.81 (s, 4H), 3.92 (s, 3H), 3.76 (s, 3H), 3.54 (s, 2H), 2.58 (s, 1H), 1.82 (s, 3H), 1.71 (s, 3H). ^{13}C NMR (101 MHz, $CDCl_3$): δ: 178.46, 169.34, 161.78, 160.56, 160.03, 156.00, 153.42, 136.54, 132.26, 130.67, 122.95, 121.96, 113.97, 108.51, 105.90, 96.05, 77.62, 76.26, 68.38, 56.49, 55.45, 52.02, 25.80, 21.77, 18.06.

1.3.1.2 methyl 4-((5-hydroxy-2-(4-methoxyphenyl)-8-(3-methylbut-2-en-1-yl)-4-oxo-7-(prop-2-yn-1-yloxy)-4H-chromen-3-yl)oxy)butanoate (4a)

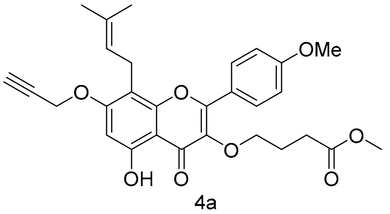

4a

*General synthesis procedure for **4a***. Intermediate **1a** (100 mg, 0.246 mmol), K2CO3 (69 mg, 0.5 mmol) were sequentially added to dry acetone (5 mL) and after ten minutes of stirring at room temperature, 4-bromobutyric acid methyl ester (55 L, 0.434 mmol) was added, the temperature was raised to 55 °C refluxed, and the reaction was monitored by TLC, and was stopped after about 8 hours. . Add ice water to quench the stirring a little, filtration, the filter cake was washed with water three times, dry. The crude product was subjected to PTLC (DCM : PE : MeOH = 20 : 15 : 1) to give intermediate **4a** (yellow solid, 89 mg, 71.4%).

Intermediate **4a**. ^{1}H NMR (500 MHz, Chloroform-*d*) δ: 12.75 (s, 1H), 8.07 (d, 2H), 7.04 (d, 2H), 6.51 (s, 1H), 5.22 (t, 1H), 4.80 (d, 2H), 4.04 (t, J = 6.2 Hz, 2H), 3.92 (s, 3H), 3.68 (s, 3H), 3.53 (d, J = 6.9 Hz, 2H), 2.58 (t, J = 2.4 Hz, 1H), 2.53 (t, J = 7.5 Hz, 2H), 2.09 – 2.04 (m, 2H), 1.80 (s, 3H), 1.71 (s, 3H). ^{13}C NMR (126 MHz, $CDCl_3$) δ: 179.16, 173.65, 161.65, 160.47, 160.16, 156.40, 153.56, 137.53, 132.09, 130.31, 123.17, 122.08, 114.00, 108.46, 106.15, 95.95, 77.69, 76.17, 71.58, 56.50, 55.43, 51.55, 30.48, 25.75, 25.38, 21.76, 18.01.

1.3.1.3 methyl 5-((5-hydroxy-2-(4-methoxyphenyl)-8-(3-methylbut-2-en-1-yl)-4-oxo-7-(prop-2-yn-1-yloxy)-4H-chromen-3-yl)oxy)pentanoate (5a)

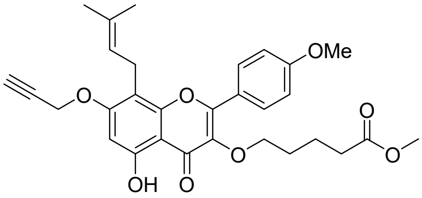

5a

1.3.1.4 methyl 6-((5-hydroxy-2-(4-methoxyphenyl)-8-(3-methylbut-2-en-1-yl)-4-oxo-7-(prop-2-yn-1-yloxy)-4H-chromen-3-yl)oxy)hexanoate (6a)

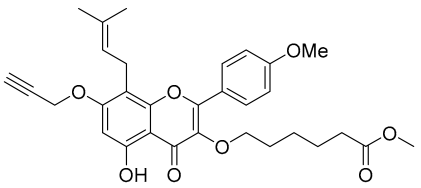

6a

1.3.1.5 methyl 7-((5-hydroxy-2-(4-methoxyphenyl)-8-(3-methylbut-2-en-1-yl)-4-oxo-7-(prop-2-yn-1-yloxy)-4H-chromen-3-yl)oxy)heptanoate (7a)

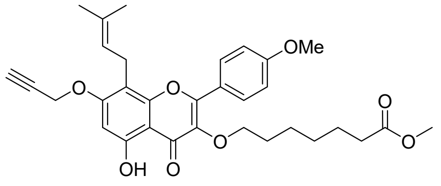

7a

*General synthesis procedure for **5a/6a/7a***. Intermediate **1a** (1 equiv, 0.295 mmol), K_2CO_3 (3.5 equiv), KI (0.3 equiv) were sequentially added to dry acetonitrile (4 mL), stirred at room temperature for ten minutes, then methyl 5-bromopentanoate (6-bromohexanoic acid methyl ester/7-bromoheptanoic acid methyl ester) was added, and the reaction was refluxed at 55 °C for about 12 h. The reaction was monitored by TLC. The reaction was monitored by TLC. The reaction was quenched by the addition of crushed ice, and the reaction solution was extracted with DCM (5 mL×3), the organic phase was washed with saturated NaCl, dried with anhydrous Na_2SO_4, filtered, and concentrated under reduced pressure to obtain intermediates **5a/6a/7a** after purification by PTLC (DCM : PE : MeOH = 15 : 20 : 1).

Intermediate **5a** (yellow solid, 63.8%). ^{1}H NMR (500 MHz, Chloroform-*d*) δ: 12.78 (s, 1H), 8.09 (d, 2H), 7.04 (d, *J* = 7.4 Hz, 2H), 6.51 (s, 1H), 5.22 (t, 1H), 4.80 (d, *J* = 2.4 Hz, 2H), 4.00 (t, 2H), 3.92 (s, 3H), 3.68 (s, 3H), 3.53 (d, *J* = 6.9 Hz, 2H), 2.58 (t, *J* = 2.4 Hz, 1H), 2.37 – 2.33 (m, 2H), 1.80 (s, 3H), 1.78 (q, *J* = 3.2 Hz, 4H), 1.71 (s, 3H). ^{13}C NMR (126 MHz, $CDCl_3$) δ: 179.26, 173.88, 161.62, 160.43, 160.15, 156.39, 153.55, 137.66, 132.11, 130.34, 123.20, 122.07, 113.96, 108.42, 106.14, 95.91, 77.69, 76.19, 72.13, 56.48, 55.43, 51.49, 33.58, 29.42, 25.78, 21.76, 21.44, 18.03.

Intermediate **6a** (yellow solid, 56.4%). ^{1}H NMR (500 MHz, Chloroform-*d*) δ: 12.79 (s, 1H), 8.10 (d, 2H), 7.04 (d, 2H), 6.51 (s, 1H), 5.22 (t, 1H), 4.80 (d, *J* = 2.4 Hz, 2H), 4.00 (t, *J* = 6.6 Hz, 2H), 3.93 (s, 3H), 3.92 (s, 1H), 3.68 (s, 3H), 3.53 (d, *J* = 7.0 Hz, 2H), 2.58 (t, *J* = 2.4 Hz, 1H), 2.31 (t, *J* = 7.5 Hz, 2H), 1.80 (s, 3H), 1.79 – 1.73 (m, 2H), 1.71 (d, *J* = 1.4 Hz, 3H), 1.68 – 1.62 (m, 2H), 1.48 – 1.42 (m, 2H). ^{13}C NMR (126 MHz, $CDCl_3$) δ: 179.30, 174.04, 161.61, 160.42, 160.15, 156.34, 153.55, 137.72, 132.11, 130.36, 123.27, 122.08, 113.93, 108.40, 106.15, 95.90, 77.70, 76.18, 72.58, 56.48, 55.43, 51.48, 33.99, 29.76, 25.78, 25.53, 24.69, 21.76, 18.03.

Intermediate **7a** (yellow solid, 51.9%). ^{1}H NMR (500 MHz, Chloroform-*d*) δ: 12.80 (s, 1H), 8.14 – 8.09 (m, 2H), 7.04 (d, 2H), 6.51 (s, 1H), 5.22 (t, *J* = 5.6, 2.8, 1.3 Hz, 1H), 4.80 (d, *J* = 2.4 Hz, 2H), 4.00 (t, *J* = 6.6 Hz, 2H), 3.92 (s, 3H), 3.68 (s, 3H), 3.53 (d, *J* = 6.9 Hz, 2H), 2.58 (t, *J* = 2.4 Hz, 1H), 2.31 (t, *J* = 7.6 Hz, 2H), 1.81 (s, 3H), 1.77 – 1.72 (m, 2H), 1.71 (s, 3H), 1.66 – 1.60 (m, 2H),

1.46 – 1.40 (m, 2H), 1.36 – 1.31 (m, 2H). ^{13}C NMR (126 MHz, $CDCl_3$) δ: 179.33, 174.18, 161.59, 160.41, 160.16, 156.30, 153.55, 137.76, 132.11, 130.36, 123.30, 122.09, 113.91, 108.39, 106.15, 95.89, 77.70, 76.17, 72.77, 56.48, 55.43, 51.46, 33.99, 29.90, 28.86, 25.78, 25.60, 24.86, 21.77, 18.03.

Intermediates **12a~16a** were synthesized similarly to **3a~7a**, taking **12a** as an example.

1.3.1.6 methyl 7-((5-hydroxy-2-(4-methoxyphenyl)-8-(3-methylbut-2-en-1-yl)-4-oxo-7-(prop-2-yn-1-yloxy)-4H-chromen-3-yl)oxy)heptanoate (12a)

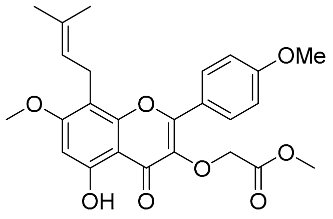

12a

1.3.1.7 methyl 4-((5-hydroxy-7-methoxy-2-(4-methoxyphenyl)-8-(3-methylbut-2-en-1-yl)-4-oxo-4H-chromen-3-yl)oxy)butanoate (13a)

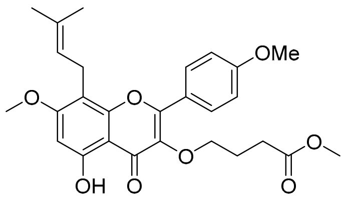

13a

1.3.1.8 methyl 5-((5-hydroxy-7-methoxy-2-(4-methoxyphenyl)-8-(3-methylbut-2-en-1-yl)-4-oxo-4H-chromen-3-yl)oxy)pentanoate (14a)

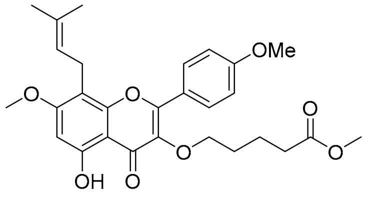

14a

1.3.1.9 methyl 6-((5-hydroxy-7-methoxy-2-(4-methoxyphenyl)-8-(3-methylbut-2-en-1-yl)-4-oxo-4H-chromen-3-yl)oxy)hexanoate (15a)

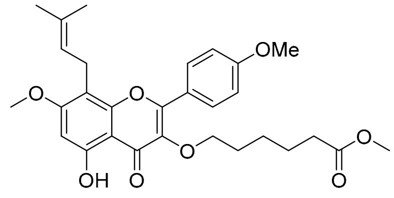

15a

1.3.1.10 methyl 7-((5-hydroxy-2-(4-methoxyphenyl)-8-(3-methylbut-2-en-1-yl)-4-oxo-7-(prop-2-yn-1-yloxy)-4H-chromen-3-yl)oxy)heptanoate (16a)

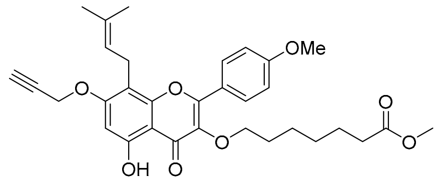

16a

*General synthesis procedure for **12a**.* Intermediate **2a** (104 mg, 0.272 mmol), K_2CO_3 (76 mg, 0.55 mmol) were sequentially added to dry acetone (4 mL), stirred at room temperature for ten minutes, then methyl 2-bromoacetate (31 L, 0.326 mmol) was added, and the reaction was heated up to 55 °C and refluxed for about 5 h. The end of the reaction was monitored by TLC. The reaction was quenched by the addition of ice water, stirred at room temperature, filtered, and the filter cake was washed with water three times and dried. The crude product was purified by PTLC (DCM: PE: MeOH = 20 : 15 : 1) to give **12a** intermediate (yellow solid, 102 mg, 82.5%).

Intermediate **12a**. ^{1}H NMR (500 MHz, $CHCl_3$ -*d*) δ: 8.20 (d, J = 9.1 Hz, 2H), 7.04 (d, J = 9.1 Hz, 2H), 6.42 (s, 1H), 5.20 (t, J = 6.7 Hz, 1H), 4.81 (s, 2H), 3.92 (d, J = 2.3 Hz, 6H), 3.76 (s, 3H), 3.52 (d, 2H), 1.80 (s, 3H), 1.71 (s, 3H). ^{13}C NMR (126 MHz, $CDCl_3$) δ: 178.46, 169.34, 162.78, 161.73, 160.30, 155.82, 153.33, 136.47, 132.04, 130.64, 123.08, 122.23, 113.94, 107.80, 105.33, 94.92, 68.42, 56.08, 55.42, 51.95, 25.74, 21.65, 17.94.

Intermediate **13a** (yellow solid, 91 mg, 71.4%). ^{1}H NMR (500 MHz, Chloroform-*d*): δ: 12.76 (s, 1H), 8.08 (d, J = 7.1 Hz, 2H), 7.03 (d, J = 7.1 Hz, 2H), 6.42 (s, 1H), 5.21 (t, 1H), 4.04 (t, J = 6.2 Hz, 2H), 3.92 (d, J = 1.6 Hz, 6H), 3.68 (s, 3H), 3.50 (d, J = 6.9 Hz, 2H), 2.53 (t, J = 7.4 Hz, 2H), 2.06 (p, J = 13.8, 7.5, 6.2 Hz, 2H), 1.79 (s, 3H), 1.71 (s, 3H). ^{13}C NMR (126 MHz, $CDCl_3$) δ: 179.15, 173.67, 162.65, 161.58, 160.40, 156.24, 153.44, 137.42, 131.93, 130.29, 123.27, 122.31, 113.97, 107.70, 105.54, 94.79, 71.57, 56.05, 55.42, 51.54, 30.49, 25.74, 25.39, 21.64, 17.93.

Intermediate **14a** (yellow solid, 70 mg, 67.4%). ^{1}H NMR (500 MHz, Chloroform-d) δ: 12.79 (s, 1H), 8.09 (d, 2H), 7.03 (d, 2H), 6.42 (s, 1H), 5.21 (t, J = 6.4, 6.0, 3.8 Hz, 1H), 4.01 (t, 2H), 3.92 (d, J = 1.5 Hz, 6H), 3.68 (s, 3H), 3.51 (d, J = 6.9 Hz, 2H), 2.37 – 2.34 (m, 2H), 1.79 (s, 4H), 1.78 (s, 3H), 1.71 (s, 3H). 13C NMR (126 MHz, $CDCl_3$) δ: 179.25, 173.88, 162.63, 161.56, 160.40, 156.22, 153.44, 137.57, 131.94, 130.32, 123.32, 122.32, 113.93, 107.68, 105.55, 94.77, 72.12, 56.06, 55.42, 51.47, 33.59, 29.43, 25.76, 21.64, 21.45, 17.94.

Intermediate **15a** (yellow solid, 68 mg, 50.9%). ^{1}H NMR (500 MHz, Chloroform-*d*) δ : 8.10 (d, 2H), 7.03 (d, 2H), 6.42 (s, 1H), 5.21 (t, J = 6.6, 5.4, 1.3 Hz, 1H), 4.00 (t, J = 6.6 Hz, 2H), 3.92 (d, J = 3.2 Hz, 6H), 3.68 (s, 3H), 3.51 (d, J = 6.9 Hz, 2H), 2.31 (t, J = 7.5 Hz, 2H), 1.80 (s, 3H),

1.78 – 1.73 (m, 2H), 1.71 (s, 3H), 1.68 – 1.62 (m, 2H), 1.48 – 1.42 (m, 2H). ^{13}C NMR (126 MHz, $CDCl_3$) *δ*: 179.30, 174.04, 162.62, 161.55, 160.40, 156.17, 153.44, 137.62, 131.93, 130.33, 123.38, 122.32, 113.89, 107.67, 105.55, 94.75, 72.58, 56.06, 55.41, 51.46, 33.99, 29.77, 25.75, 25.53, 24.69, 21.65, 17.94.

Intermediate **16a** (yellow solid, 60 mg, 46.2%). ^{1}H NMR (500 MHz, Chloroform-*d*) *δ*: 12.80 (s, 1H), 8.11 (d, 2H), 7.03 (d, 2H), 6.42 (s, 1H), 5.21 (t, *J* = 8.2, 6.5, 2.7, 1.3 Hz, 1H), 4.00 (t, *J* = 6.6 Hz, 2H), 3.92 (d, *J* = 2.5 Hz, 6H), 3.68 (s, 3H), 3.50 (dd, *J* = 6.9, 4.2 Hz, 2H), 2.31 (t, *J* = 7.5 Hz, 2H), 1.80 (s, 1H), 1.77 – 1.73 (m, 2H), 1.71 (s, 3H), 1.66 – 1.60 (m, 2H), 1.46 – 1.39 (m, 2H), 1.36 – 1.31 (m, 2H). ^{13}C NMR (126 MHz, $CDCl_3$) *δ*: 179.32, 174.17, 162.61, 161.53, 160.40, 156.14, 153.44, 137.66, 131.93, 130.34, 123.42, 122.33, 113.88, 107.65, 105.56, 94.74, 72.76, 56.05, 55.41, 51.44, 33.99, 29.91, 28.86, 25.75, 25.60, 24.86, 21.65, 17.94.

1.3.2 Synthesis of intermediates 3b~7b, 12b~16b

1.3.2.1 2-((5-hydroxy-2-(4-methoxyphenyl)-8-(3-methylbut-2-en-1-yl)-4-oxo-7-(prop-2-yn-1-yloxy)-4H-chromen-3-yl)oxy)acetic acid (3b)

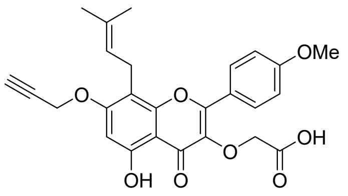

3b

1.3.2.2 4-((5-hydroxy-2-(4-methoxyphenyl)-8-(3-methylbut-2-en-1-yl)-4-oxo-7-(prop-2-yn-1-yloxy)-4H-chromen-3-yl)oxy)butanoic acid (4b)

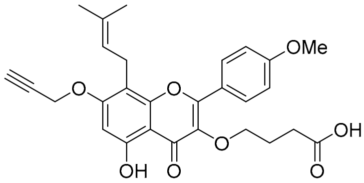

4b

1.3.2.3 5-((5-hydroxy-2-(4-methoxyphenyl)-8-(3-methylbut-2-en-1-yl)-4-oxo-7-(prop-2-yn-1-yloxy)-4H-chromen-3-yl)oxy)pentanoic acid (5b)

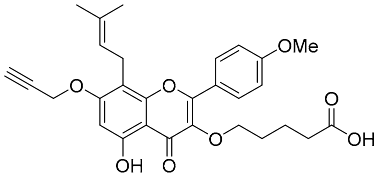

5b

1.3.2.4 6-((5-hydroxy-2-(4-methoxyphenyl)-8-(3-methylbut-2-en-1-yl)-4-oxo-7-(prop-2-yn-1-yloxy)-4H-chromen-3-yl)oxy)hexanoic acid (6b)

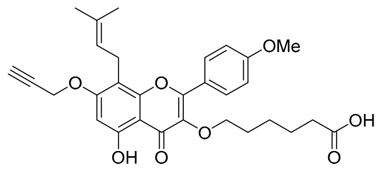

6b

1.3.2.5 7-((5-hydroxy-2-(4-methoxyphenyl)-8-(3-methylbut-2-en-1-yl)-4-oxo-7-(prop-2-yn-1-yloxy)-4H-chromen-3-yl)oxy)heptanoic acid (7b)

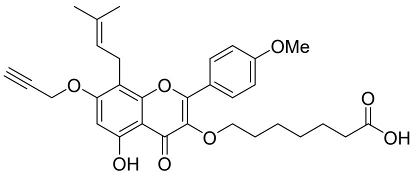

7b

1.3.2.6 2-((5-hydroxy-7-methoxy-2-(4-methoxyphenyl)-8-(3-methylbut-2-en-1-yl)-4-oxo-4H-chromen-3-yl)oxy)acetic acid (12b)

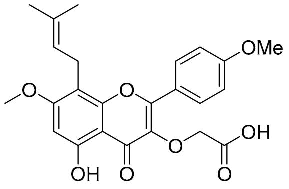

12b

1.3.2.7 4-((5-hydroxy-7-methoxy-2-(4-methoxyphenyl)-8-(3-methylbut-2-en-1-yl)-4-oxo-4H-chromen-3-yl)oxy)butanoic acid (13b)

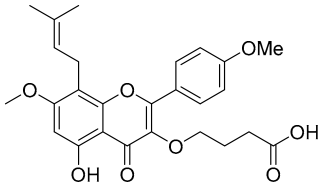

13b

1.3.2.8 5-((5-hydroxy-7-methoxy-2-(4-methoxyphenyl)-8-(3-methylbut-2-en-1-yl)-4-oxo-4H-chromen-3-yl)oxy)pentanoic acid (14b)

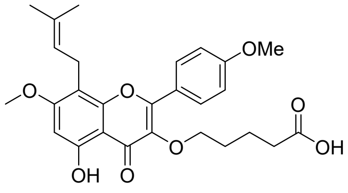

14b

1.3.2.9 6-((5-hydroxy-7-methoxy-2-(4-methoxyphenyl)-8-(3-methylbut-2-en-1-yl)-4-oxo-4H-chromen-3-yl)oxy)hexanoic acid (15b)

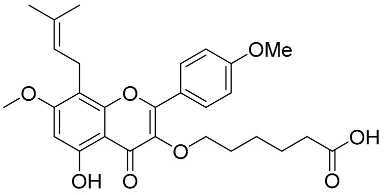

15b

1.3.2.10 7-((5-hydroxy-7-methoxy-2-(4-methoxyphenyl)-8-(3-methylbut-2-en-1-yl)-4-oxo-4H-chromen-3-yl)oxy)heptanoic acid (16b)

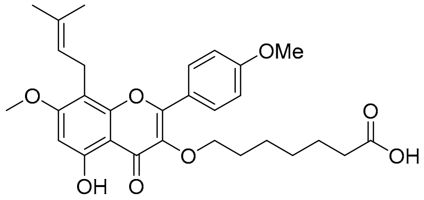

16b

*General synthesis procedure for **3b***. Intermediate **3a** (90 mg, 0.188 mmol), LiOH (18 mg, 0.752 mmol) was dissolved in a mixture of THF/H_2O (V:V=1:1) (2 mL), and the reaction was warmed up to 40°C and monitored by TLC, and stopped after about 4 hours. The PH was adjusted to about 2 with 1 M HCl solution, filtered, the filter cake was washed with water 3 times and dried to give intermediate **3b** (light yellow solid, 83 mg, yield 95.0%), which was used directly in the next step without purification.

Intermediate **3b**. ^{1}H NMR (400 MHz, Chloroform-*d*) δ: 8.08 (d, 2H), 7.09 (d, 2H), 6.60 (s, 1H), 5.20 (t, J = 7.7 Hz, 1H), 4.83 (s, 2H), 4.40 (s, 2H), 3.95 (s, 3H), 3.55 (d, J = 7.0 Hz, 2H), 2.61 (s, 1H), 1.81 (s, 3H), 1.71 (s, 3H).^{13}C NMR (101 MHz, $CDCl_3$) δ: 178.92, 162.64, 161.64, 159.68, 157.80, 153.62, 138.24, 132.64, 130.47, 121.61, 121.52, 114.66, 109.27, 104.96, 96.85, 77.34, 77.23, 76.60, 72.45, 56.66, 55.60, 25.76, 21.75, 18.06.

Intermediates **4b**~**7b**, **12b**~**16b** were synthesized similarly to **3b**.

Intermediate **4b** (yellow solid, 94.7%). ^{1}H NMR (500 MHz, Chloroform-*d*) δ : 12.67 (s, 1H), 8.07 (d, 2H), 7.04 (d, 2H), 6.52 (s, 1H), 5.21 (t, J = 7.0, 5.8, 1.4 Hz, 1H), 4.80 (d, J = 2.4 Hz, 2H), 4.04 (t, J = 6.0 Hz, 2H), 3.92 (s, 3H), 3.53 (d, J = 6.9 Hz, 2H), 2.62 (t, J = 7.2 Hz, 2H), 2.58 (t, J = 2.4 Hz, 1H), 2.11 – 2.05 (m, 2H), 1.80 (s, 3H), 1.71 (s, 3H).

Intermediate **5b** (yellow solid, 92.5%). ^{1}H NMR (500 MHz, Chloroform-*d*) δ: 8.09 (d, 2H), 7.04 (d, 2H), 6.51 (s, 1H), 5.24 – 5.19 (m, 1H), 4.80 (d, J = 2.4 Hz, 2H), 4.01 (t, 2H), 3.91 (s, 3H), 3.53 (t, 2H), 2.58 (t, J = 2.4 Hz, 1H), 2.42 (t, 2H), 1.84 – 1.78 (m, 7H), 1.71 (s, 3H). ^{13}C NMR (126 MHz, $CDCl_3$) δ: 178.14, 161.67, 160.47, 160.15, 156.46, 153.57, 137.65, 132.10, 130.33, 123.17, 122.08, 113.99, 108.46, 106.16, 95.96, 77.69, 76.16, 72.04, 56.50, 55.42, 33.32, 29.29, 25.75, 21.76,

21.27, 18.01.

Intermediate **6b** (yellow solid, 84.0%). ^{1}H NMR (500 MHz, Chloroform-*d*) δ: 12.77 (s, 1H), 8.09 (d, 2H), 7.03 (d, 2H), 6.51 (s, 1H), 5.22 (t, J = 6.9, 1.4 Hz, 1H), 4.80 (d, J = 2.4 Hz, 2H), 4.00 (t, J = 6.5 Hz, 2H), 3.92 (s, 3H), 3.53 (d, J = 6.9 Hz, 2H), 2.58 (t, J = 2.4 Hz, 1H), 2.36 (t, J = 7.5 Hz, 2H), 1.80 (s, 3H), 1.79 – 1.74 (m, 2H), 1.71 (s, 3H), 1.69 – 1.63 (m, 2H), 1.54 – 1.45 (m, 2H).

Intermediate **7b** (yellow solid, 94.7%). ^{1}H NMR (500 MHz, Chloroform-*d*) δ: 8.10 (d, 2H), 7.04 (d, 2H), 6.51 (s, 1H), 5.22 (t, 1H), 4.80 (d, J = 2.4 Hz, 2H), 4.00 (t, J = 6.6 Hz, 2H), 3.92 (s, 3H), 3.53 (t, 2H), 2.58 (t, J = 2.4 Hz, 1H), 2.35 (t, J = 7.5 Hz, 2H), 1.81 (s, 3H), 1.77 – 1.73 (m, 2H), 1.71 (s, 3H), 1.67 – 1.61 (m, 2H), 1.47 – 1.41 (m, 2H), 1.39 – 1.33 (m, 2H). ^{13}C NMR (126 MHz, $CDCl_3$) δ: 179.33, 178.38, 161.61, 160.43, 160.16, 156.34, 153.57, 137.77, 132.08, 130.37, 123.31, 122.10, 113.92, 108.42, 106.17, 95.92, 77.71, 76.15, 72.74, 56.49, 55.42, 33.67, 29.86, 28.74, 25.75, 25.57, 24.59, 21.76, 18.01.

Intermediate **12b** (yellow solid, 94.7%). ^{1}H NMR (500 MHz, Chloroform-*d*) δ: 11.69 (s, 1H), 8.08 (d, J = 8.3 Hz, 2H), 7.08 (d, J = 8.4 Hz, 2H), 6.49 (s, 1H), 5.17 (t, J = 1.4 Hz, 1H), 4.39 (s, 2H), 3.95 (s, 6H), 3.53 (d, J = 6.8 Hz, 2H), 1.80 (s, 3H), 1.71 (s, 3H). ^{13}C NMR (126 MHz, $CDCl_3$) δ: 178.86, 169.96, 163.89, 162.59, 159.95, 157.64, 153.51, 138.18, 132.44, 130.43, 121.71, 114.63, 108.61, 104.36, 95.72, 72.54, 56.26, 55.57, 25.72, 21.63, 17.96.

Intermediate **13b** (yellow solid, 91.6%). ^{1}H NMR (500 MHz, Chloroform-*d*) δ: 8.07 (d, 2H), 7.03 (d, 2H), 6.43 (s, 1H), 5.20 (t, J = 6.9, 2.8, 1.5 Hz, 1H), 4.03 (t, J = 6.0 Hz, 2H), 3.92 (d, J = 2.3 Hz, 6H), 3.51 (d, J = 6.9 Hz, 2H), 2.62 (t, J = 7.2 Hz, 2H), 2.11 – 2.05 (m, 2H), 1.79 (s, 3H), 1.71 (s, 3H).

Intermediate **14b** (yellow solid, 88.5%). ^{1}H NMR (500 MHz, Chloroform-*d*) δ: 8.09 (d, 2H), 7.03 (d, 2H), 6.42 (s, 1H), 5.21 (t, J = 8.3, 5.5, 2.7, 1.3 Hz, 1H), 4.01 (t, 2H), 3.92 (d, J = 2.9 Hz, 6H), 3.51 (d, 2H), 2.42 (t, 2H), 1.83 – 1.80 (m, 4H), 1.79 (s, 3H), 1.71 (s, 3H). ^{13}C NMR (126 MHz, $CDCl_3$) δ: 179.23, 178.49, 162.66, 161.60, 160.39, 156.30, 153.46, 137.54, 131.93, 130.31, 123.28, 122.31, 113.95, 107.71, 105.55, 94.80, 72.03, 56.06, 55.41, 33.38, 29.30, 25.74, 21.64, 21.27, 17.93.

Intermediate **15b** (yellow solid, 92.5%). ^{1}H NMR (500 MHz, Chloroform-*d*) δ: 8.09 (d, 2H), 7.03 (d, 2H), 6.42 (s, 1H), 5.21 (t, J = 7.1, 5.6, 1.5 Hz, 1H), 4.01 (t, J = 6.5 Hz, 2H), 3.92 (s, 6H), 3.51 (d, J = 7.0 Hz, 2H), 2.36 (t, J = 7.5 Hz, 2H), 1.79 (s, 3H), 1.78 – 1.73 (m, 2H), 1.71 (d, J = 1.4 Hz, 3H), 1.68 – 1.63 (m, 2H), 1.52 – 1.44 (m, 2H). ^{13}C NMR (126 MHz, $CDCl_3$) δ: 179.29, 178.29, 162.64, 161.56, 160.39, 156.22, 153.45, 137.61, 131.92, 130.34, 123.37, 122.33, 113.90, 107.69, 105.56, 94.77, 72.52, 56.05, 55.42, 33.71, 29.73, 25.74, 25.46, 24.42, 21.64, 17.93.

Intermediate **16b** (yellow solid, 94.1%).^{1}H NMR (500 MHz, Chloroform-*d*) δ: 8.11 (d, 2H), 7.03 (d, 2H), 6.42 (s, 1H), 5.21 (t, J = 5.5, 1.7 Hz, 1H), 4.00 (t, J = 6.6 Hz, 2H), 3.92 (s, 6H), 3.51

(d, 2H), 2.35 (t, J = 7.5 Hz, 2H), 1.80 (s, 3H), 1.78 – 1.73 (m, 2H), 1.71 (s, 3H), 1.68 – 1.61 (m, 2H), 1.47 – 1.40 (m, 2H), 1.39 – 1.33 (m, 2H). ^{13}C NMR (126 MHz, $CDCl_3$) δ: 179.32, 178.42, 162.62, 161.54, 160.40, 156.17, 153.45, 137.66, 131.91, 130.35, 123.42, 122.34, 113.88, 107.67, 105.57, 94.76, 72.74, 56.05, 55.41, 33.70, 29.87, 28.75, 25.74, 25.58, 24.59, 21.65, 17.93.

Supplementary Scheme 4. Synthesis of end products 3-14

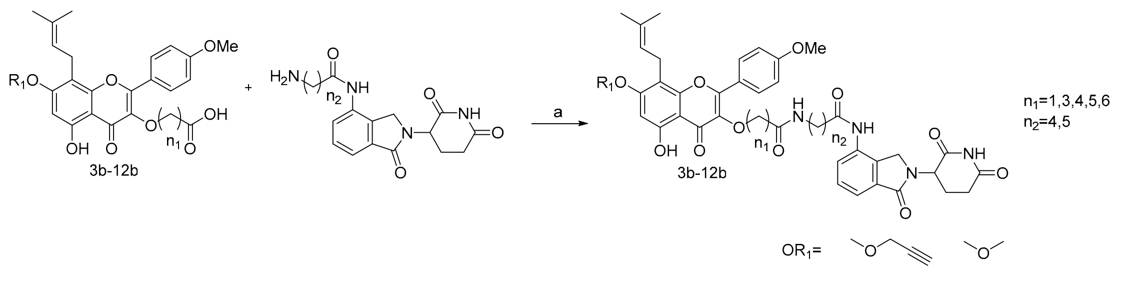

$^{\alpha}$Reagents and conditions: (a) HATU/PyBOP, DIPEA, DMF, RT

1.3.3 Synthesis of compounds 3 to 7

1.3.3.1 N-(2-(2,6-dioxopiperidin-3-yl)-1-oxoisoindolin-4-yl)-6-(2-((5-hydroxy-2-(4-methoxyphenyl)-8-(3-methylbut-2-en-1-yl)-4-oxo-7-(prop-2-yn-1-yloxy)-4H-chromen-3-yl)oxy)acetamido)hexanamide (compound 3)

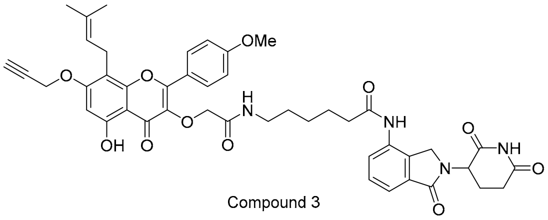

Compound 3

1.3.3.2 N-(2-(2,6-dioxopiperidin-3-yl)-1-oxoisoindolin-4-yl)-6-(4-((5-hydroxy-2-(4-methoxyphenyl)-8-(3-methylbut-2-en-1-yl)-4-oxo-7-(prop-2-yn-1-yloxy)-4H-chromen-3-yl)oxy)butanamido)hexanamide (compound 4)

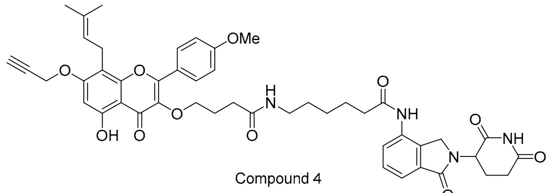

Compound 4

1.3.3.3 N-(2-(2,6-dioxopiperidin-3-yl)-1-oxoisoindolin-4-yl)-6-(5-((5-hydroxy-2-(4-methoxyphenyl)-8-(3-methylbut-2-en-1-yl)-4-oxo-7-(prop-2-yn-1-yloxy)-4H-chromen-3-yl)oxy)pentanamido)hexanamide (compound 5)

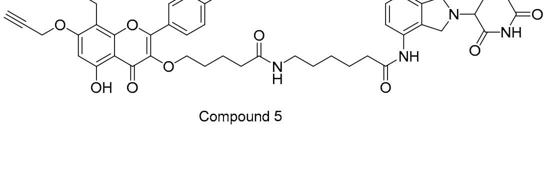

Compound 5

1.3.3.4 N-(2-(2,6-dioxopiperidin-3-yl)-1-oxoisoindolin-4-yl)-6-(6-((5-hydroxy-2-(4-methoxyphenyl)-8-(3-methylbut-2-en-1-yl)-4-oxo-7-(prop-2-yn-1-yloxy)-4H-chromen-3-yl)oxy)hexanamido)hexanamide (compound 6)

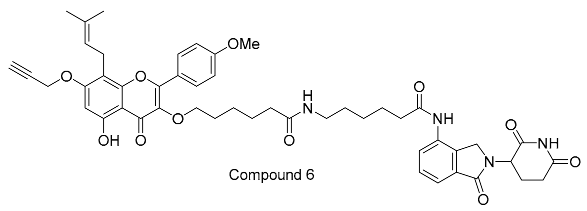

Compound 6

1.3.3.5 N-(6-((2-(2,6-dioxopiperidin-3-yl)-1-oxoisoindolin-4-yl)amino)-6-oxohexyl)-7-((5-hydroxy-2-(4-methoxyphenyl)-8-(3-methylbut-2-en-1-yl)-4-oxo-7-(prop-2-yn-1-yloxy)-4H-chromen-3-yl)oxy)heptanamide (compound 7)

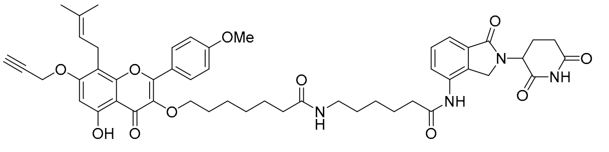

Compound 7

Intermediate **3b/4b** (1.1 equiv), DIPEA (3 equiv) were sequentially dissolved in dry DMF solution and stirred for five minutes at room temperature , HATU (1.2~1.5 equiv) was added and monitored by TLC for about half an hour, then intermediate **s3** (1equiv) was added, and the reaction was stopped for about 12 hours at room temperature. The reaction was quenched by addition of crushed ice, EA extraction (5 mL x 3), the organic phase was washed three times sequentially with saturated saturated NH4Cl and saturated NaCl, dried over anhydrous Na_2SO_4, filtered, and the solvent was removed to remove **the compound 3/4** after purification by PTLC (DCM : MeOH = 15 : 1).

Compound 3 (light yellow solid, 53.0%). ^{1}H NMR (400 MHz, Chloroform-*d*) δ: 12.44 (s, 1H), 8.74 (s, 1H), 8.37 (d, J = 32.0 Hz, 2H), 7.95 (s, 2H), 7.69 (s, 2H), 7.44 (s, 1H), 7.03 (s, 2H), 6.51 (s, 1H), 5.14 (t, J = 17.0 Hz, 2H), 4.79 (s, 2H), 4.42 (s, 2H), 4.25 (s, 2H), 3.92 (s, 3H), 3.50 (t, J = 5.5 Hz, 2H), 3.35 (s, 2H), 2.87 – 2.68 (m, 2H), 2.60 (s, 1H), 2.49 – 2.23 (m, 4H), 1.84 – 1.75 (m, 5H), 1.70 (s, 3H), 1.67 – 1.58 (m, 2H), 1.52 – 1.39 (m, 2H). ^{13}C NMR (101 MHz, $CDCl_3$) δ: 178.77, 171.74, 171.31, 169.93, 169.03, 162.24, 161.03, 159.72, 157.00, 153.47, 138.00, 134.32, 132.96, 132.62, 132.47, 130.18, 128.99, 126.19, 122.00, 121.63, 120.84, 114.54, 109.02, 105.57, 96.33, 77.24, 76.48, 72.74, 56.59, 55.59, 51.85, 46.76, 38.86, 36.75, 31.50, 28.81, 26.19, 25.79, 25.17, 23.27, 21.71, 18.06. HRMS: calculated for $C_{45}H_{46}N_4O_{11}$ [M + Na]$^+$, 841.3056.; found, 841.3059.

Compound 4 (yellow solid, 46.1%). ^{1}H NMR (500 MHz, Chloroform-*d*) δ: 12.67 (s, 1H), 8.06 (d, J = 8.5 Hz, 2H), 7.76 (d, J = 7.8 Hz, 1H), 7.65 (d, J = 7.4 Hz, 1H), 7.43 (t, J = 7.7 Hz, 1H), 7.26 (s, 1H), 7.02 (d, J = 8.5 Hz, 2H), 6.46 (s, 1H), 5.32 (s, 1H), 5.18 (t, J = 5.7, 4.1, 1.8 Hz, 1H),

5.10 (s, 1H), 4.75 (d, *J* = 2.4 Hz, 2H), 4.42 (s, 2H), 3.90 (s, 3H), 3.84 (s, 2H), 3.50 (d, *J* = 6.8 Hz, 2H), 3.24 (s, 2H), 2.83 – 2.67 (m, 2H), 2.59 (t, *J* = 2.3 Hz, 1H), 2.52 (s, 2H), 2.42 (s, 2H), 2.00 (s, 2H), 1.79 (s, 3H), 1.74 (s, 2H), 1.69 (s, 3H), 1.54 (s, 2H), 1.38 (s, 2H). ^{13}C NMR (126 MHz, $CDCl_3$) *δ*: 179.29, 173.62, 172.10, 171.57, 170.14, 169.12, 161.95, 160.76, 159.74, 157.19, 153.54, 137.23, 134.26, 133.20, 132.57, 132.38, 130.22, 128.98, 126.24, 122.74, 121.79, 120.63, 114.24, 108.85, 105.89, 96.10, 77.54, 76.41, 70.99, 56.53, 55.51, 53.45, 46.86, 39.40, 36.62, 32.96, 31.51, 28.96, 26.61, 26.19, 25.78, 25.17, 23.24, 21.73, 18.05. HRMS: calculated for $C_{47}H_{50}N_4O_{11}$ $[M + Na]^+$, 869.3368.; found, 869.3368.

Intermediates **5b/6b/7b** (1.1 equiv), DIPEA (5 equiv), PyBOP (3 equiv) were sequentially dissolved in dry DMF solution, stirred at room temperature, and the reaction was monitored by TLC for about half an hour, then intermediate **s3** (1 equiv) was added, and the reaction was stopped by stirring at room temperature for about 12 hours. The reaction was quenched by addition of crushed ice, extracted by EA (5 mL x 3), the organic phase was washed sequentially with saturated NH4Cl and saturated NaCl, dried with anhydrous Na_2SO_4, filtered, concentrated under reduced pressure and purified by PTLC (DCM : MeOH= 15 : 1) to give the **compound 5/6/7**.

Compound 5 (yellow solid, 50.38%). ^{1}H NMR (500 MHz, Chloroform-*d*) *δ* ： 12.71 (s, 1H), 8.91 (s, 1H), 8.66 (s, 1H), 8.07 (d, *J* = 8.6 Hz, 2H), 7.75 (d, *J* = 7.8 Hz, 1H), 7.66 (d, *J* = 7.4 Hz, 1H), 7.45 (t, *J* = 7.6 Hz, 1H), 7.02 (d, *J* = 8.6 Hz, 2H), 6.48 (s, 3H), 5.32 (s, 1H), 5.19 (t, 1H), 5.13 (d, 1H), 4.78 (d, *J* = 2.4 Hz, 2H), 4.44 (s, 1H), 3.91 (s, 5H), 3.51 (s, 3H), 3.25 (s, 2H), 2.85 – 2.69 (m, 2H), 2.59 (t, *J* = 2.3 Hz, 1H), 2.45 (s, 2H), 2.30 (s, 2H), 2.16 (s, 1H), 1.84 – 1.72 (m, 9H), 1.70 (s, 3H), 1.58 – 1.51 (m, 2H), 1.44 – 1.37 (m, 2H). ^{13}C NMR (126 MHz, $CDCl_3$) *δ* : 179.35, 173.75, 171.99, 171.37, 169.98, 169.07, 161.82, 160.65, 159.88, 156.82, 153.54, 137.56, 134.29, 133.14, 132.60, 132.29, 130.26, 128.98, 126.23, 122.95, 121.89, 120.71, 114.14, 108.71, 106.02, 96.03, 77.60, 76.32, 71.90, 56.53, 55.49, 51.92, 46.81, 39.12, 36.65, 35.82, 29.72, 26.07, 25.77, 25.06, 23.30, 22.51, 21.75, 18.04. HRMS: calculated for $C_{48}H_{52}N_4O_{11}$ $[M + Na]^+$, 883.3525.; found, 883.3526.

Compound 6 (yellow solid, 57.5%). ^{1}H NMR (500 MHz, Chloroform-*d*) *δ* : 12.73 (s, 1H), 8.08 (d, *J* = 8.5 Hz, 2H), 7.76 (d, *J* = 7.8 Hz, 1H), 7.67 (d, *J* = 7.3 Hz, 1H), 7.45 (t, *J* = 7.4 Hz, 1H), 7.03 (d, *J* = 8.5 Hz, 2H), 6.49 (s, 1H), 6.44 (s, 1H), 5.20 (t, *J* = 6.3, 5.8, 3.7 Hz, 1H), 5.14 (s, 1H), 4.78 (d, *J* = 2.4 Hz, 2H), 4.45 (s, 2H), 3.92 (d, *J* = 5.3 Hz, 5H), 3.53 (s, 1H), 3.51 (s, 3H), 3.26 (s, 2H), 2.87 – 2.68 (m, 3H), 2.59 (t, *J* = 2.3 Hz, 1H), 2.49 – 2.40 (m, 3H), 1.80 (s, 3H), 1.70 (s, 7H), 1.68 – 1.61 (m, 2H), 1.58 – 1.50 (m, 3H), 1.49 – 1.44 (m, 3H). ^{13}C NMR (126 MHz, $CDCl_3$) δ 179.38, 171.35, 169.95, 169.07, 167.03, 164.14, 161.78, 160.62, 159.93, 156.75, 153.54, 137.63, 134.19, 133.13, 132.58, 132.27, 130.31, 128.99, 126.18, 123.03, 121.93, 120.68, 114.09, 108.65,

106.02, 96.02, 77.62, 76.30, 72.43, 56.52, 55.50, 51.92, 50.85, 39.21, 37.58, 36.63, 36.41, 31.93, 29.70, 29.36, 25.77, 25.31, 22.70, 21.76, 18.04. HRMS: calculated for $C_{49}H_{54}N_4O_{11}$ $[M + Na]^+$, 897.3682.; found, 897.3683.

Compound 7 (yellow solid, 48.3%). ^{1}H NMR (500 MHz, Chloroform-*d*) δ: 12.74 (s, 1H), 9.14 (s, 1H), 8.81 (s, 1H), 8.08 (d, 2H), 7.75 (d, *J* = 7.8 Hz, 1H), 7.64 (d, *J* = 7.4 Hz, 1H), 7.43 (t, *J* = 7.7 Hz, 1H), 7.02 (d, 2H), 6.49 (s, 1H), 6.18 (s, 1H), 5.20 (t, *J* = 7.1, 5.3, 1.7 Hz, 1H), 5.10 (d, *J* = 12.3 Hz, 1H), 4.78 (d, *J* = 2.4 Hz, 2H), 4.41 (s, 2H), 3.93 (d, *J* = 6.6 Hz, 2H), 3.90 (s, 3H), 3.51 (t, 2H), 3.21 (s, 2H), 2.82 – 2.67 (m, 2H), 2.59 (t, *J* = 2.4 Hz, 1H), 2.43 (s, 2H), 2.17 – 2.01 (m, 6H), 1.80 (s, 3H), 1.71 (s, 7H), 1.58 (d, *J* = 7.3 Hz, 2H), 1.51 (s, 2H), 1.41 – 1.33 (m, 4H). ^{13}C NMR (126 MHz, $CDCl_3$) δ :179.37, 173.71, 172.09, 171.58, 170.15, 169.14, 161.69, 160.55, 159.98, 156.56, 153.53, 137.69, 134.22, 133.20, 132.54, 132.21, 130.32, 128.99, 126.26, 123.14, 121.98, 120.60, 114.02, 108.57, 106.06, 95.97, 77.67, 76.26, 72.80, 56.51, 55.49, 51.93, 46.87, 39.17, 36.52, 31.49, 29.87, 29.69, 29.14, 28.84, 26.16, 25.76, 25.66, 25.49, 25.02, 23.22, 21.76, 18.03. HRMS: $C_{50}H_{56}N_4O_{11}$ $[M + Na]^+$, 911.3838.; found, 911.3838.

1.3.4 Synthesis of compounds 12 to 16

1.3.4.1N-(2-(2,6-dioxopiperidin-3-yl)-1-oxoisoindolin-4-yl)-6-(2-((5-hydroxy-7-methoxy-2-(4-methoxyphenyl)-8-(3-methylbut-2-en-1-yl)-4-oxo-4H-chromen-3-yl)oxy)acetamido)hexanamide (compound 12)

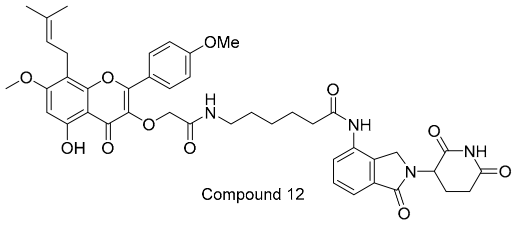

Compound 12

1.3.4.2 N-(2-(2,6-dioxopiperidin-3-yl)-1-oxoisoindolin-4-yl)-6-(4-((5-hydroxy-7-methoxy-2-(4-methoxyphenyl)-8-(3-methylbut-2-en-1-yl)-4-oxo-4H-chromen-3-yl)oxy)butanamido)hexanamide (compound 13)

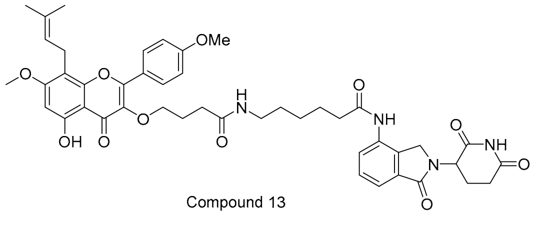

Compound 13

1.3.4.3 N-(2-(2,6-dioxopiperidin-3-yl)-1-oxoisoindolin-4-yl)-6-(5-((5-hydroxy-7-methoxy-2-(4-methoxyphenyl)-8-(3-methylbut-2-en-1-yl)-4-oxo-4H-chromen-3-yl)oxy)pentanamido)hexanamide (compound 14)

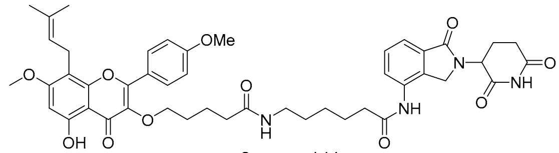

Compound 14

1.3.4.4 N-(2-(2,6-dioxopiperidin-3-yl)-1-oxoisoindolin-4-yl)-6-(6-((5-hydroxy-7-methoxy-2-(4-methoxyphenyl)-8-(3-methylbut-2-en-1-yl)-4-oxo-4H-chromen-3-yl)oxy)hexanamido)hexanamide (compound 15)

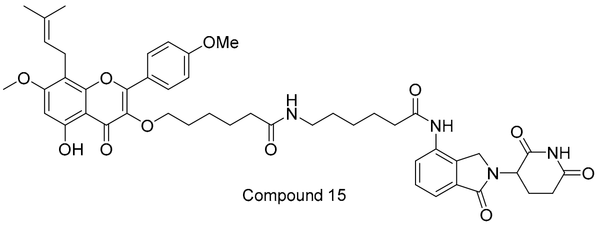

Compound 15

1.3.4.5 N-(6-((2-(2,6-dioxopiperidin-3-yl)-1-oxoisoindolin-4-yl)amino)-6-oxohexyl)-7-((5-hydroxy-7-methoxy-2-(4-methoxyphenyl)-8-(3-methylbut-2-en-1-yl)-4-oxo-4H-chromen-3-yl)oxy)heptanamide (compound 16)

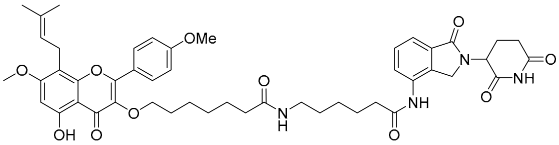

Compound 16

At room temperature, intermediate **12b/13b** (1.1 equiv), DIPEA (3 equiv), and HATU (1.2~1.5 equiv) were sequentially dissolved in dry DMF solution and monitored by TLC, about half an hour, then intermediate **s3** (1 equiv) was added, and the reaction was stopped after continuing at room temperature for about 12 hours. The reaction was quenched by addition of crushed ice, extracted by EA (5 mL x 3), the organic phase was washed sequentially with saturated NH4Cl and saturated NaCl, dried over anhydrous Na2SO4, filtered, concentrated under reduced pressure and **compound 12/13** was purified by PTLC (DCM : MeOH= 15 : 1).

Compound 12 (light yellow solid, 47.0%). ^{1}H NMR (500 MHz, Chloroform-*d*) δ: 12.43 (s, 1H), 8.92 (s, 1H), 8.51 (s, 1H), 8.37 (t, J = 5.8 Hz, 1H), 7.93 (d, 2H), 7.71 (d, J = 7.9 Hz, 1H), 7.65 (d, J = 7.5 Hz, 1H), 7.42 (t, J = 7.7 Hz, 1H), 7.04 – 7.00 (m, 2H), 6.39 (s, 1H), 5.16 (t, J = 6.8, 1.5 Hz, 1H), 5.11 (q, J = 8.3 Hz, 1H), 4.40 (t, 2H), 4.25 (s, 2H), 3.93 – 3.86 (m, 6H), 3.46 (d, J = 6.9 Hz, 2H), 3.33 (q, J = 6.7 Hz, 2H), 2.83 – 2.67 (m, 2H), 2.42 (t, J = 7.5 Hz, 2H), 2.35 – 2.21 (m, 1H), 2.17 – 2.09 (m, 1H), 1.81 – 1.74 (m, 5H), 1.69 (s, 3H), 1.63 (t, J = 7.2 Hz, 2H), 1.49 – 1.40 (m, 2H). ^{13}C NMR (126 MHz, $CDCl_3$) δ : 178.72, 171.85, 171.43, 170.01, 169.07, 169.02, 163.22, 162.14,

160.01, 156.75, 153.34, 137.86, 134.29, 133.06, 132.59, 132.22, 130.11, 128.96, 126.19, 122.15, 121.90, 120.72, 114.47, 108.27, 105.01, 95.17, 72.69, 56.17, 55.53, 51.90, 46.76, 38.85, 36.68, 31.47, 29.69, 26.20, 25.73, 25.16, 23.25, 21.59, 17.94. HRMS: calculated for $C_{43}H_{46}N4O_{11}$ $[M + H]^{+}$, 795.3236.; found, 795.3236.

Compound 13 (yellow solid, 47.9%). MS (ESI) for $C_{45}H_{50}N_4O_{11}$ $[M + Na]^+$ 845.3370. ^{1}H NMR (500 MHz, Chloroform-*d*) δ : 12.73 (s, 1H), 8.98 (s, 1H), 8.62 (s, 1H), 8.07 (d, 2H), 7.73 (d, *J* = 7.9 Hz, 1H), 7.67 (d, *J* = 7.5 Hz, 1H), 7.44 (t, *J* = 7.7 Hz, 1H), 7.07 – 6.99 (m, 3H), 6.36 (s, 1H), 5.18 (t, *J* = 6.1, 5.6, 3.4 Hz, 1H), 5.12 (dd, *J* = 13.2, 5.0 Hz, 1H), 4.43 (t, 2H), 3.90 (s, 3H), 3.88 – 3.83 (m, 5H), 3.49 (t, 2H), 3.27 – 3.21 (m, 2H), 2.84 – 2.69 (m, 2H), 2.50 (t, *J* = 6.5 Hz, 2H), 2.41 (t, *J* = 7.5 Hz, 2H), 2.07 – 1.96 (m, 4H), 1.78 (s, 3H), 1.77 – 1.71 (m, 2H), 1.70 (s, 3H), 1.58 – 1.51 (m, 2H), 1.44 – 1.37 (m, 2H). ^{13}C NMR (126 MHz, $CDCl_3$) δ: 179.30, 171.97, 171.43, 169.99, 169.09, 162.96, 161.87, 160.02, 157.00, 153.45, 137.15, 134.30, 133.13, 132.60, 132.18, 130.19, 128.97, 126.23, 122.90, 122.05, 120.72, 114.19, 108.15, 105.34, 94.94, 70.96, 56.12, 55.47, 51.91, 46.78, 39.18, 36.60, 33.10, 31.50, 29.00, 26.59, 26.11, 25.74, 25.11, 23.26, 21.63, 17.95. HRMS: calculated for C43H46N4O11 $[M + Na]^+$, 845.3368.; found, 845.3370.

Compounds 14 to **16** were synthesized similarly to **compounds 5** to **7**.

Compound 14 (yellow solid, 48.8%). ^{1}H NMR (500 MHz, Chloroform-*d*) δ : 12.72 (s, 1H), 8.96 (s, 1H), 8.69 (s, 1H), 8.06 (d, 2H), 7.74 (d, *J* = 7.9 Hz, 1H), 7.66 (d, *J* = 7.5 Hz, 1H), 7.44 (t, *J* = 7.7 Hz, 1H), 7.02 (d, 2H), 6.38 (s, 1H), 6.35 (s, 1H), 5.18 (t, *J* = 7.0, 5.6, 1.5 Hz, 1H), 5.12 (dd, *J* = 13.2, 5.1 Hz, 1H), 4.43 (t, 2H), 3.92 – 3.90 (m, 4H), 3.89 (s, 3H), 3.49 (t, 2H), 3.24 (q, *J* = 6.6 Hz, 2H), 2.84 – 2.68 (m, 2H), 2.44 (t, *J* = 7.5 Hz, 2H), 2.35 – 2.24 (m, 3H), 2.18 – 2.12 (m, 1H), 1.82 – 1.78 (m, 5H), 1.77 – 1.71 (m, 5H), 1.70 (s, 3H), 1.56 – 1.49 (m, 2H), 1.43 – 1.36 (m, 2H). ^{13}C NMR(126 MHz, $CDCl_3$) δ : 229.34, 179.34, 173.61, 172.09, 171.42, 170.02, 169.08, 162.84, 161.73, 160.14, 156.63, 153.42, 137.46, 134.27, 133.16, 132.58, 132.10, 130.23, 130.23, 128.97, 126.25, 123.07, 122.14, 120.68, 114.09, 107.96, 105.43, 94.87, 71.90, 56.10, 55.47, 51.92, 46.78, 39.01, 36.62, 35.94, 31.50, 29.69, 29.12, 26.06, 25.74, 25.05, 23.26, 22.47, 21.63, 17.94. HRMS: calculated for $C_{46}H_{52}N_4O_{11}$ $[M + Na]^+$, 859.3525.; found, 859.3527.

Compound 15 (yellow solid, 61.8%). ^{1}H NMR (500 MHz, Chloroform-*d*) δ : 12.73 (s, 1H), 8.85 (s, 1H), 8.61 (s, 1H), 8.08 (d, *J* = 8.7 Hz, 2H), 7.75 (d, *J* = 7.9 Hz, 1H), 7.67 (d, *J* = 7.5 Hz, 1H), 7.44 (t, *J* = 7.7 Hz, 1H), 7.03 (d, *J* = 8.7 Hz, 2H), 6.39 (s, 1H), 6.23 (s, 1H), 5.19 (t, *J* = 6.9, 1.5 Hz, 1H), 5.13 (d, *J* = 12.2 Hz, 1H), 4.43 (s, 2H), 3.95 – 3.91 (m, 5H), 3.90 (s, 3H), 3.50 (d, 2H), 3.24 (s, 2H), 2.87 – 2.68 (m, 2H), 2.47 – 2.41 (m, 2H), 2.27 – 2.12 (m, 4H), 1.79 (s, 3H), 1.77 – 1.68 (m, 7H), 1.67 – 1.61 (m, 2H), 1.57 – 1.50 (m, 2H), 1.48 – 1.42 (m, 2H), 1.41 – 1.35 (m, 2H). ^{13}C NMR (126 MHz, $CDCl_3$) δ : 179.37, 173.50, 171.36, 169.94, 169.07, 166.01, 162.81, 161.70,

160.18, 156.57, 153.42, 137.53, 133.12, 132.58, 132.09, 131.43, 130.28, 128.98, 126.19, 123.15, 122.17, 114.99, 114.04, 107.91, 105.43, 94.86, 72.45, 56.10, 55.48, 51.91, 46.80, 39.05, 36.54, 31.51, 29.69, 29.07, 26.02, 25.75, 25.33, 25.19, 24.94, 23.29, 22.69, 21.64, 17.95. HRMS: calculated for $C_{47}H_{54}N_4O_{11}$ $[M + Na]^+$, 873.3681.; found, 873.3683.

Compound 16 (yellow solid, 46.5%). ^{1}HNMR (500 MHz, Chloroform-*d*) δ: 12.74 (s, 1H), 9.04 (s, 1H), 8.75 (s, 1H), 8.08 (d, 2H), 7.75 (d, J = 7.9 Hz, 1H), 7.65 (d, J = 7.5 Hz, 1H), 7.44 (t, J = 7.7 Hz, 1H), 7.02 (d, 2H), 6.39 (s, 1H), 6.15 (s, 1H), 5.19 (t, J = 6.9, 5.5, 1.6 Hz, 1H), 5.11 (d, J = 12.6 Hz, 1H), 4.41 (s, 2H), 3.93 (d, J = 6.5 Hz, 2H), 3.90 (d, J = 1.6 Hz, 6H), 3.49 (d, J = 7.5 Hz, 2H), 3.25 – 3.19 (m, 2H), 2.82 – 2.65 (m, 2H), 2.44 (s, 2H), 2.14 (s, 4H), 1.79 (s, 3H), 1.76 – 1.71 (m, 2H), 1.71 – 1.65 (m, 6H), 1.62 – 1.56 (m, 2H), 1.54 – 1.48 (m, 2H), 1.42 – 1.34 (m, 5H). ^{13}C NMR (126 MHz, $CDCl_3$) δ: 179.37, 173.69, 171.48, 170.06, 169.11, 168.70, 162.76, 161.63, 160.23, 156.42, 153.42, 137.58, 134.21, 133.18, 132.56, 132.05, 130.30, 128.98, 126.24, 123.24, 122.21, 120.64, 113.99, 107.83, 105.46, 94.82, 77.27, 76.76, 72.79, 56.09, 55.48, 51.92, 46.82, 39.14, 36.57, 36.52, 29.88, 29.69, 29.13, 28.83, 26.13, 25.75, 25.66, 25.48, 25.00, 23.25, 21.64, 17.94. HRMS: calculated for $C_{48}H_{56}N_4O_{11}$ $[M + Na]^+$, 887.3838.; found, 887.3838.

1.3.5 Synthesis of compounds 8 and 17

1.3.5.1 N-(2-(2,6-dioxopiperidin-3-yl)-1-oxoisoindolin-4-yl)-5-(4-((5-hydroxy-2-(4-methoxyphenyl)-8-(3-methylbut-2-en-1-yl)-4-oxo-7-(prop-2-yn-1-yloxy)-4H-chromen-3-yl)oxy)butanamido)pentanamide (compound 8)

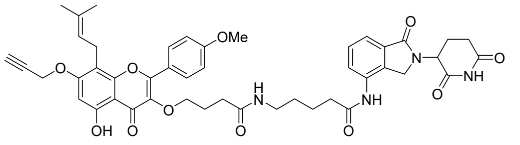

Compound 8

1.3.5.2 N-(6-((2-(2,6-dioxopiperidin-3-yl)-1-oxoisoindolin-4-yl)amino)-6-oxohexyl)-7-((5-hydroxy-7-methoxy-2-(4-methoxyphenyl)-8-(3-methylbut-2-en-1-yl)-4-oxo-4H-chromen-3-yl)oxy)heptanamide (compound 17)

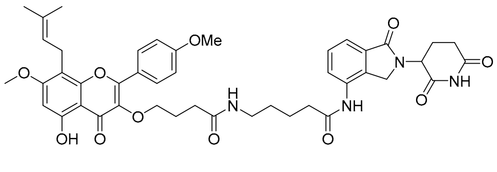

Compound 17

Intermediate **4b/13b** (1.1 equiv), DIPEA (5 equiv), PyBOP (3 equiv) were sequentially dissolved in dry DMF solution at room temperature, monitored by TLC, and after about half an hour, intermediate **s6** (1 equiv) was added and stirring was continued at room temperature, stopping the reaction at about 12 hours. The reaction was quenched by addition of crushed ice, extracted by EA (5 ml × 3), the organic phase was washed sequentially with saturated NH_4Cl and saturated NaCl,

dried over anhydrous Na2SO4, filtered, concentrated under reduced pressure and the **compound 8/17** was purified by PTLC (DCM : MeOH = 15 : 1).

Compound 8 (light yellow solid, 46.3%). ^{1}H NMR (500 MHz, DMSO-d_6) δ: 12.71 (s, 1H), 11.01 (s, 1H), 9.77 (s, 1H), 8.04 (d, 2H), 7.83 – 7.80 (m, 2H), 7.53 – 7.45 (m, 2H), 7.15 (d, 2H), 6.61 (s, 1H), 5.17 – 5.11 (m, 2H), 4.97 (d, J = 2.4 Hz, 2H), 4.38 (q, 2H), 3.96 (t, J = 6.6 Hz, 2H), 3.66 (t, J = 2.3 Hz, 1H), 3.44 (d, J = 7.1 Hz, 2H), 3.06 (q, J = 6.7 Hz, 2H), 2.96 – 2.86 (m, 1H), 2.61 (d, 1H), 2.36 (t, 3H), 2.20 (t, J = 7.5 Hz, 2H), 2.06 – 1.97 (m, 1H), 1.91 – 1.83 (m, 2H), 1.73 (s, 3H), 1.62 (s, 3H), 1.61 – 1.56 (m, 2H), 1.49 – 1.40 (m, 2H). ^{13}C NMR (126 MHz, DMSO) δ: 178.99, 173.28, 171.80, 171.69, 171.49, 168.30, 161.91, 160.72, 159.73, 156.37, 153.30, 137.39, 134.25, 134.13, 133.11, 131.98, 130.52, 129.04, 125.65, 122.88, 122.31, 119.42, 114.72, 108.14, 105.67, 96.72, 79.27, 79.01, 72.23, 57.12, 55.94, 52.03, 46.96, 38.68, 35.86, 32.21, 31.68, 29.22, 26.26, 25.91, 23.10, 23.00, 21.74, 18.29. HRMS: calculated for $C_{46}H_{48}N_4O_{11}$ $[M + H]^+$, 833.3392.; found, 833.3394.

Compound 17 (yellow solid, 47.9%). ^{1}H NMR (500 MHz, DMSO-d_6) δ: 12.74 (s, 1H), 11.02 (s, 1H), 9.77 (s, 1H), 8.04 (d, 2H), 7.82 (t, 2H), 7.52 – 7.45 (m, 2H), 7.15 (d, 2H), 6.54 (s, 1H), 5.18 – 5.10 (m, 2H), 4.35 (q, 2H), 3.95 (t, J = 6.6 Hz, 2H), 3.90 (s, 3H), 3.86 (s, 3H), 3.42 (d, J = 6.9 Hz, 2H), 3.06 (q, J = 6.6 Hz, 2H), 2.96 – 2.87 (m, 1H), 2.60 (d, J = 17.2, 4.4, 2.3 Hz, 1H), 2.41 – 2.31 (m, 3H), 2.20 (t, J = 7.5 Hz, 2H), 2.05 – 1.98 (m, 1H), 1.90 – 1.83 (m, 2H), 1.71 (s, 3H), 1.62 (s, 3H), 1.61 – 1.57 (m, 2H), 1.48 – 1.41 (m, 2H). ^{13}C NMR (126 MHz, DMSO) δ: 178.97, 173.28, 171.78, 171.67, 171.50, 168.29, 162.86, 161.87, 160.12, 156.21, 153.18, 137.28, 134.26, 134.11, 133.12, 131.83, 130.48, 129.04, 125.63, 122.92, 122.51, 119.40, 114.70, 107.44, 105.14, 95.49, 72.22, 56.92, 55.93, 52.01, 46.94, 38.68, 35.86, 32.21, 31.68, 29.23, 26.27, 25.87, 23.10, 23.00, 21.65, 18.21. HRMS: calculated for $C_{44}H_{48}N_4O_{11}$ $[M + Na]^+$, 831.3212.; found, 831.3211.

Supplementary Scheme 5. Synthesis of end products P1-P4

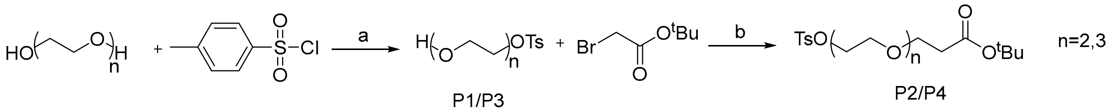

^{a}Reagents and conditions: (a) Et_3N, DCM, 0°C, overnight; (b) TBAB, KOH, DCM, toluene, RT

Supplementary Scheme 6. Synthesis of end products 9-11, 18-20

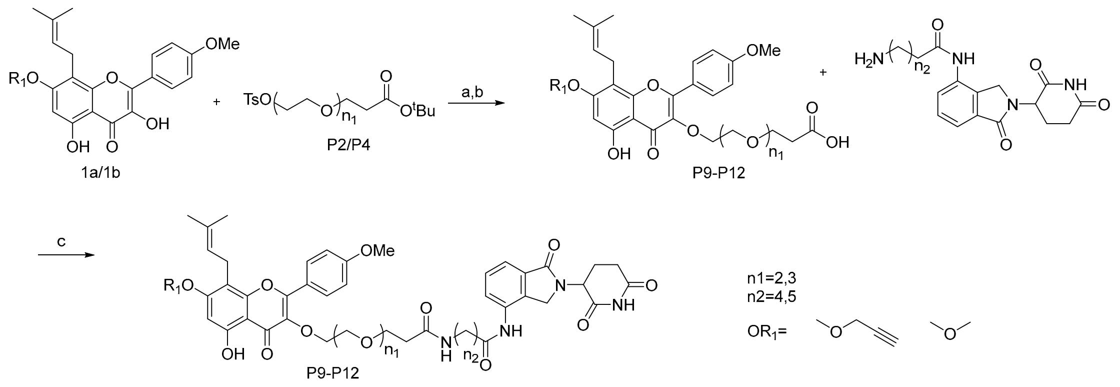

[α]Reagents and conditions: (a) K_2CO_3, DMF, RT; (b) TFA, DCM, RT; (c) PyBOP, DIPEA, DMF, RT

1.3.6 Synthesis of compounds 9-11, 18-20

1.3.6.1 2-(2-hydroxyethoxy)ethyl 4-methylbenzenesulfonate (p1)

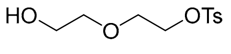

p1

General synthesis procedure for **p1**. Diethylene glycol (1.07 mL, 11.31 mmol), triethylamine (4.72 mL, 33.92 mmol), and DMAP (138 mg, 1.13 mmol) were sequentially dissolved in DCM solution (40 mL), and the reaction was gradually warmed up to room temperature after the batch addition of TsCl (3.23 g, 16.96 mmol) under an ice bath.The TLC was monitored for about 24 h. The reaction was stopped. The reaction solution was adjusted to neutral with 4 M hydrochloric acid solution, extracted three times with DCM, the organic phase was washed with saturated NaCl, dried with anhydrous Na_2SO_4, filtered, and the solvent was removed under reduced pressure and purified by column chromatography (PE : EA = 1:1) to obtain the intermediate **p1** (colorless, transparent liquid, 440 mg, 15.0%).

Intermediate **p1**. ^{1}H NMR (500 MHz, $CHCl_3$-*d*) δ: 7.83 (d, J = 8.4 Hz, 2H), 7.38 (d, J = 8.0 Hz, 2H), 4.22 (t, 2H), 3.75 – 3.67 (m, 4H), 3.59 – 3.53 (m, 2H), 2.48 (s, 3H), 1.27 (s, 1H).

1.3.6.2 tert-butyl 2-(2-(2-(tosyloxy)ethoxy)ethoxy)acetate (p2)

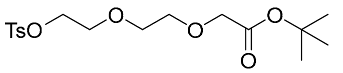

p2

General synthesis procedure for **p2**. Intermediates **p1** (210 mg, 0.807 mmol), TBAB (13 mg, 0.04 mmol), KOH (72.5 mg, 1.29 mmol), and tert-butyl bromoacetate (141 µL, 0.968 mmol) were sequentially added to the toluene solution (1 mL) at room temperature, and the reaction was carried out for about 24 h at room temperature. End. Toluene was removed under reduced pressure, DCM and water were added, extracted with DCM (5 mL x 3), the organic phase was washed with saturated NaCl, dried over anhydrous Na2SO4, filtered, and the solvent was removed under reduced pressure and purified by PTLC (PE : EA = 2 : 1) to give the intermediate p2 (anhydrous clear liquid, 110 mg,

36.4%).

Intermediate **p2**. ^{1}H NMR (500 MHz, Chloroform-*d*) δ: 7.81 (d, J = 7.8 Hz, 2H), 7.36 (d, J = 7.8 Hz, 2H), 4.18 (t, 2H), 3.99 (s, 2H), 3.72 (t, J = 4.4 Hz, 2H), 3.66 (d, 4H), 2.46 (s, 3H), 1.49 (s, 9H). ^{13}C NMR (126 MHz, $CDCl_3$) δ: 169.54, 144.77, 133.06, 129.83, 127.99, 81.61, 70.77, 70.70, 69.26, 69.08, 68.75, 28.13, 21.63.

1.3.6.3 tert-butyl 2-(2-(2-((5-hydroxy-2-(4-methoxyphenyl)-8-(3-methylbut-2-en-1-yl)-4-oxo-7-(prop-2-yn-1-yloxy)-4H-chromen-3-yl)oxy)ethoxy)ethoxy)acetate (p3)

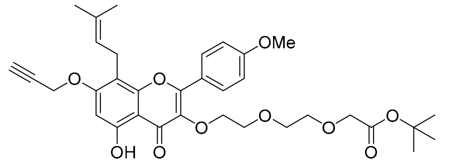

p3

1.3.6.4 tert-butyl 2-(2-(2-((5-hydroxy-7-methoxy-2-(4-methoxyphenyl)-8-(3-methylbut-2-en-1-yl)-4-oxo-4H-chromen-3-yl)oxy)ethoxy)ethoxy)acetate (p4)

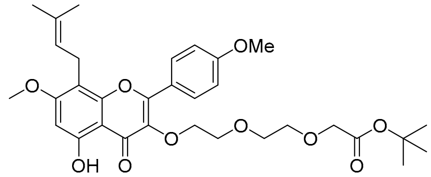

p4

Intermediate **p2** (1 equiv), K2CO3 (1.2 equiv), and intermediate 1a/1b (1 equiv) were sequentially dissolved in dry acetone, stirred at room temperature for a few minutes and then warmed up to 50 °C reflux, and the reaction was stopped after about 6 hours. The reaction was quenched by adding ice water to the reaction solution, extracted by EA (5 ml × 3), the organic phase was washed with saturated NH_4Cl solution and saturated NaCl, dried over anhydrous Na_2SO_4, filtered, concentrated under reduced pressure and purified by PTLC (DCM : MeOH = 35 : 1) to give intermediates **p3/p4**.

Intermediate **p3**: (yellow solid, 41.0%). ^{1}H NMR (500 MHz, Chloroform-*d*) δ: 12.76 (s, 1H), 8.20 (d, J = 8.2 Hz, 2H), 7.04 (d, J = 8.1 Hz, 2H), 6.51 (s, 1H), 5.22 (t, 1H), 4.80 (d, J = 2.3 Hz, 2H), 4.26 (s, 2H), 4.02 (s, 2H), 3.92 (s, 3H), 3.80 (s, 2H), 3.73 (t, 1H), 3.70 – 3.67 (m, 2H), 3.66 – 3.63 (m, 2H), 3.54 (d, J = 7.0 Hz, 2H), 1.81 (s, 3H), 1.71 (s, 3H), 1.49 (s, 9H). ^{13}C NMR (126 MHz, $CDCl_3$) δ: 179.14, 169.63, 161.67, 160.45, 160.13, 156.20, 153.52, 137.46, 132.12, 130.62, 123.20, 122.09, 113.97, 108.44, 106.09, 95.93, 81.53, 77.70, 76.16, 71.54, 70.75, 70.49, 70.42, 69.13, 56.50, 55.44, 28.13, 25.75, 21.78, 18.03.

Intermediate **p4** (yellow solid, 54.0%). ^{1}H NMR (500 MHz, Chloroform-*d*) δ: 8.20 (d, J = 8.6 Hz, 2H), 7.03 (d, J = 8.6 Hz, 2H), 6.42 (s, 1H), 5.21 (t, J = 6.9, 2.9, 1.5 Hz, 1H), 4.26 (t, J = 5.7, 3.2 Hz, 2H), 3.92 (d, J = 1.5 Hz, 6H), 3.81 – 3.78 (m, 2H), 3.70 – 3.67 (m, 2H), 3.65 – 3.62 (m, 2H),

1.80 (s, 3H), 1.71 (s, 3H), 1.49 (s, 9H). ^{13}C NMR (126 MHz, $CDCl_3$) δ: 179.13, 169.64, 162.64, 161.60, 160.37, 156.03, 153.41, 137.35, 131.95, 130.57, 123.31, 122.33, 113.92, 107.69, 105.48, 94.77, 81.52, 71.52, 70.74, 70.47, 70.41, 69.10, 56.05, 55.42, 28.12, 25.74, 21.66, 17.94.

Intermediates **p5** and **p6** are synthesized as **p1** and **p2**.

1.3.6.5 2-(2-(2-hydroxyethoxy)ethoxy)ethyl 4-methylbenzenesulfonate (p5)

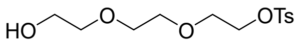

p5

1.3.6.6 tert-butyl 2-(2-(2-(2-(tosyloxy)ethoxy)ethoxy)ethoxy)acetate (p6)

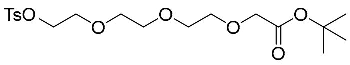

p6

Intermediate **p5** (colorless transparent oily liquid, 22.2%). ^{1}H NMR (500 MHz, $CHCl_3$-*d*) δ: 7.83 (d, 2H), 7.36 (d, *J* = 8.0 Hz, 1H), 4.19 (t, *J* = 4.7 Hz, 2H), 3.74 – 3.71 (m, 4H), 3.63 (s, 4H), 3.59 (t, *J* = 4.2 Hz, 2H), 2.47 (s, 3H), 2.16 (s, 1H).

Intermediate **p6** (anhydrous transparent oily liquid, 40.4%). ^{1}H NMR (500 MHz, Chloroform-*d*) δ: 7.82 (d, 2H), 7.36 (d, 2H), 4.18 (t, 2H), 4.02 (s, 2H), 3.73 – 3.69 (m, 4H), 3.69 – 3.66 (m, 2H), 3.61 (s, 4H), 2.47 (s, 3H), 1.70 (s, 2H), 1.49 (s, 9H).

The synthesis of intermediates **p7**/**p8** is similar to that of **p3**/**p4**.

1.3.6.7 tert-butyl 2-(2-(2-(2-((5-hydroxy-2-(4-methoxyphenyl)-8-(3-methylbut-2-en-1-yl)-4-oxo-7-(prop-2-yn-1-yloxy)-4H-chromen-3-yl)oxy)ethoxy)ethoxy)ethoxy)acetate (p7)

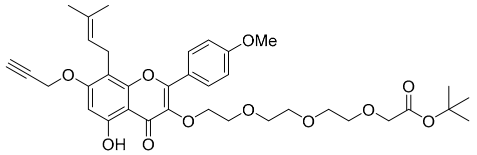

p7

1.3.6.8 tert-butyl 2-(2-(2-(2-((5-hydroxy-7-methoxy-2-(4-methoxyphenyl)-8-(3-methylbut-2-en-1-yl)-4-oxo-4H-chromen-3-yl)oxy)ethoxy)ethoxy)ethoxy)acetate (p7)

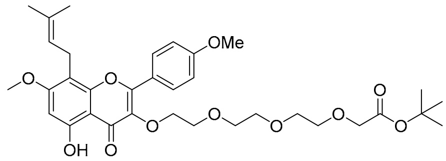

p8

Intermediate **p7** (yellow solid, 49.2%). ^{1}H NMR (500 MHz, Chloroform-*d*) δ: 8.20 (d, *J* = 8.5 Hz, 2H), 7.03 (d, *J* = 8.4 Hz, 2H), 6.51 (s, 1H), 5.25 – 5.20 (m, 1H), 4.80 (d, *J* = 2.4 Hz, 2H), 4.26 (t, *J* = 5.7, 3.2 Hz, 2H), 4.03 (s, 2H), 3.92 (s, 3H), 3.78 (t, *J* = 4.6 Hz, 2H), 3.74 – 3.71 (m, 2H), 3.70 – 3.67 (m, 2H), 3.65 – 3.60 (m, 4H), 3.54 (d, *J* = 7.0 Hz, 2H), 2.58 (t, *J* = 2.4 Hz, 1H), 1.81 (s, 3H), 1.71 (s, 3H), 1.48 (s, 9H). ^{13}C NMR (126 MHz, $CDCl_3$) δ: 179.16, 169.66, 161.66, 160.45, 160.12,

156.20, 153.51, 137.44, 132.13, 130.62, 123.19, 122.08, 113.93, 108.43, 106.07, 95.92, 81.54, 77.69, 76.18, 71.54, 70.73, 70.61, 70.43, 70.35, 69.07, 56.49, 55.43, 28.12, 25.76, 21.78, 18.03.

Intermediate **p8** (yellow solid, 44.4%). ^{1}H NMR (500 MHz, Chloroform-*d*) δ: 12.77 (s, 1H), 8.20 (d, J = 8.7 Hz, 2H), 7.03 (d, 2H), 6.42 (s, 1H), 5.21 (t, 1H), 4.26 (t, 2H), 4.03 (s, 2H), 3.92 (d, J = 2.2 Hz, 6H), 3.78 (t, 2H), 3.73 – 3.67 (m, 4H), 3.62 (tt, J = 4.8, 2.9 Hz, 4H), 3.52 (d, J = 7.0 Hz, 2H), 1.80 (s, 3H), 1.71 (s, 3H), 1.48 (s, 9H). ^{13}C NMR (126 MHz, $CDCl_3$) δ: 179.15, 169.65, 162.63, 161.58, 160.36, 156.02, 153.39, 137.34, 131.97, 130.58, 123.30, 122.32, 113.89, 107.68, 105.47, 94.76, 81.52, 71.53, 70.73, 70.61, 70.42, 70.35, 69.06, 56.06, 55.42, 28.11, 25.75, 21.66, 17.95.

Synthesis of intermediates **p9~p12**

1.3.6.9 2-(2-(2-((5-hydroxy-2-(4-methoxyphenyl)-8-(3-methylbut-2-en-1-yl)-4-oxo-7-(prop-2-yn-1-yloxy)-4H-chromen-3-yl)oxy)ethoxy)ethoxy)acetic acid (p9)

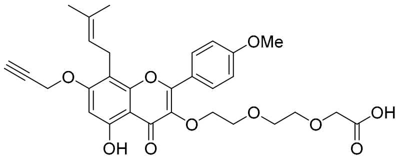

p9

1.3.6.10 2-(2-(2-((5-hydroxy-7-methoxy-2-(4-methoxyphenyl)-8-(3-methylbut-2-en-1-yl)-4-oxo-4H-chromen-3-yl)oxy)ethoxy)ethoxy)acetic acid (p10)

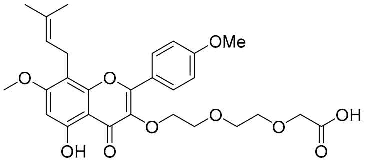

p10

1.3.6.11 2-(2-(2-(2-((5-hydroxy-2-(4-methoxyphenyl)-8-(3-methylbut-2-en-1-yl)-4-oxo-7-(prop-2-yn-1-yloxy)-4H-chromen-3-yl)oxy)ethoxy)ethoxy)ethoxy)acetic acid (p11)

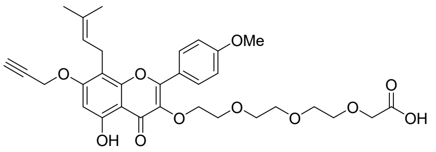

p11

1.3.6.12 2-(2-(2-(2-((5-hydroxy-7-methoxy-2-(4-methoxyphenyl)-8-(3-methylbut-2-en-1-yl)-4-oxo-4H-chromen-3-yl)oxy)ethoxy)ethoxy)ethoxy)acetic acid (p12)

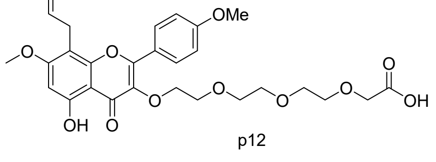

p12

Under the ice bath, the intermediate **p3/p4/p7/p8** (1 equiv) was dissolved in DCM solution, anisole (5~6 equiv) was added as a protective agent, stirred well and then TFA (15%) was slowly added dropwise to the solution, the reaction was slowly warmed up and moved to room temperature,

and the reaction was complete for about 12 hours. After the reaction was stopped, the solution was diluted with water, extracted by DCM (5 mL×3), the organic phase was washed with saturated NaCl, dried with anhydrous Na2SO4, filtered and concentrated under reduced pressure to obtain intermediates **p9~p12**, which were directly used in the next reaction without purification.

Intermediate **p9**: yellow oily liquid, 98.3%.

Intermediate **p10**: yellow oily liquid, 93.6%.

Intermediate **p11**: yellow oily liquid, 96.4%.

Intermediate **p12**: yellow oily liquid, 95.8%

1.3.7 Synthesis of compounds 9/18/11/20

1.3.7.1 N-(2-(2,6-dioxopiperidin-3-yl)-1-oxoisoindolin-4-yl)-6-(2-(2-(2-((5-hydroxy-2-(4-methoxyphenyl)-8-(3-methylbut-2-en-1-yl)-4-oxo-7-(prop-2-yn-1-yloxy)-4H-chromen-3-yl)oxy)ethoxy)ethoxy)acetamido)hexanamide (Compound 9)

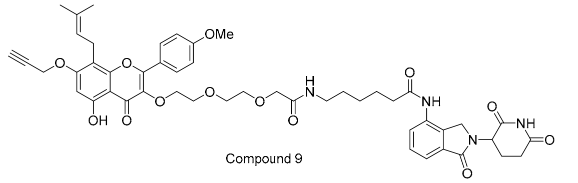

Compound 9

1.3.7.2 N-(2-(2,6-dioxopiperidin-3-yl)-1-oxoisoindolin-4-yl)-6-(2-(2-(2-((5-hydroxy-7-methoxy-2-(4-methoxyphenyl)-8-(3-methylbut-2-en-1-yl)-4-oxo-4H-chromen-3-yl)oxy)ethoxy)ethoxy)acetamido)hexanamide (Compound 18)

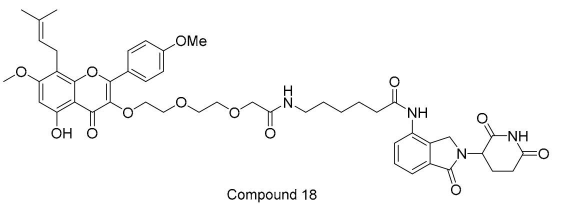

Compound 18

1.3.7.3 N-(2-(2,6-dioxopiperidin-3-yl)-1-oxoisoindolin-4-yl)-6-(2-(2-(2-(2-((5-hydroxy-2-(4-methoxyphenyl)-8-(3-methylbut-2-en-1-yl)-4-oxo-7-(prop-2-yn-1-yloxy)-4H-chromen-3-yl)oxy)ethoxy)ethoxy)ethoxy)acetamido)hexanamide (Compound 11)

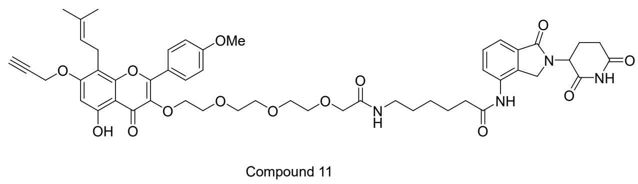

Compound 11

1.3.7.4 N-(2-(2,6-dioxopiperidin-3-yl)-1-oxoisoindolin-4-yl)-6-(2-(2-(2-(2-((5-hydroxy-7-methoxy-2-(4-methoxyphenyl)-8-(3-methylbut-2-en-1-yl)-4-oxo-4H-chromen-3-yl)oxy)ethoxy)ethoxy)ethoxy)acetamido)hexanamide (Compound 20)

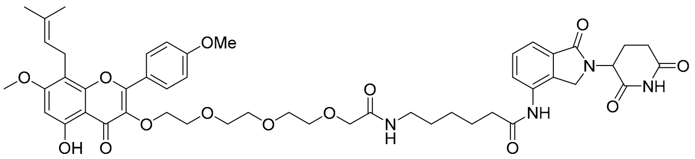

Compound 20

The lower intermediates **p9/p10 /p11/p12** (1.1 equiv), DIPEA (5 equiv), PyBOP (3 equiv) were sequentially dissolved in dry DMF solution at room temperature and monitored by TLC for about half an hour, then intermediate **s3** (1 equiv) was added, and the reaction was continued at room temperature, and was stopped after about 12 hours. The reaction was quenched by the addition of crushed ice, extracted by EA (5 mL x 3), the organic phase was washed sequentially with saturated NH_4Cl and saturated NaCl, dried with anhydrous Na2SO4, filtered, concentrated under reduced pressure and purified by PTLC (DCM : MeOH= 13 : 1~15 : 1) to **compound 9/18/11/20**.

Compound 9 (light yellow solid, 44.5%). ^{1}H NMR (500 MHz, Chloroform-*d*) δ: 12.69 (s, 1H), 8.96 (s, 1H), 8.59 (s, 1H), 8.13 (d, 2H), 7.73 (d, *J* = 7.9 Hz, 1H), 7.65 (d, *J* = 7.5 Hz, 1H), 7.43 (t, *J* = 7.7 Hz, 1H), 7.25 (t, *J* = 5.9 Hz, 1H), 7.02 (d, 2H), 6.38 (s, 1H), 5.13 (dd, *J* = 13.3, 5.2 Hz, 1H), 4.41 (s, 2H), 4.20 (t, 2H), 3.95 (s, 2H), 3.90 (d, *J* = 2.0 Hz, 6H), 3.75 (t, 2H), 3.66 – 3.57 (m, 4H), 3.49 (s, 1H), 3.25 (q, *J* = 6.8 Hz, 2H), 2.90 – 2.84 (m, 2H), 2.82 – 2.71 (m, 2H), 2.40 (t, *J* = 7.5 Hz, 2H), 2.11 – 2.04 (m, 2H), 1.76 – 1.69 (m, 2H), 1.67 (s, 6H), 1.57 – 1.50 (m, 2H), 1.40 – 1.33 (m, 2H). ^{13}C NMR (126 MHz, $CDCl_3$) δ: 179.04, 171.88, 171.53, 170.28, 169.99, 169.10, 162.96, 161.91, 160.52, 156.40, 153.30, 137.18, 134.15, 133.12, 132.55, 130.30, 128.94, 126.13, 122.83, 120.66, 115.63, 107.01, 105.24, 94.95, 77.22, 71.51, 70.94, 70.35, 70.32, 69.94, 56.16, 55.45, 51.91, 46.71, 39.32, 38.58, 29.68, 28.94, 26.06, 25.56, 24.96, 23.25, 17.01. HRMS: calculated for $C_{49}H_{54}N_4O_{13}$ $[M + Na]^+$, 929.3580.; found, 929.3578.

Compound 18 (light yellow solid, 32.3%). ^{1}H NMR (500 MHz, Chloroform-*d*) δ: 12.71 (s, 1H), 8.80 (s, 1H), 8.44 (s, 1H), 8.14 (d, 2H), 7.74 (d, *J* = 8.0 Hz, 1H), 7.67 (d, *J* = 7.5 Hz, 1H), 7.44 (t, *J* = 7.7 Hz, 1H), 7.20 (s, 1H), 7.05 – 7.00 (m, 2H), 6.39 (s, 1H), 5.15 (dd, *J* = 13.3, 5.1 Hz, 1H), 4.42 (s, 2H), 4.21 (t, *J* = 5.5, 3.6 Hz, 2H), 3.96 (s, 2H), 3.90 (d, *J* = 1.7 Hz, 6H), 3.74 (t, *J* = 5.5, 3.6 Hz, 2H), 3.65 – 3.56 (m, 4H), 3.26 (q, *J* = 6.7 Hz, 2H), 2.90 – 2.84 (m, 2H), 2.83 – 2.71 (m, 2H), 2.42 (t, *J* = 7.3 Hz, 2H), 2.20 – 2.12 (m, 2H), 1.77 – 1.70 (m, 2H), 1.67 (s, 6H), 1.57 – 1.51 (m, 2H), 1.40 – 1.32 (m, 3H). ^{13}C NMR (126 MHz, $CDCl_3$) δ: 179.04, 171.76, 171.36, 170.28, 169.87, 169.02, 162.95, 161.90, 160.51, 156.37, 153.29, 137.17, 134.10, 133.07, 132.59, 130.49, 130.32, 128.97, 126.06, 122.82, 120.72, 115.63, 114.12, 107.00, 105.24, 94.93, 71.50, 71.00, 70.32, 70.26, 69.91,

56.18, 55.47, 51.89, 46.69, 39.31, 38.57, 36.52, 31.53, 29.01, 26.10, 25.57, 24.95, 23.30. HRMS: calculated for $C_{47}H_{54}N_4O_{13}$ $[M + H]^+$, 905.3580.; found, 905.3582.

Compound 11 (light yellow solid, 25.7%). ^{1}H NMR (500 MHz, Chloroform-*d*) δ: 12.68 (s, 1H), 8.85 (s, 1H), 8.51 (s, 1H), 8.17 – 8.13 (m, 2H), 7.77 (d, *J* = 7.9 Hz, 1H), 7.65 (d, *J* = 7.5 Hz, 1H), 7.43 (t, *J* = 7.7 Hz, 1H), 7.03 (d, 2H), 6.49 (s, 1H), 5.14 (d, *J* = 13.2, 4.8 Hz, 1H), 4.79 (d, *J* = 2.4 Hz, 2H), 4.44 (s, 2H), 4.18 (t, *J* = 4.5 Hz, 2H), 3.96 (s, 2H), 3.91 (s, 3H), 3.76 (dd, *J* = 5.8, 3.5 Hz, 2H), 3.63 (s, 8H), 3.25 (d, *J* = 6.8 Hz, 2H), 2.93 – 2.87 (m, 2H), 2.85 – 2.71 (m, 2H), 2.60 (t, *J* = 2.3 Hz, 1H), 2.43 (s, 2H), 2.12 – 2.05 (m, 4H), 1.78 – 1.72 (m, 2H), 1.68 (s, 6H), 1.58 – 1.51 (m, 2H), 1.42 – 1.34 (m, 2H). ^{13}C NMR (126 MHz, $CDCl_3$) δ: 179.08, 171.43, 170.43, 169.95, 169.04, 161.92, 160.70, 160.33, 156.45, 156.03, 153.40, 138.30, 137.30, 134.04, 133.14, 132.57, 130.37, 128.94, 126.02, 122.77, 120.59, 115.64, 114.12, 107.61, 105.84, 96.04, 77.36, 76.46, 71.51, 70.88, 70.49, 70.37, 70.29, 70.19, 70.15, 56.59, 55.45, 51.92, 46.79, 39.50, 38.56, 36.59, 31.51, 29.70, 28.95, 26.03, 24.99, 23.28, 17.13. HRMS: calculated for $C_{51}H_{58}N_4O_{14}$ $[M + Na]^+$, 973.3842.; found, 973.3848.

Compound 20 (yellow solid, 32.7%). ^{1}H NMR (500 MHz, Chloroform-*d*) δ: 12.68 (s, 1H), 8.91 (s, 1H), 8.59 (s, 1H), 8.14 (d, 2H), 7.77 (d, *J* = 7.9 Hz, 1H), 7.65 (d, *J* = 7.5 Hz, 1H), 7.33 (s, 1H), 7.03 (d, 2H), 6.39 (s, 1H), 5.13 (dd, *J* = 13.0, 5.0 Hz, 1H), 4.43 (s, 2H), 4.18 (t, 2H), 3.96 (s, 2H), 3.90 (s, 6H), 3.76 (t, 2H), 3.63 (d, *J* = 2.7 Hz, 8H), 3.50 (q, *J* = 7.0 Hz, 1H), 3.25 (q, *J* = 6.7 Hz, 2H), 2.91 – 2.85 (m, 2H), 2.85 – 2.69 (m, 2H), 2.46 – 2.40 (m, 2H), 2.19 – 2.11 (m, 2H), 1.78 – 1.70 (m, 2H), 1.59 – 1.51 (m, 2H), 1.42 – 1.33 (m, 2H). ^{13}C NMR (126 MHz, $CDCl_3$) δ: 179.07, 171.95, 171.47, 170.41, 169.96, 169.06, 162.90, 161.85, 160.56, 156.29, 153.30, 141.08, 137.20, 134.05, 133.17, 132.55, 130.33, 128.92, 126.04, 122.88, 120.59, 115.63, 114.09, 106.94, 105.25, 94.91, 71.52, 70.87, 70.50, 70.36, 70.30, 70.19, 70.16, 56.16, 55.44, 51.92, 46.76, 39.36, 38.58, 36.56, 31.51, 28.94, 26.04, 25.56, 25.00, 23.26. HRMS: calculated for $C_{49}H_{58}N_4O_{14}$ $[M + Na]^+$, 949.3842.; found, 949.3842.

1.3.8 Synthesis of compound 10/19

1.3.8.1 N-(2-(2,6-dioxopiperidin-3-yl)-1-oxoisoindolin-4-yl)-5-(2-(2-(2-((5-hydroxy-2-(4-methoxyphenyl)-8-(3-methylbut-2-en-1-yl)-4-oxo-7-(prop-2-yn-1-yloxy)-4H-chromen-3-yl)oxy)ethoxy)ethoxy)acetamido)pentanamide (Compound 10;LJ-11)

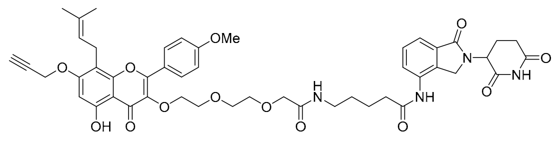

Compound 10 (LJ-41)

1.3.8.2 N-(2-(2,6-dioxopiperidin-3-yl)-1-oxoisoindolin-4-yl)-5-(2-(2-(2-((5-hydroxy-7-methoxy-2-(4-methoxyphenyl)-8-(3-methylbut-2-en-1-yl)-4-oxo-4H-chromen-3-yl)oxy)ethoxy)ethoxy)acetamido)pentanamide (Compound 19)

Intermediate **p9/p10** (1.1 equiv), DIEA (5 equiv), and PyBOP (3 equiv) were sequentially dissolved in dry DMF solution at room temperature and monitored by TLC for about half an hour, then intermediate s6 (1 equiv) was added, and the reaction was stopped by continuing the reaction

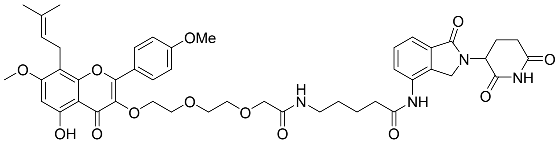

Compound 19

at room temperature for about 24 hours. The reaction was quenched by addition of crushed ice, extracted by EA (5 mL x 3), the organic phase was washed sequentially with saturated NH_4Cl and saturated NaCl, dried over anhydrous Na_2SO_4, filtered, concentrated under reduced pressure and purified by PTLC (DCM : MeOH = 14 : 1) to give **compounds 10/19**.

Compound10 (light yellow solid, 53.4%). ^{1}H NMR (500 MHz, Chloroform-*d*) δ: 12.70 (s, 1H), 8.65 (s, 1H), 8.56 (s, 1H), 8.14 (d, 2H), 7.83 (d, *J* = 7.9 Hz, 1H), 7.65 (d, *J* = 7.4 Hz, 1H), 7.43 (t, *J* = 7.8 Hz, 1H), 7.34 (t, 1H), 7.03 (d, 2H), 6.49 (s, 1H), 5.19 (dd, *J* = 13.2, 5.1 Hz, 1H), 4.79 (d, *J* = 2.4 Hz, 2H), 4.50 – 4.41 (m, 2H), 4.20 (t, *J* = 6.0, 3.3 Hz, 2H), 4.00 (s, 2H), 3.90 (s, 3H), 3.75 (t, *J* = 5.9, 3.3 Hz, 2H), 3.68 (t, *J* = 5.8, 2.7 Hz, 2H), 3.62 (t, *J* = 5.7, 2.9 Hz, 2H), 3.34 (t, *J* = 20.1, 6.7 Hz, 2H), 2.93 – 2.87 (m, 2H), 2.60 (t, *J* = 2.3 Hz, 1H), 2.55 – 2.32 (m, 4H), 2.22 – 2.16 (m, 1H), 2.12 – 2.07 (m, 2H), 1.80 – 1.72 (m, 2H), 1.68 (s, 6H), 1.64 – 1.58 (m, 2H). ^{13}C NMR (126 MHz, $CDCl_3$) δ: 179.06, 171.91, 171.33, 170.76, 169.80, 169.04, 161.98, 160.76, 160.30, 156.57, 153.40, 137.36, 133.49, 133.27, 132.49, 130.35, 128.99, 125.48, 122.71, 120.45, 114.16, 107.69, 105.85, 96.08, 76.48, 71.62, 71.08, 70.35, 70.31, 69.95, 56.61, 55.46, 51.85, 46.53, 39.47, 37.62, 31.53, 29.70, 28.85, 25.55, 23.33, 22.80. HRMS: calculated for $C_{48}H_{52}N_4O_{13}$ $[M + Na]^+$, 915.3423.; found, 915.3416.

Compound 19 (light yellow solid, 35.2%). ^{1}H NMR (500 MHz, Chloroform-*d*) δ: 12.69 (s, 1H), 8.70 (s, 1H), 8.62 (s, 1H), 8.13 (d, 2H), 7.83 (d, *J* = 7.9 Hz, 1H), 7.65 (d, *J* = 7.5 Hz, 1H), 7.46 – 7.41 (m, 1H), 7.37 (s, 1H), 7.03 (d, 2H), 6.39 (s, 1H), 5.18 (dd, *J* = 13.0, 6.4 Hz, 1H), 4.50 – 4.41 (m, 2H), 4.25 – 4.16 (m, 2H), 4.01 (s, 1H), 3.90 (d, *J* = 5.5 Hz, 6H), 3.77 – 3.73 (m, 2H), 3.70 – 3.66 (m, 2H), 3.64 – 3.58 (m, 2H), 3.41 – 3.23 (m, 2H), 2.91 – 2.74 (m, 4H), 2.55 – 2.30 (m, 4H), 2.11 – 2.05 (m, 2H), 1.78 – 1.71 (m, 2H), 1.67 (s, 6H), 1.64 – 1.58 (m, 2H). ^{13}C NMR (126 MHz, $CDCl_3$) δ: 179.04, 171.95, 171.35, 170.77, 169.82, 169.05, 162.97, 161.91, 160.52, 156.41, 153.30, 137.25, 133.50, 133.29, 132.60, 132.48, 130.31, 128.98, 125.52, 122.81, 120.43, 115.63, 114.13, 107.02, 105.25, 94.95, 71.60, 71.09, 70.35, 70.30, 69.94, 56.18, 55.45, 51.85, 46.55, 39.33, 37.69,

31.53, 29.69, 28.83, 25.57, 23.32, 22.79. HRMS: calculated for $C_{46}H_{52}N_4O_{13}$ $[M + Na]^+$, 891.3423.; found, 891.3424.

1.3.9 Synthesis of VHL-type ligand V0

Supplementary Scheme 7. Synthesis of VHL-type ligand V0

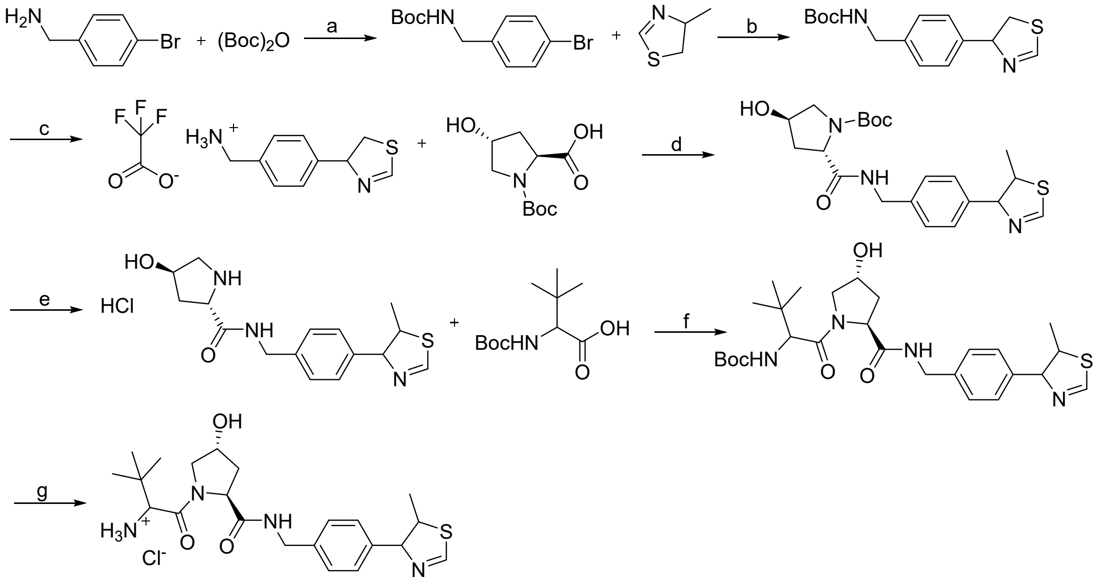

^{a}Reagents and conditions: (a)EA/H2O, NaHCO3, RT; (b) KOAc, Pd(OAc)2, DMF, N2, 90°C; (c) TFA, DCM; (d) HATU, DIPEA, DMF; (e) HCl/EtOH; (f) HATU, DIPEA, DMF; (g) HCl/EtOH

1.3.9.1 tert-butyl (4-bromobenzyl)carbamate (v1)

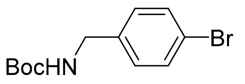

v1

General synthesis procedure for **v1**, P-bromobenzylamine (681 μL, 5.37 mmol) was dissolved in a mixed solution (40 mL) of EA/H_2O (V : V=1:1), stirred well at room temperature, then $NaHCO_3$ (91 mg, 1.07 mmol), Boc_2O (1.36 mL, 5.91 mmol) were added sequentially, and the reaction was monitored by TLC for about 2 hours. The reaction was stopped. EA extraction (5 mL × 3), the organic phase was washed with saturated NaCl, dried with anhydrous Na2SO4, filtered, and concentrated under reduced pressure to give intermediate **v1** (white solid, 1.365 g, 88.6%), which was used directly in the next step without purification.

Intermediate **v1**. ^{1}H NMR (500 MHz, Chloroform-*d*) δ: 7.47 (d, J = 8.4 Hz, 2H), 7.19 (d, 2H), 4.88 (s, 1H), 4.28 (s, 2H), 1.48 (s, 9H)

1.3.9.2 tert-butyl (4-(4-methylthiazol-5-yl)benzyl)carbamate (v2)

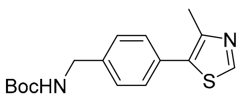

v2

General synthesis procedure for **v2**, Intermediate V1 (1 g, 3.49 mmol), KOAc (686 mg, 6.99 mmol) and palladium acetate (1%, 8 mg, 0.03 mmol) were added to a solution of dry DMF (12 mL)

under N_2 protection, 4-methylthiazole (636 μL, 6.99 mmol) was added, and the reaction was warmed to 90°C Reflow and stop the reaction after about 4 hours. The reaction was quenched by the addition of crushed ice, extracted by EA (5 mL×3), the organic phase was washed with saturated NH_4Cl and saturated NaCl, dried with anhydrous Na_2SO_4, filtered, and the crude product obtained after concentration under reduced pressure was purified by column chromatography (PE:EA = 5:1) to obtain the intermediate **v2** (white solid, 864 mg,, 81.5%).

1.3.9.3 (4-(4-methylthiazol-5-yl)phenyl)methanaminium (v3)

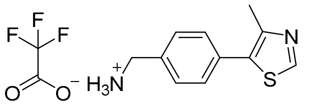

v3

General synthesis procedure for **v3**, Intermediate **v2** (500 mg, 1.64 mmol) was dissolved in a solution of DCM (2.5 mL), and TFA ($V_{TFA} : V_{DCM} = 1 : 1$) was slowly added dropwise to the reaction solution with stirring at room temperature. The reaction was followed by TLC and was stopped after about 1 hour. The solvent was removed under reduced pressure and left to stand to obtain the intermediate **v3**, which was carried out directly to the next step of the reaction without purification.

Intermediate **v3** (white to slightly yellow solid, 312 mg, 92.5%): 1H NMR (500 MHz, DMSO-d6) δ : 9.04 (s, 1H), 8.35 (s, 2H), 7.57 (s, 4H), 4.10 (s, 2H), 2.47 (s, 3H).

1.3.9.4 tert-butyl (2S,4R)-4-hydroxy-2-((4-(4-methylthiazol-5-yl)benzyl)carbamoyl)pyrrolidine-1-carboxylate (v4)

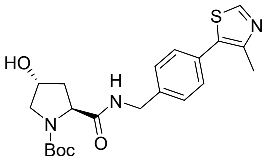

v4

General synthesis procedure for **v4**, Boc-L-hydroxyproline (240 mg, 1.04 mmol), DIPEA (4.71 mmol, 821 L), and HATU (430 mg, 1.13 mmol) were sequentially dissolved in a dry DMF (5 mL) solution under an ice bath, and ice was added to maintain the temperature of the reaction solution at 0~5 °C, and the generation of active esters was monitored by TLC. Intermediate **v3** (300 mg, 0.94 mmol) was added and the reaction was stopped at room temperature for about 12 hours. The reaction was quenched with ice water, extracted with EA (5 mL×3), the organic phase was washed with saturated NH_4Cl and saturated NaCl, dried with anhydrous Na_2SO_4, filtered, concentrated under reduced pressure, and then purified by PTLC (DCM: MeOH = 20:1) to obtain intermediate v4 (white viscous liquid, 298 mg, 75.7%).

1.3.9.5 (2S,4R)-4-hydroxy-N-(4-(4-methylthiazol-5-yl)benzyl)pyrrolidine-2-carboxamide (v5)

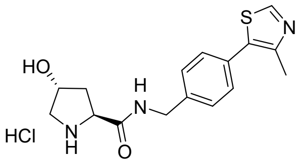

v5

General synthesis procedure for **v5**, Intermediate **v4** (250 mg, 0.6 mmol) was dissolved in ethanol hydrochloride (5 mL) solution and stirred at room temperature, a white solid gradually precipitated from the reaction solution, and the reaction was stopped after about 12 hours. Concentrate under reduced pressure to remove the solvent and dry under vacuum to obtain intermediate **v5**, which was used directly in the next reaction without purification.

Intermediate **v5** (white to slightly pink solid, 181 mg, 95.2%). ^{1}H NMR (500 MHz, DMSO-d_6) δ:10.28 – 10.16 (m, 1H), 9.34 (t, J = 5.8, 5.0 Hz, 1H), 9.12 (s, 1H), 8.72 (d, J = 34.9 Hz, 1H), 7.48 (d, J = 7.9 Hz, 2H), 7.40 (d, J = 7.9 Hz, 2H), 4.46 – 4.37 (m, 4H), 3.38 – 3.32 (m, 1H), 3.10 (d, J = 12.0, 7.3 Hz, 1H), 2.47 (s, 3H), 2.38 – 2.33 (m, 1H), 1.97 – 1.89 (m, 1H). ^{13}C NMR (126 MHz, DMSO) δ 168.45, 152.44, 147.75, 139.03, 131.78, 130.41, 129.45, 128.33, 69.51, 58.46, 53.71, 42.55, 39.13, 16.17.

1.3.9.6 tert-butyl ((S)-1-((2S,4R)-4-hydroxy-2-((4-(4-methylthiazol-5-yl)benzyl)carbamoyl)pyrrolidin-1-yl)-3,3-dimethyl-1-oxobutan-2-yl)carbamate (v6)

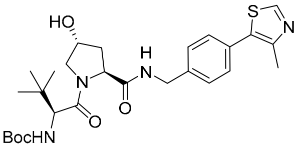

v6

General synthesis procedure for **v6**, N-Boc-L-tert-leucine (150 mg, 0.65 mmol), DIPEA (514 μL, 1.67 mmol), and HATU (270 mg, 0.71 mmol) were sequentially dissolved in a dry DMF (5 mL) solution, and after TLC to monitor the formation of the active ester, intermediate **v5** (208 mg. 0.59 mmol) was added and the reaction was stopped by stirring at room temperature for about 24 hours. The reaction was quenched by adding ice water, extracted by EA (5 mL×3), the organic phase was washed with saturated NH_4Cl and saturated NaCl sequentially, dried with anhydrous Na_2SO_4, filtered, concentrated under reduced pressure and purified by PTLC (DCM: MeOH = 15: 1) to obtain the intermediate **v6** (white viscous liquid, 236 mg, 75.4%).

1.3.9.7 (S)-1-((2S,4R)-4-hydroxy-2-((4-(4-methylthiazol-5-yl)benzyl)carbamoyl)pyrrolidin-1-yl)-3,3-dimethyl-1-oxobutan-2-aminium (v0)

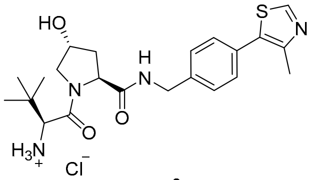

v0

General synthesis procedure for **v0**, Intermediate **v6** was dissolved in ethanol hydrochloride (236 mg, 0.44 mmol) solution and stirred at room temperature, a white solid gradually precipitated from the reaction solution and the reaction was stopped after about 24 hours. The solvent was removed by concentration under reduced pressure and dried under vacuum to obtain Intermediate **v0**, which was used directly in the next reaction without purification.

Intermediate **v0** (white solid, 186.7 mg, 89.9%). ^{1}H NMR (500 MHz, DMSO-d_6) δ: 9.09 (s, 1H), 8.77 (t, J = 6.0 Hz, 1H), 8.18 (s, 3H), 4.56 (t, 2H), 4.43 (dd, J = 15.8, 6.5 Hz, 1H), 4.38 (s, 1H), 4.25 (dd, J = 15.8, 5.6 Hz, 1H), 3.91 (q, J = 5.5 Hz, 1H), 3.79 (d, J = 11.0 Hz, 1H), 3.56 (dd, J = 11.0, 3.9 Hz, 1H), 2.46 (s, 3H), 2.16 – 2.10 (m, 1H), 1.92 – 1.86 (m, 1H). ^{13}C NMR (126 MHz, DMSO) δ: 171.98, 167.21, 152.26, 147.69, 140.01, 131.88, 129.94, 129.16, 127.90, 69.45, 59.46, 58.49, 57.01, 42.14, 38.57, 34.88, 26.48, 16.19.

Supplementary Scheme 8. Synthesis of end products 21-24

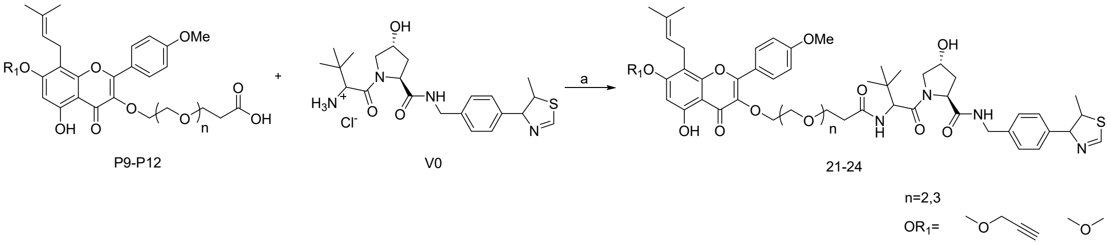

$^{\alpha}$Reagents and conditions: PyBOP, DIPEA, DMF, RT

1.3.10 Synthesis of compounds 21~24

1.3.10.1 (2S,4R)-4-hydroxy-1-((S)-2-(2-(2-(2-((5-hydroxy-2-(4-methoxyphenyl)-8-(3-methylbut-2-en-1-yl)-4-oxo-7-(prop-2-yn-1-yloxy)-4H-chromen-3-yl)oxy)ethoxy)ethoxy)acetamido)-3,3-dimethylbutanoyl)-N-(4-(4-methylthiazol-5-yl)benzyl)pyrrolidine-2-carboxamide (Compound 21)

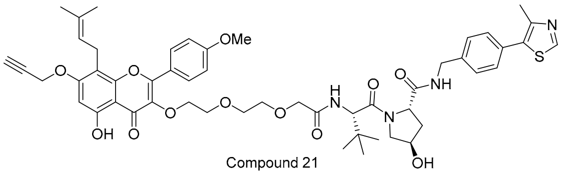

Compound 21

1.3.10.2 (2S,4R)-1-((S)-2-(tert-butyl)-14-((5-hydroxy-2-(4-methoxyphenyl)-8-(3-methylbut-2-en-1-yl)-4-oxo-7-(prop-2-yn-1-yloxy)-4H-chromen-3-yl)oxy)-4-oxo-6,9,12-trioxa-3-azatetradecanoyl)-4-hydroxy-N-(4-(4-methylthiazol-5-yl)benzyl)pyrrolidine-2-carboxamide (Compound 22)

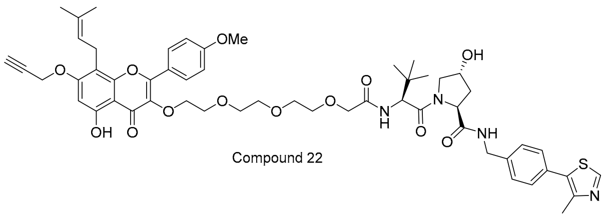

Compound 22

1.3.10.3 (2S,4R)-4-hydroxy-1-((S)-2-(2-(2-(2-((5-hydroxy-7-methoxy-2-(4-methoxyphenyl)-8-(3-methylbut-2-en-1-yl)-4-oxo-4H-chromen-3-yl)oxy)ethoxy)ethoxy)acetamido)-3,3-dimethylbutanoyl)-N-(4-(4-methylthiazol-5-yl)benzyl)pyrrolidine-2-carboxamide (Compound 23)

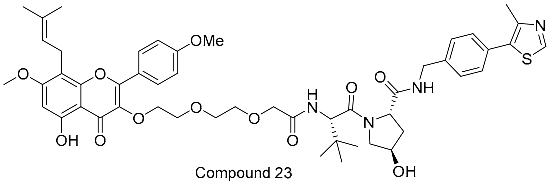

Compound 23

1.3.10.4 (2S,4R)-1-((S)-2-(tert-butyl)-14-((5-hydroxy-7-methoxy-2-(4-methoxyphenyl)-8-(3-methylbut-2-en-1-yl)-4-oxo-4H-chromen-3-yl)oxy)-4-oxo-6,9,12-trioxa-3-azatetradecanoyl)-4-hydroxy-N-(4-(thiazol-5-yl)benzyl)pyrrolidine-2-carboxamide (Compound 24)

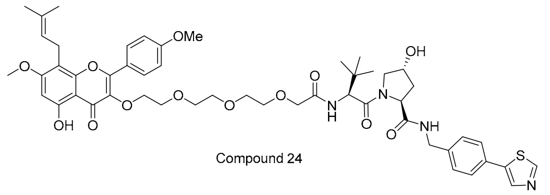

Compound 24

General synthesis procedure for **compounds 21~24**, Intermediates **p9/p10/p11/p12** (1.1 equiv), DIPEA (5 equiv), and PyBOP (3 equiv) were sequentially dissolved in dry DMF solution and stirred at room temperature for 2 h. After 2 h, intermediate **v0** (1equiv) was added, and the reaction was continued at room temperature, stopping after about 12 h. The reaction was quenched with ice water. The reaction was quenched by adding ice water to the reaction solution, extracted by EA (5 mL × 3), the organic phase was washed sequentially with saturated NH_4Cl and saturated NaCl, dried with anhydrous Na_2SO_4, filtered, concentrated under reduced pressure, and purified by PTLC (DCM : MeOH = 15 : 1) to give **compounds 21~24**.

Compound 21 (yellow solid, 35.8%). ^{1}H NMR (500 MHz, Chloroform-*d*) *δ*: 12.73 (s, 1H), 8.70 (s, 1H), 8.15 (d, 2H), 7.34 (s, 5H), 7.02 (d, 2H), 6.49 (s, 1H), 4.79 (d, *J* = 2.4 Hz, 2H), 4.75 (t, *J* = 7.9 Hz, 1H), 4.60 – 4.49 (m, 3H), 4.33 (dd, *J* = 14.9, 5.2 Hz, 1H), 4.27 – 4.17 (m, 2H), 4.09 (t, 1H), 4.04 (s, 1H), 3.98 (s, 1H), 3.90 (s, 3H), 3.78 (t, *J* = 4.8 Hz, 2H), 3.67 – 3.60 (m, 5H), 2.92 – 2.87 (m, 2H), 2.59 (t, *J* = 2.3 Hz, 1H), 2.52 (s, 3H), 2.18 – 2.04 (m, 4H), 1.68 (s, 6H). ^{13}C NMR

(126 MHz, $CDCl_3$) *δ*: 179.04, 171.38, 170.68, 170.49, 161.82, 160.57, 160.45, 156.26, 153.39, 150.38, 148.18, 138.21, 137.38, 136.66, 131.77, 130.72, 130.41, 129.45, 128.13, 122.91, 115.64, 114.03, 107.47, 105.92, 95.94, 77.38, 76.41, 71.52, 71.21, 70.42, 70.38, 70.26, 70.15, 58.41, 57.17, 56.68, 56.55, 55.44, 43.23, 35.79, 34.96, 29.70, 26.38, 25.54, 17.13, 15.94. HRMS: calculated for $C_{52}H_{60}N_4O_{12}S$ $[M + Na]^+$, 987.3821.; found, 987.3821.

Compound 22 (yellow solid, 34.5%). ^1H NMR (500 MHz, Chloroform-*d*) δ 12.72 (s, 1H), 8.78 (s, 1H), 8.16 (d, 2H), 7.47 (t, *J* = 6.1 Hz, 1H), 7.35 (s, 5H), 7.02 (d, 2H), 6.49 (s, 1H), 4.79 (d, *J* = 2.4 Hz, 2H), 4.75 (t, *J* = 8.0 Hz, 1H), 4.58 – 4.52 (m, 3H), 4.36 (dd, *J* = 15.1, 5.4 Hz, 1H), 4.25 – 4.16 (m, 2H), 4.10 – 4.05 (m, 1H), 4.04 – 3.97 (m, 2H), 3.90 (s, 3H), 3.76 (td, *J* = 4.9, 2.1 Hz, 2H), 3.70 – 3.66 (m, 4H), 3.65 – 3.58 (m, 6H), 2.92 – 2.87 (m, 3H), 2.60 (t, *J* = 2.3 Hz, 1H), 2.52 (s, 3H), 2.10 – 2.05 (m, 2H), 1.68 (s, 6H), 0.97 (s, 9H). ^{13}C NMR (126 MHz, $CDCl_3$) δ 179.09, 171.25, 170.90, 170.48, 161.82, 160.57, 160.43, 156.35, 156.24, 156.02, 153.39, 150.84, 150.28, 147.96, 138.42, 137.40, 130.41, 129.42, 128.11, 122.90, 115.64, 114.04, 107.47, 105.91, 95.94, 77.38, 76.42, 71.61, 71.11, 70.62, 70.42, 70.38, 70.30, 70.26, 70.16, 58.54, 57.12, 56.72, 56.55, 55.41, 43.18, 39.54, 36.07, 35.05, 26.39, 25.53, 17.12, 15.81. HRMS: calculated for $C_{54}H_{64}N_4O_{13}S$ $[M + Na]^+$, 1031.4083.; found, 1031.4083.

Compound 23 (yellow solid, 36.3%). ^1H NMR (500 MHz, Chloroform-*d*) *δ*: 12.73 (s, 1H), 8.71 (s, 1H), 8.14 (d, 2H), 7.43 (t, *J* = 6.0 Hz, 1H), 7.34 (s, 5H), 7.02 (d, 2H), 6.40 (s, 1H), 4.75 (t, *J* = 7.9 Hz, 1H), 4.60 – 4.50 (m, 3H), 4.33 (dd, *J* = 14.9, 5.2 Hz, 1H), 4.26 – 4.17 (m, 2H), 4.09 (t, 1H), 4.04 (s, 1H), 3.98 (s, 1H), 3.90 (d, *J* = 7.2 Hz, 6H), 3.78 (t, 2H), 3.67 – 3.60 (m, 5H), 2.90 – 2.85 (m, 2H), 2.58 – 2.53 (m, 1H), 2.52 (s, 3H), 2.17 – 2.11 (m, 1H), 2.09 – 2.05 (m, 2H), 1.67 (s, 6H), 0.95 (s, 9H). ^{13}C NMR (126 MHz, $CDCl_3$) *δ*: 179.04, 171.37, 170.71, 170.49, 162.78, 161.75, 160.66, 156.10, 153.29, 150.37, 148.06, 144.72, 138.26, 137.27, 131.84, 130.65, 130.37, 129.44, 128.14, 123.02, 115.64, 114.00, 106.80, 105.33, 94.82, 71.52, 71.22, 70.42, 70.38, 70.26, 70.15, 58.42, 57.18, 56.69, 56.12, 55.44, 43.23, 35.81, 26.39, 25.56, 17.00, 15.88. HRMS: calculated for $C_{50}H_{60}N_4O_{12}S$ $[M + Na]^+$, 963.3821.; found, 963.3821.

Compound 24 (yellow solid, 35.9%). ^1H NMR (500 MHz, Chloroform-*d*) δ 12.71 (s, 1H), 8.15 (d, 2H), 7.49 (t, *J* = 5.9 Hz, 1H), 7.34 (s, 5H), 7.02 (d, 2H), 6.40 (s, 1H), 4.76 (t, *J* = 8.0 Hz, 1H), 4.58 – 4.51 (m, 3H), 4.36 (dd, *J* = 15.1, 5.4 Hz, 1H), 4.19 (td, *J* = 5.1, 3.0 Hz, 2H), 4.05 (dd, *J* = 29.6, 13.9 Hz, 2H), 3.90 (d, *J* = 4.9 Hz, 6H), 3.78 – 3.74 (m, 2H), 3.70 – 3.66 (m, 4H), 3.66 – 3.60 (m, 6H), 2.90 – 2.85 (m, 2H), 2.53 (s, 3H), 2.09 – 2.05 (m, 2H), 1.67 (s, 6H), 0.97 (s, 9H). ^{13}C NMR (126 MHz, $CDCl_3$) δ 179.08, 171.27, 170.92, 170.50, 162.79, 161.76, 160.65, 156.09, 153.30, 147.80, 138.53, 137.28, 130.36, 129.42, 128.13, 122.99, 114.02, 106.81, 105.31, 94.82, 88.78, 71.61, 71.11, 70.61, 70.41, 70.36, 70.28, 70.25, 70.18, 58.55, 57.15, 56.71, 56.12, 55.41, 43.17, 39.40,

36.10, 35.05, 26.39, 25.55, 16.99, 15.74. HRMS: calculated for $C_{52}H_{64}N_4O_{13}S$ $[M + Na]^+$, 1007.4083.; found, 1007.4083.

1H and 13C 1D NMR spectra for compounds 1a/b and compounds 3-24

Supplementary Figure 3: 1H and 13C spectra of compounds 1a

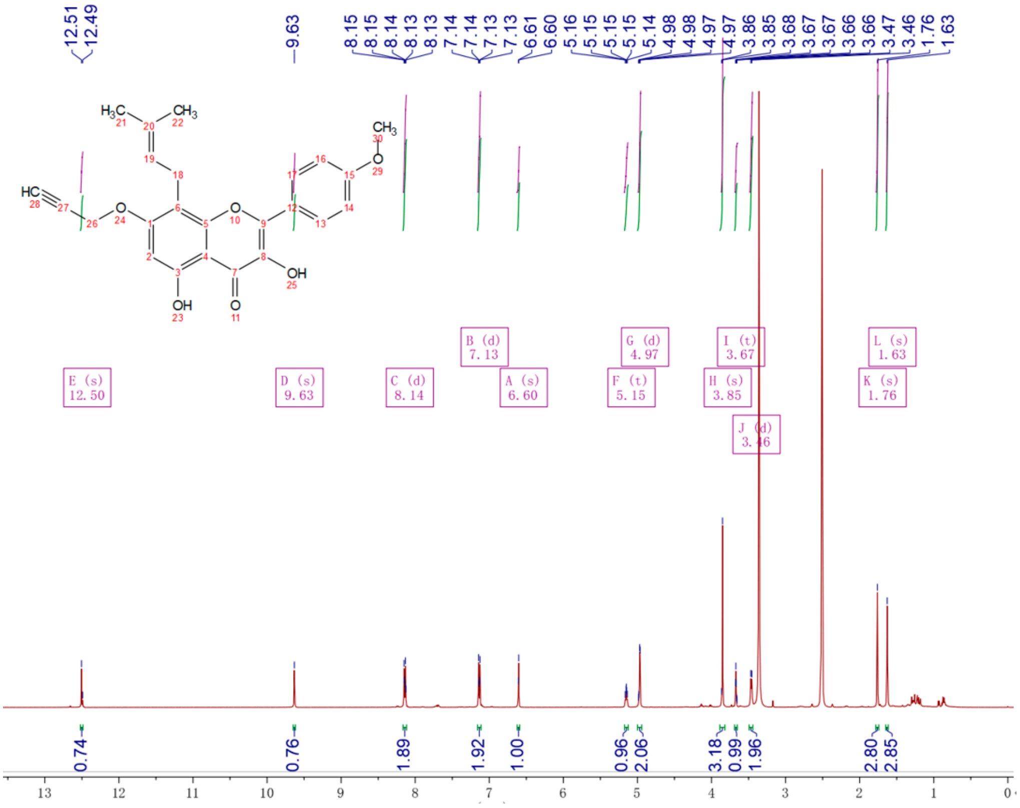

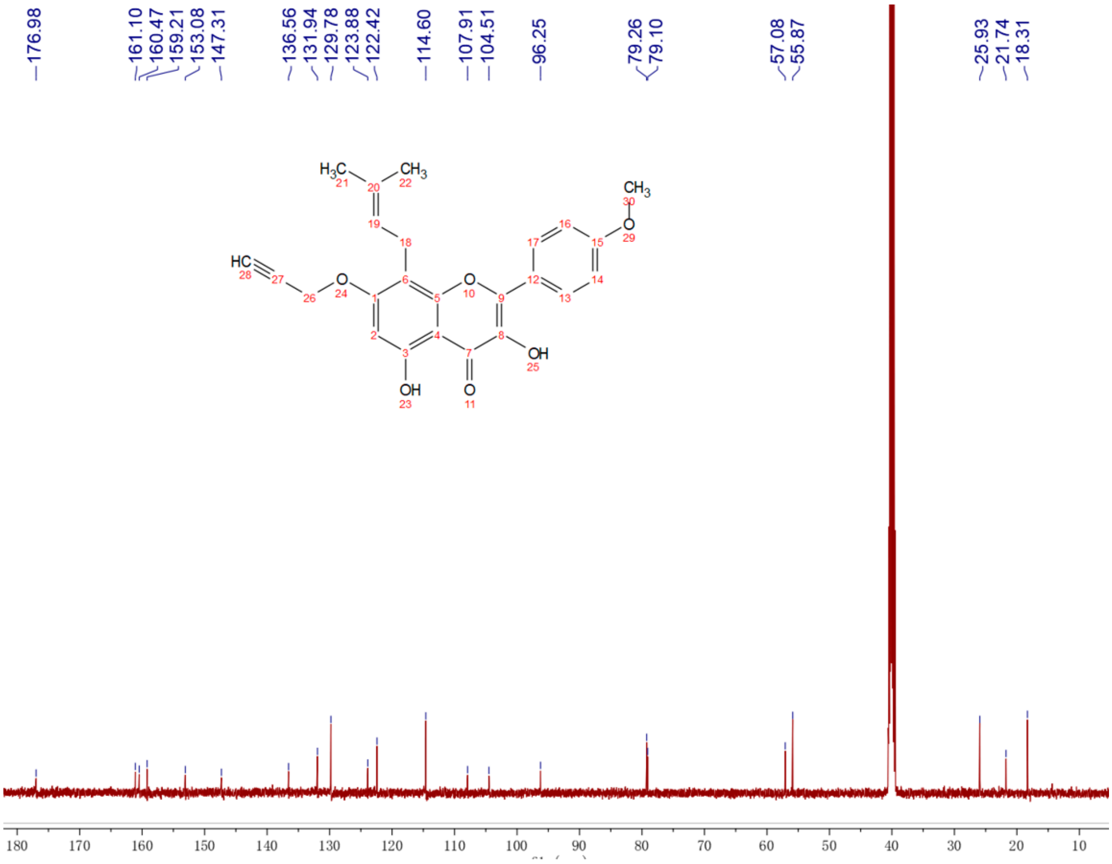

Supplementary Figure 4: 1H and 13C spectra of compound 1b

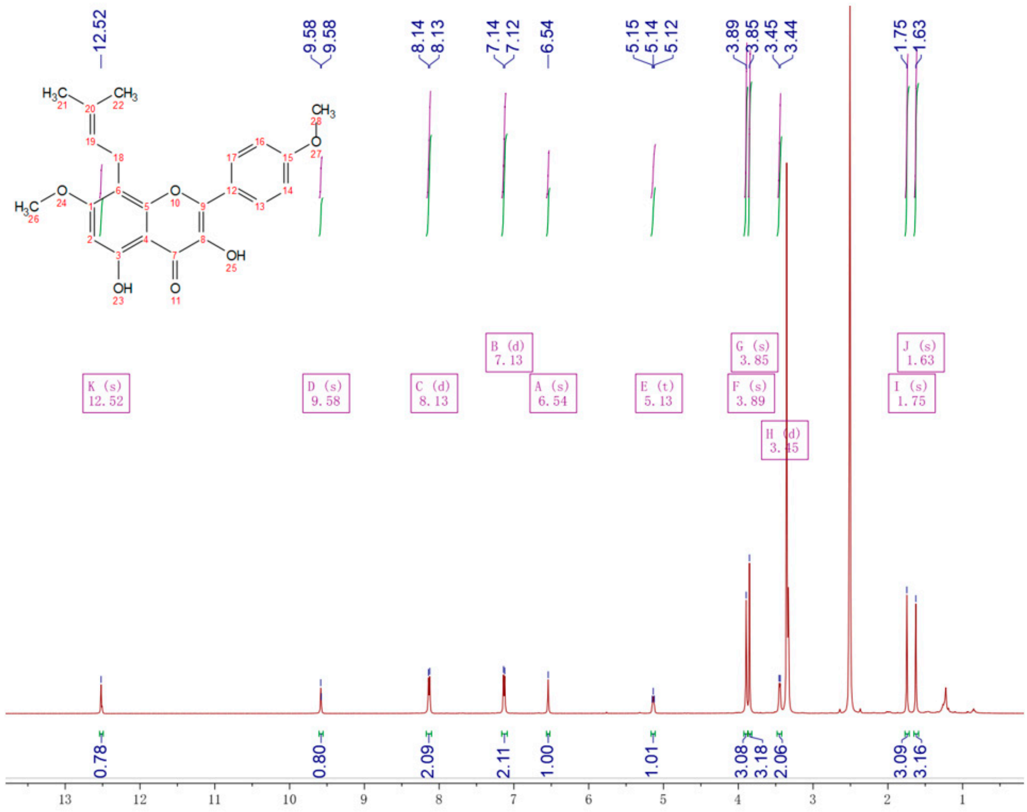

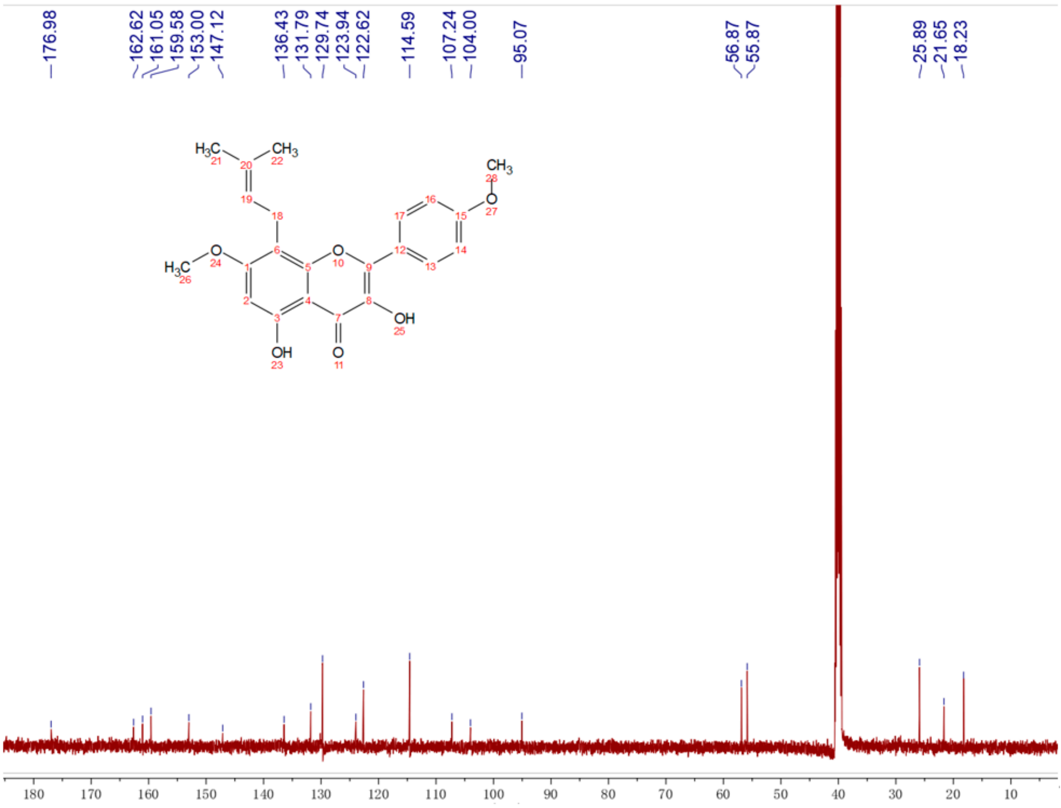

Supplementary Figure 5: 1H and 13C spectra of compounds 3

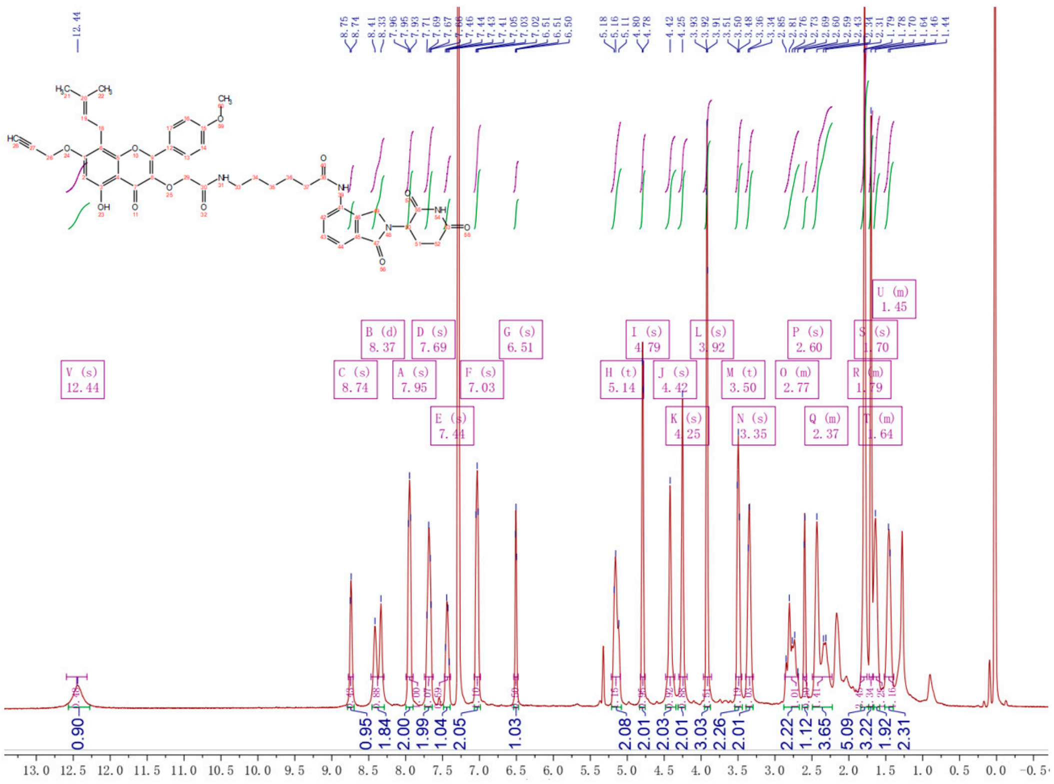

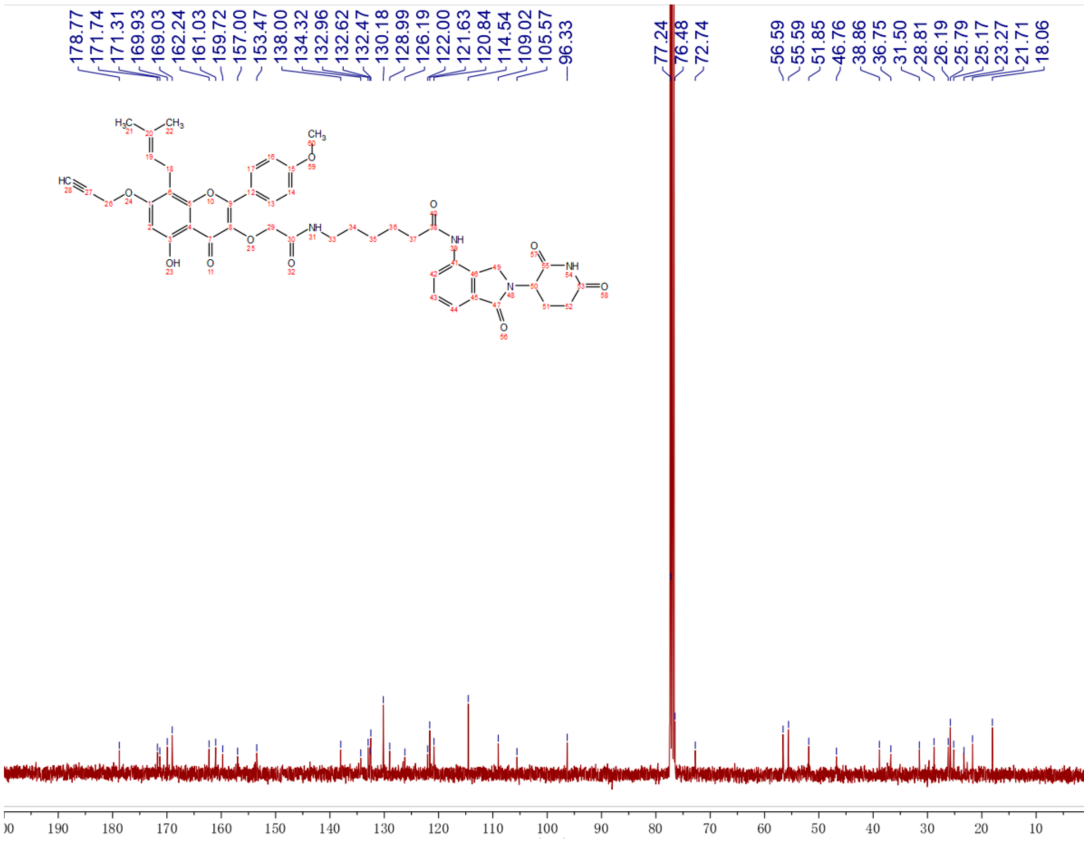

Supplementary Figure 6: 1H and 13C spectra of compounds 4

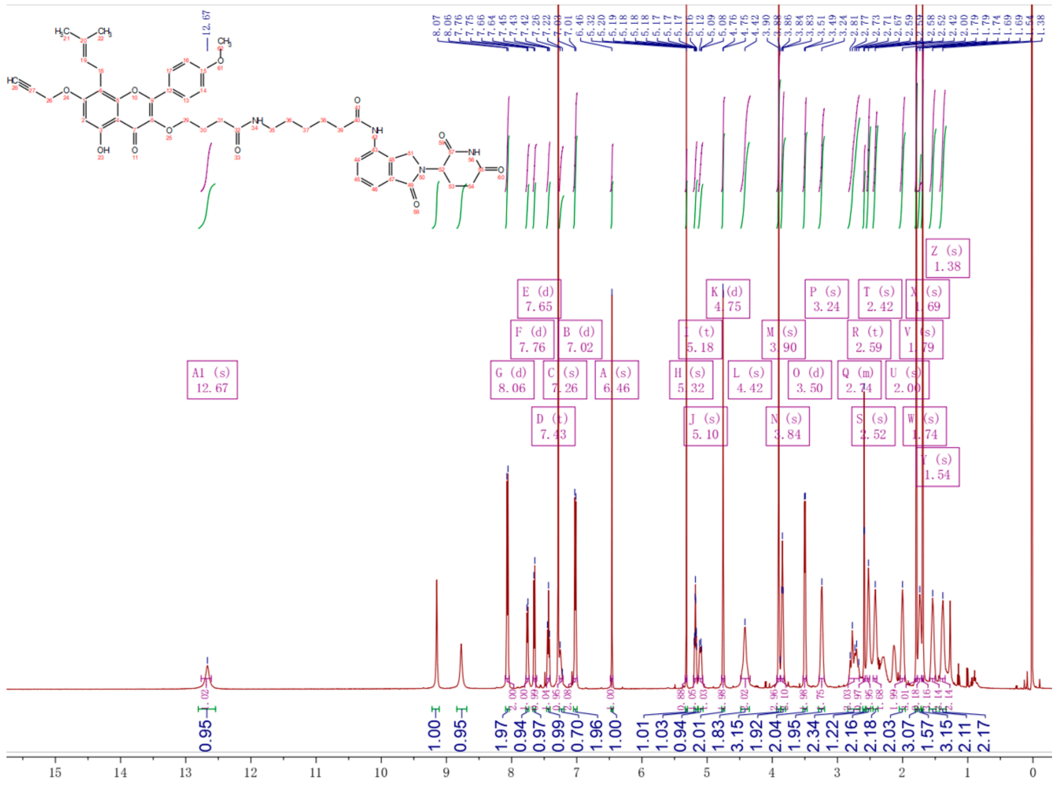

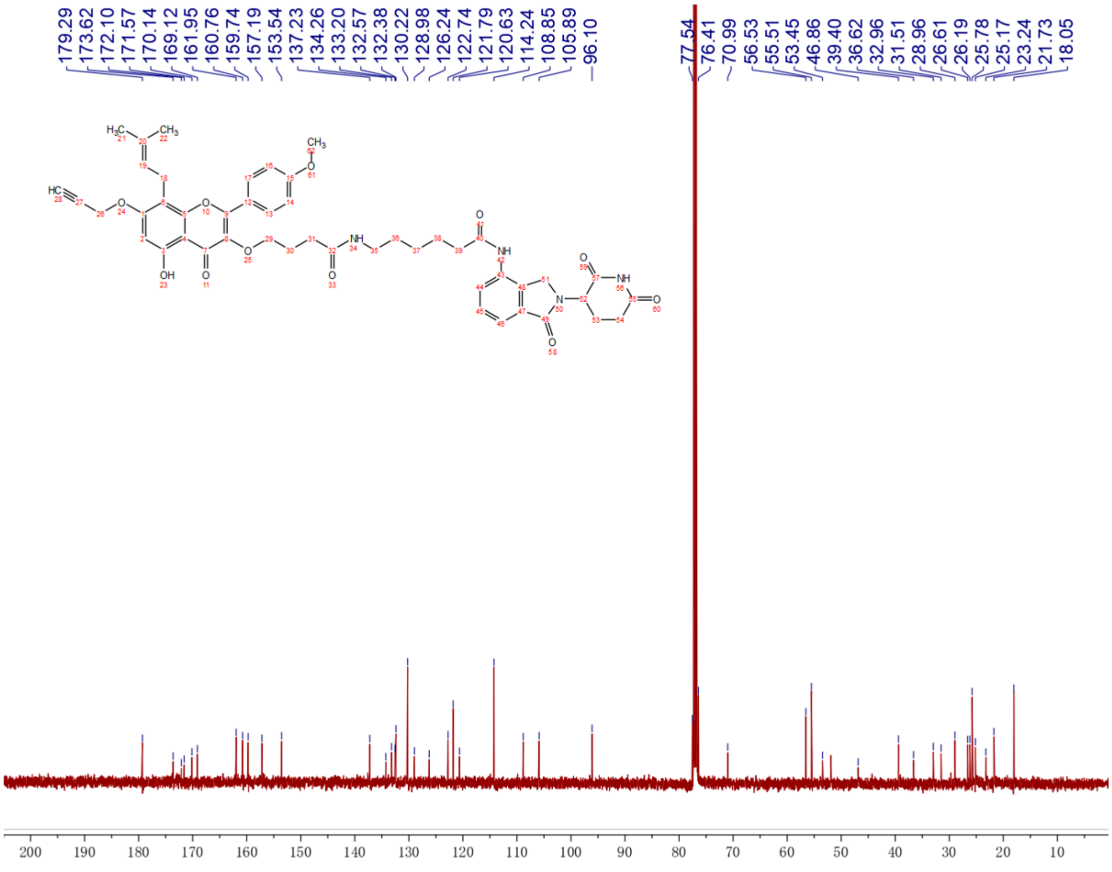

Supplementary Figure 7: 1H and 13C spectra of compounds 5

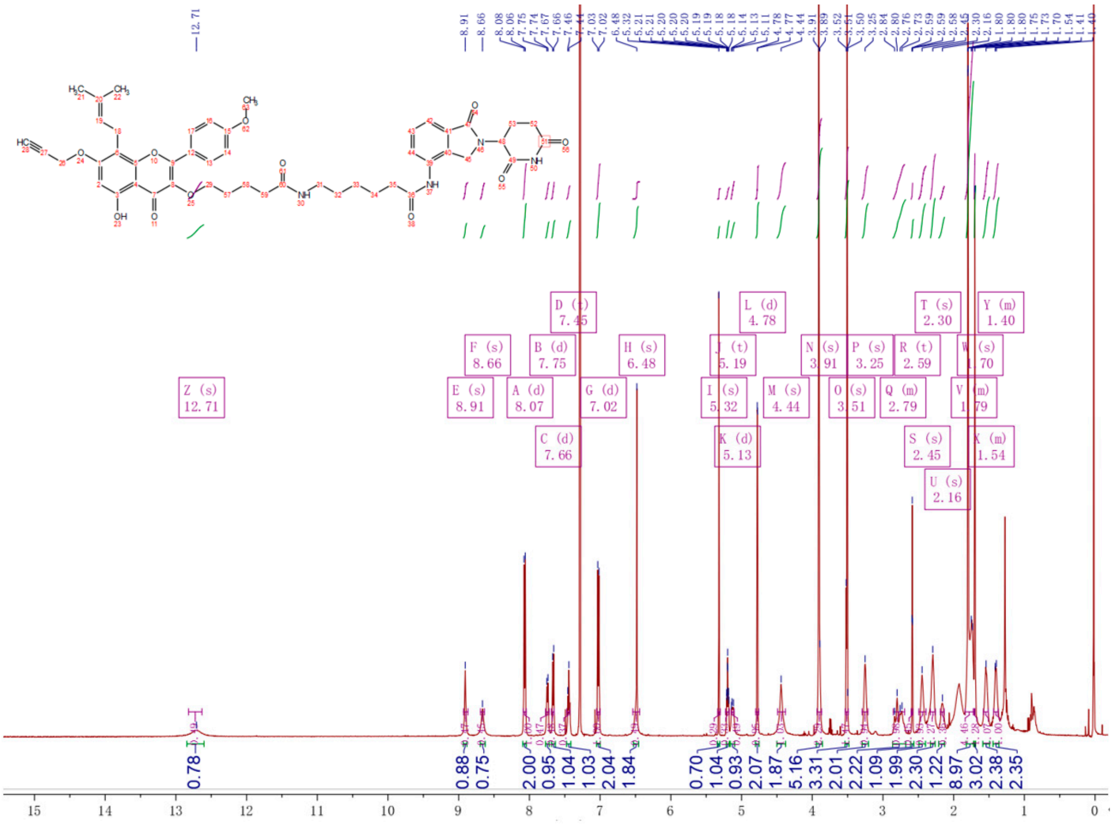

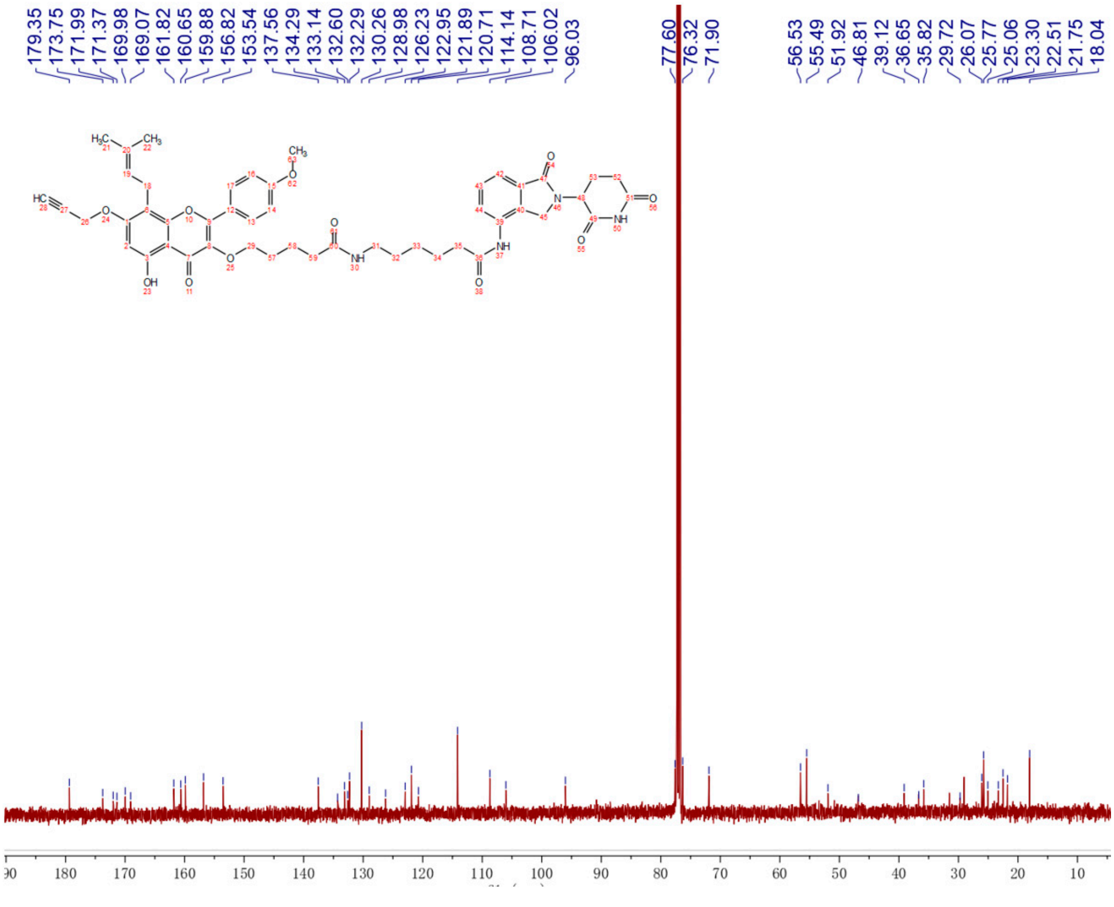

Supplementary Figure 8: 1H and 13C spectra of compounds 6

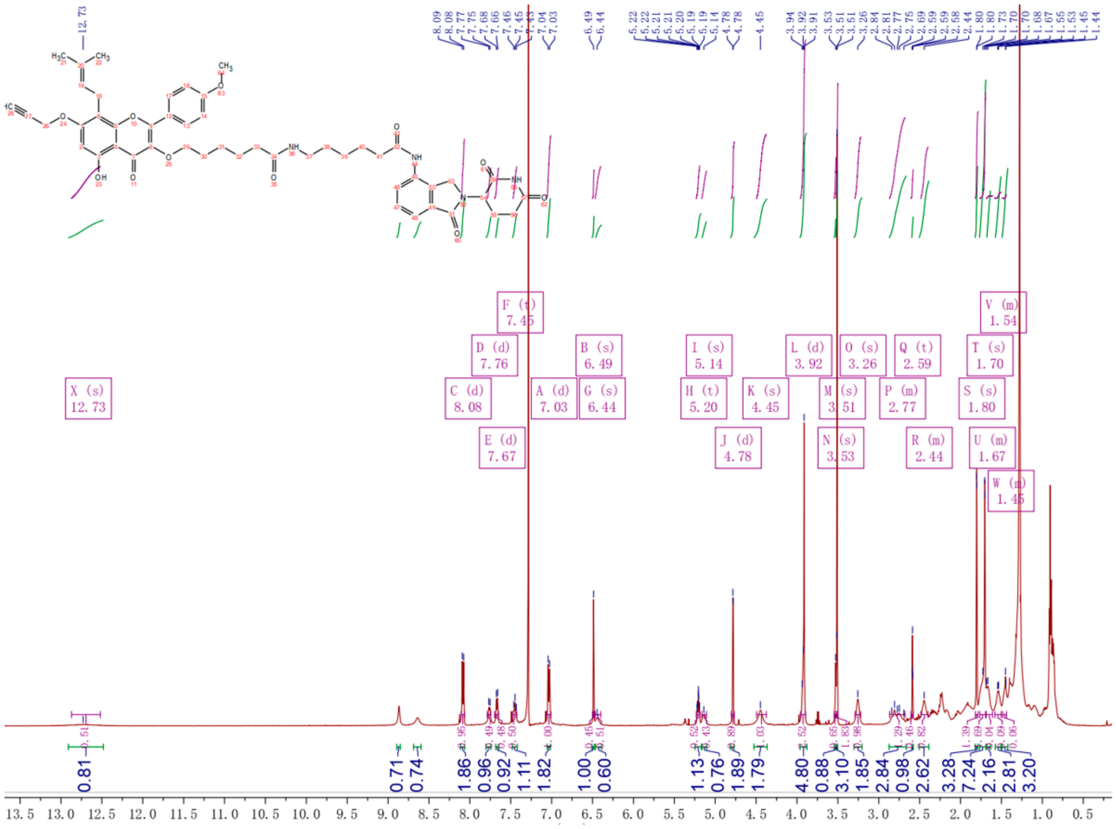

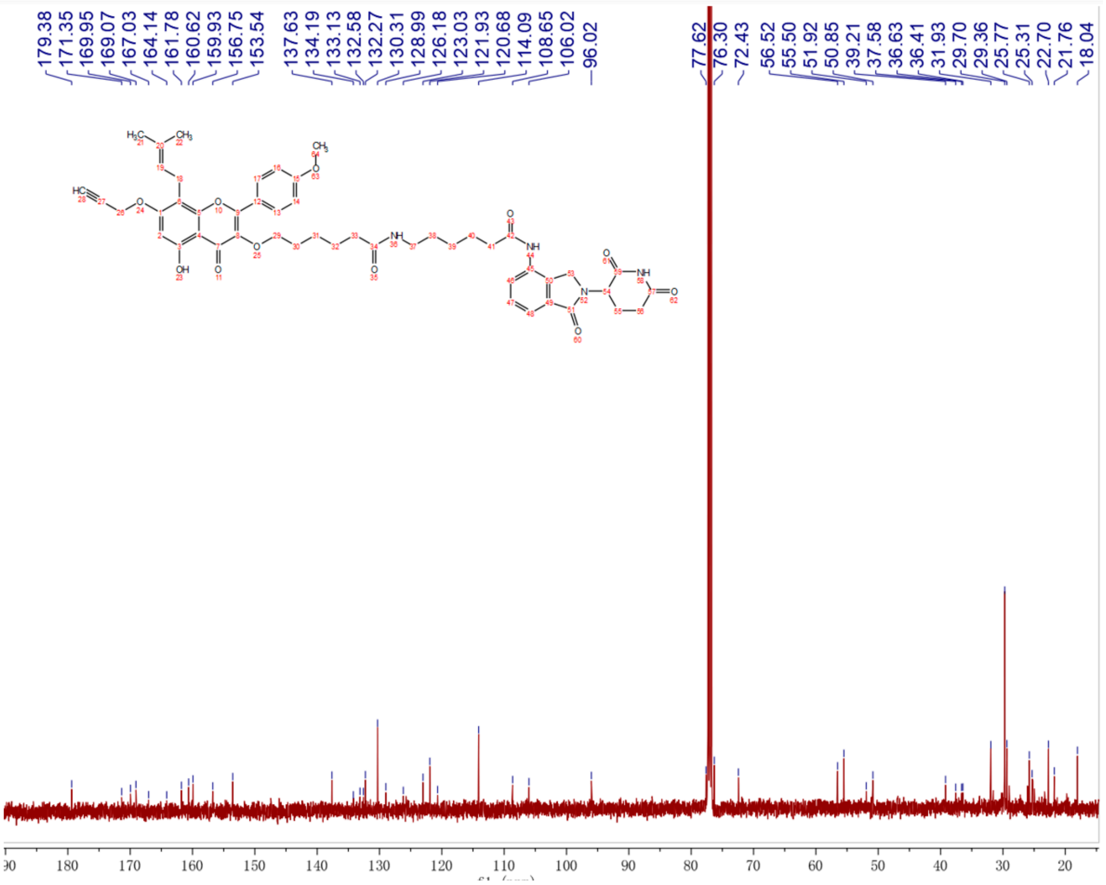

Supplementary Figure 9: 1H and 13C spectra of compounds 7

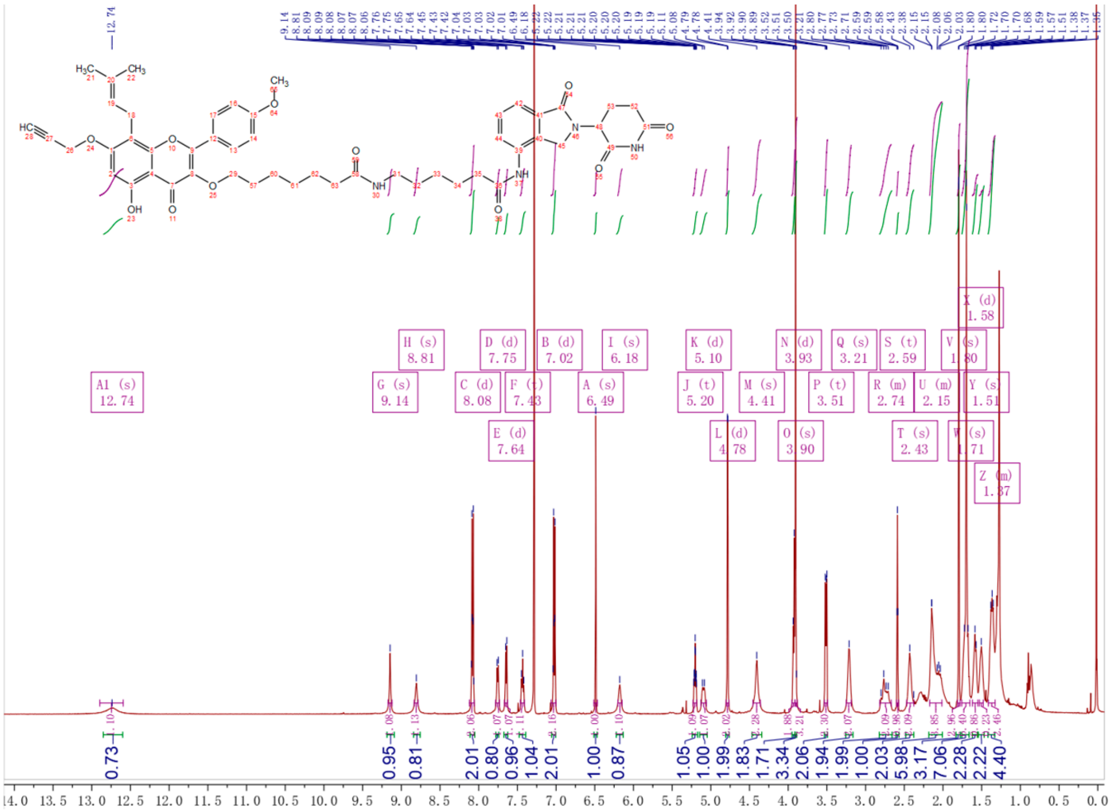

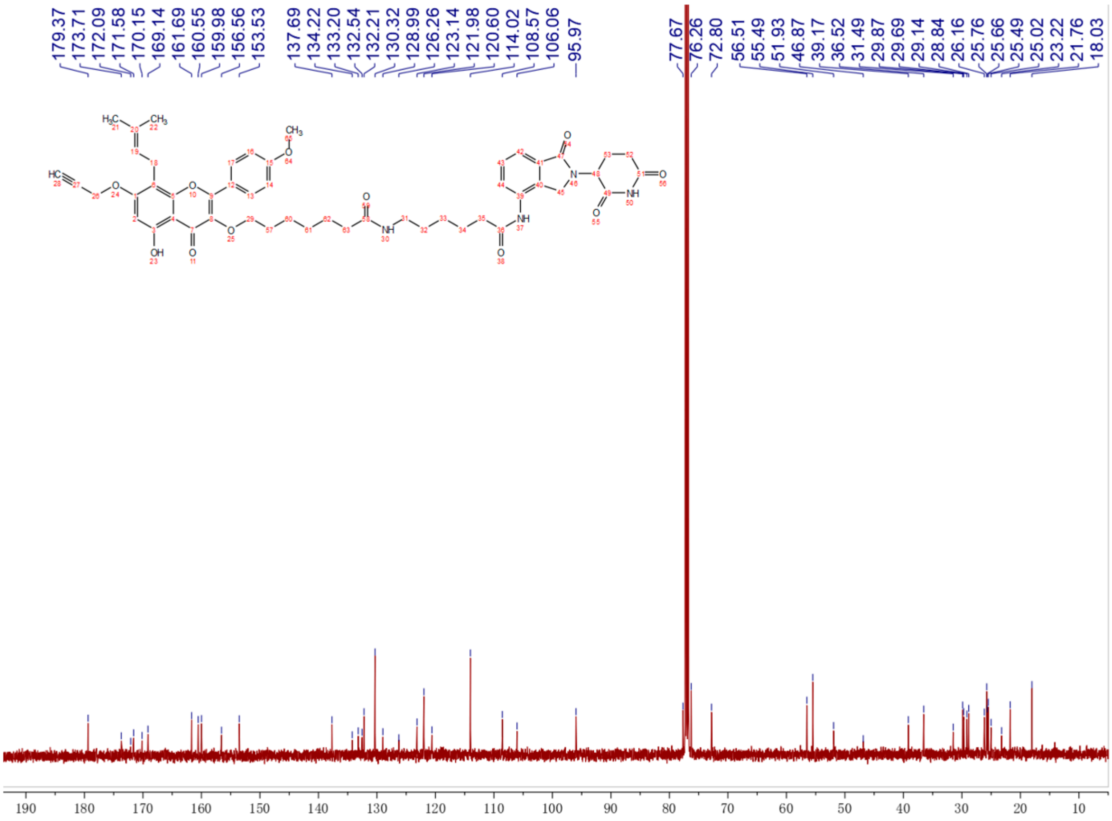

Supplementary Figure 10: 1H and 13C spectra of compounds 8

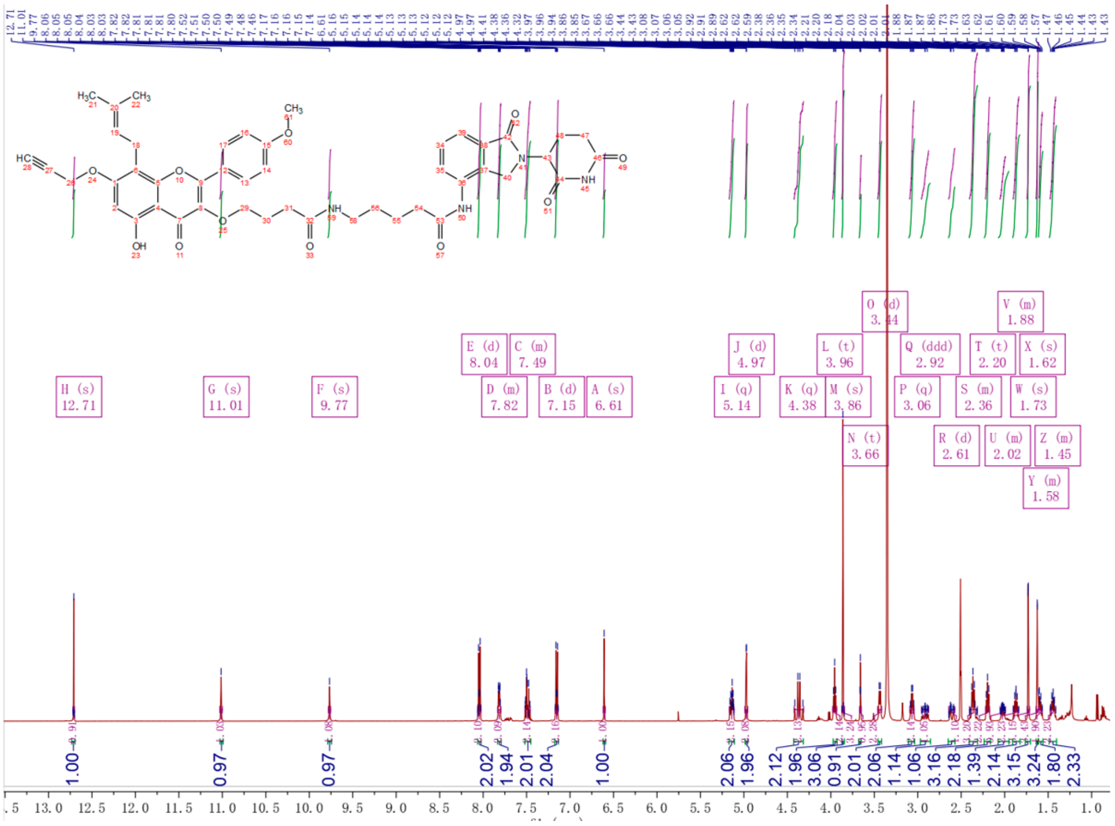

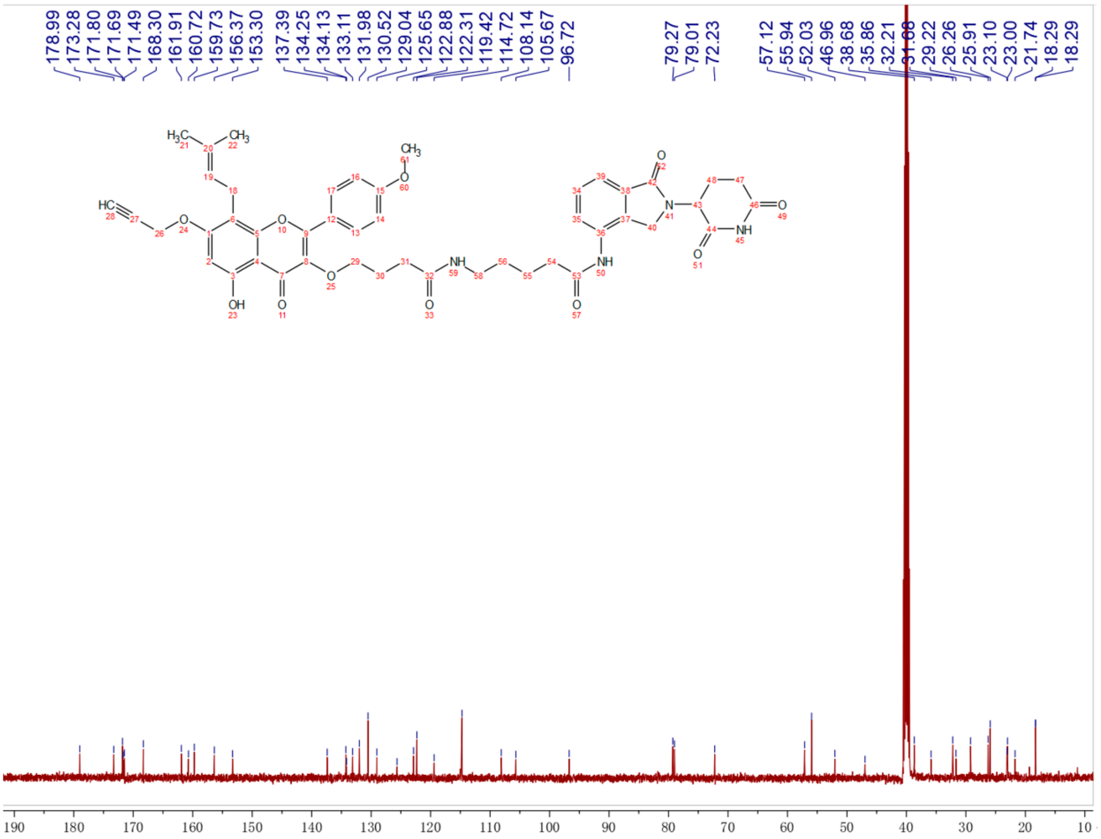

Supplementary Figure 11: 1H and 13C spectra of compounds 9

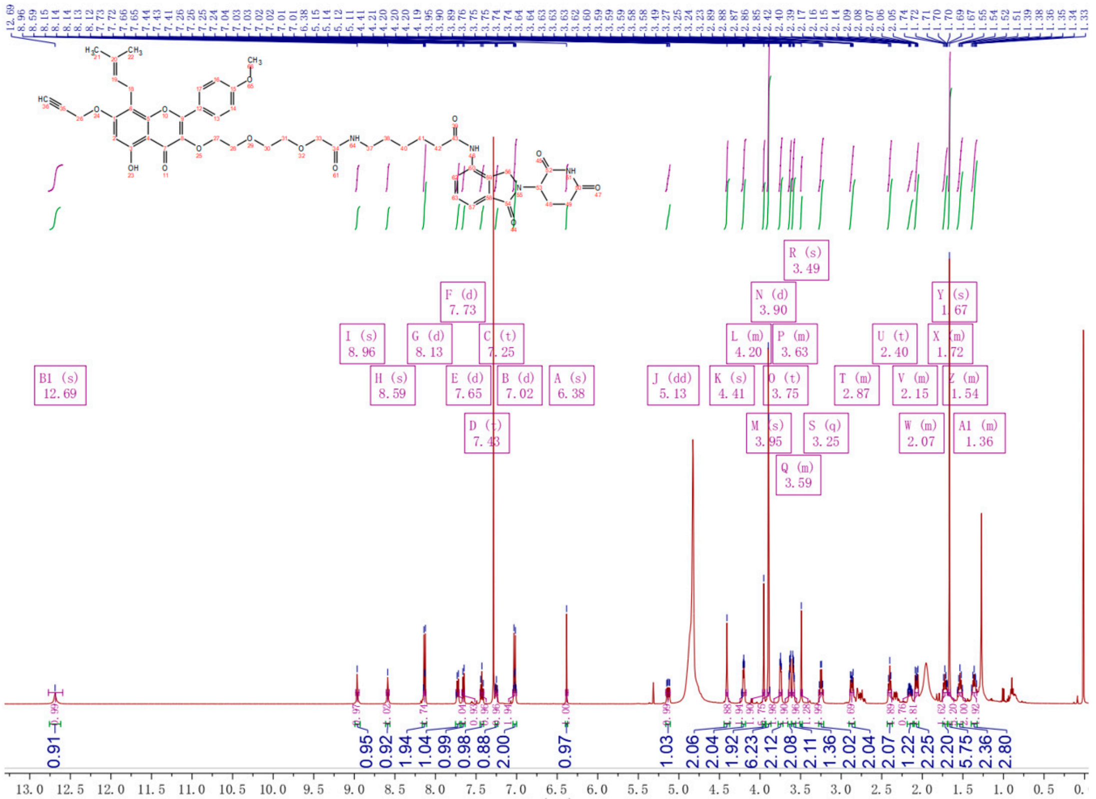

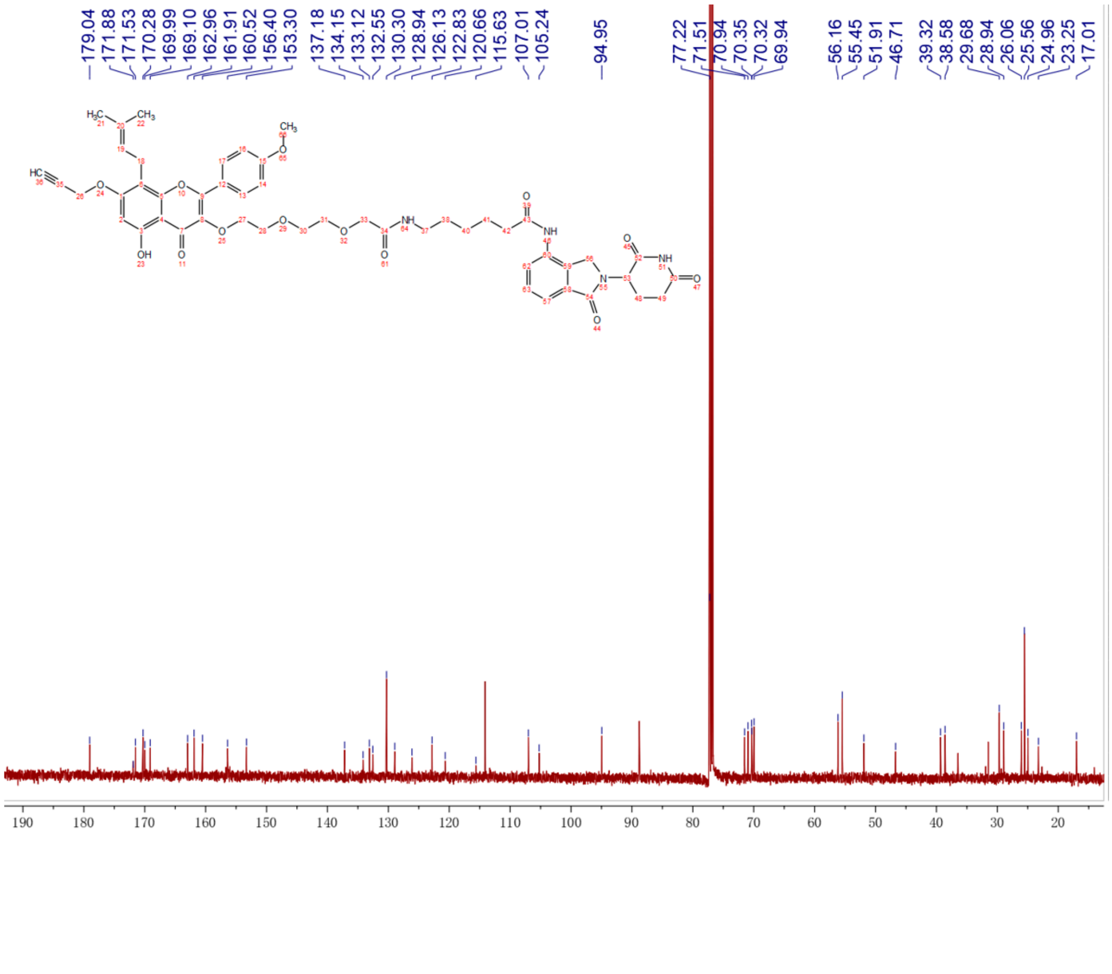

Supplementary Figure 12: 1H and 13C spectra of compounds 10

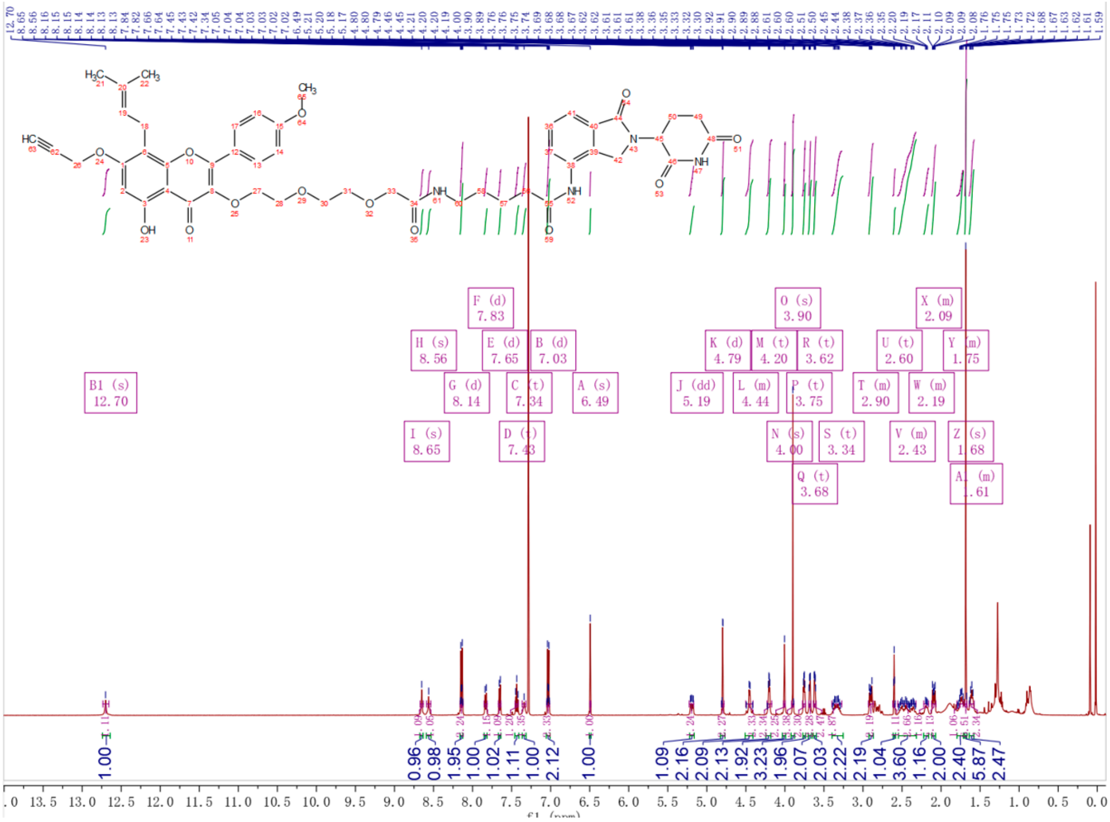

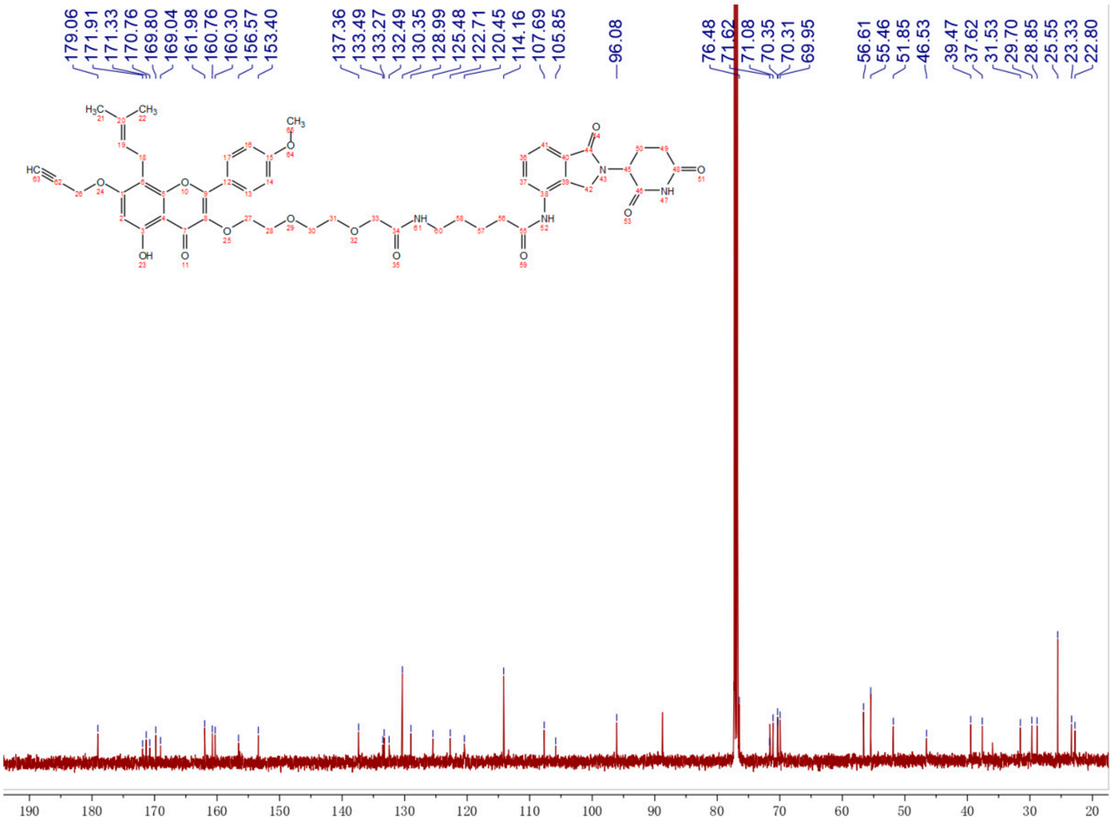

Supplementary Figure 13: 1H and 13C spectra of compounds 11

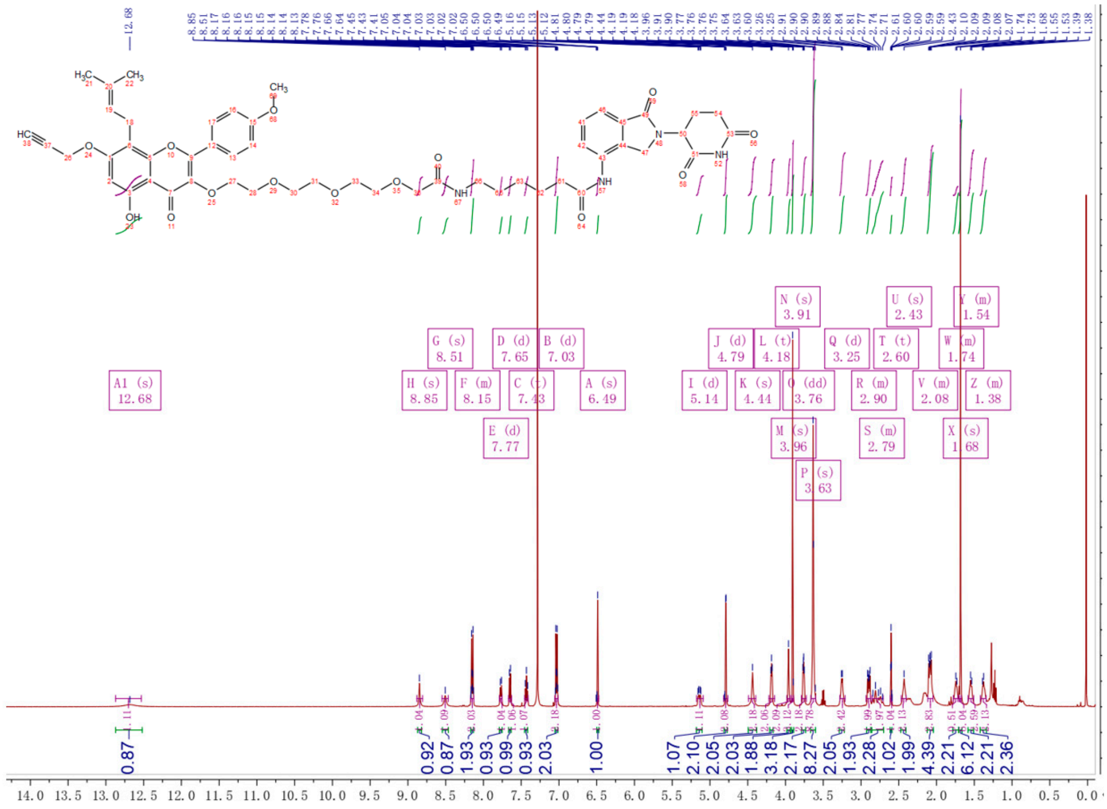

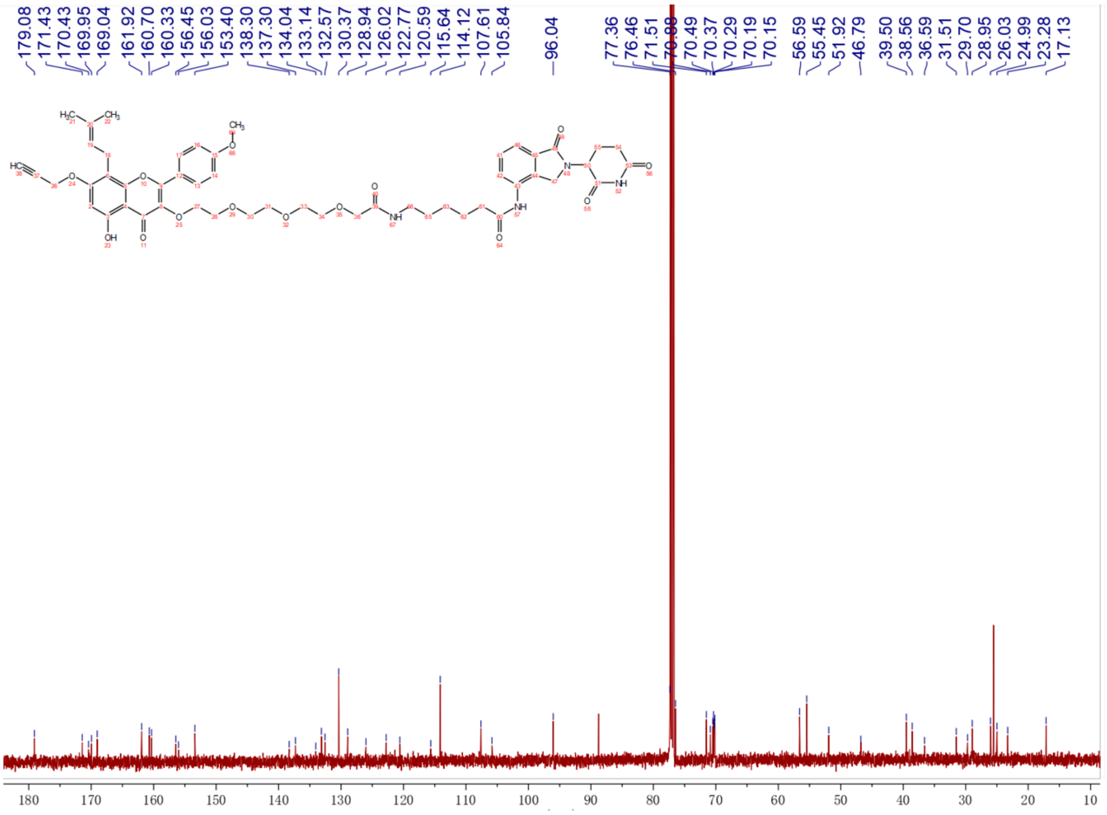

Supplementary Figure 14: 1H and 13C spectra of compounds 12

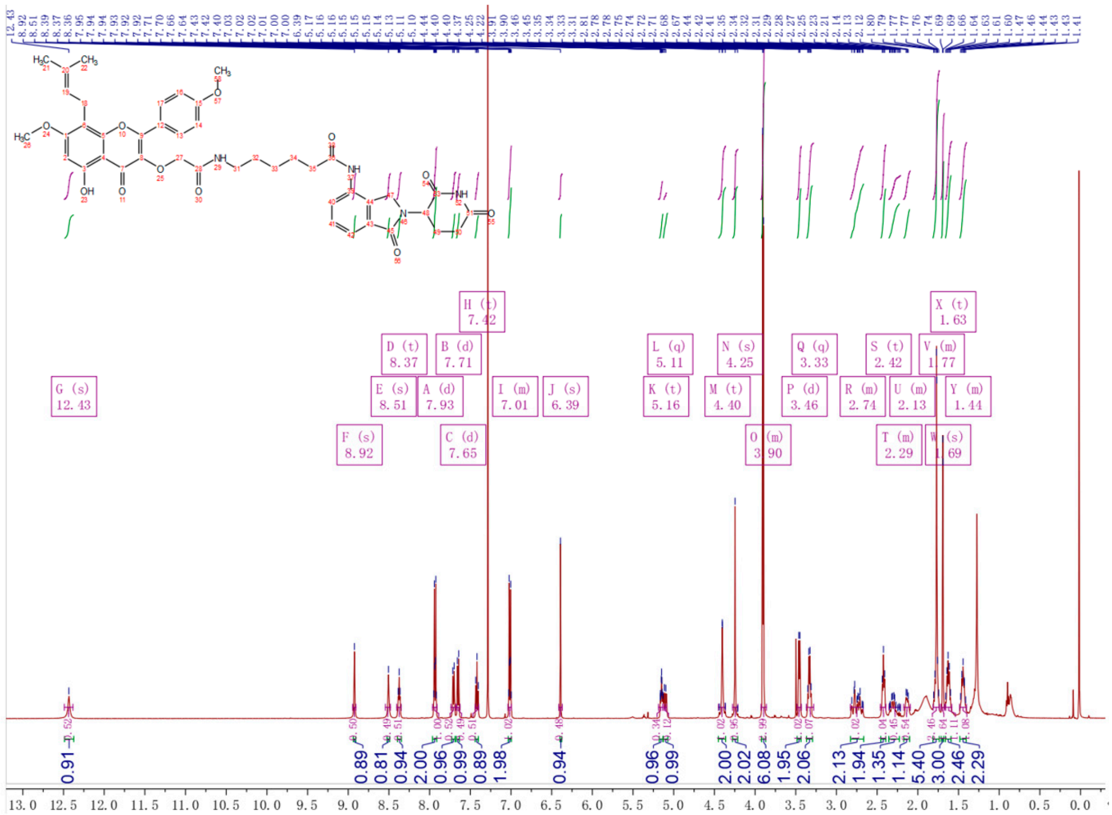

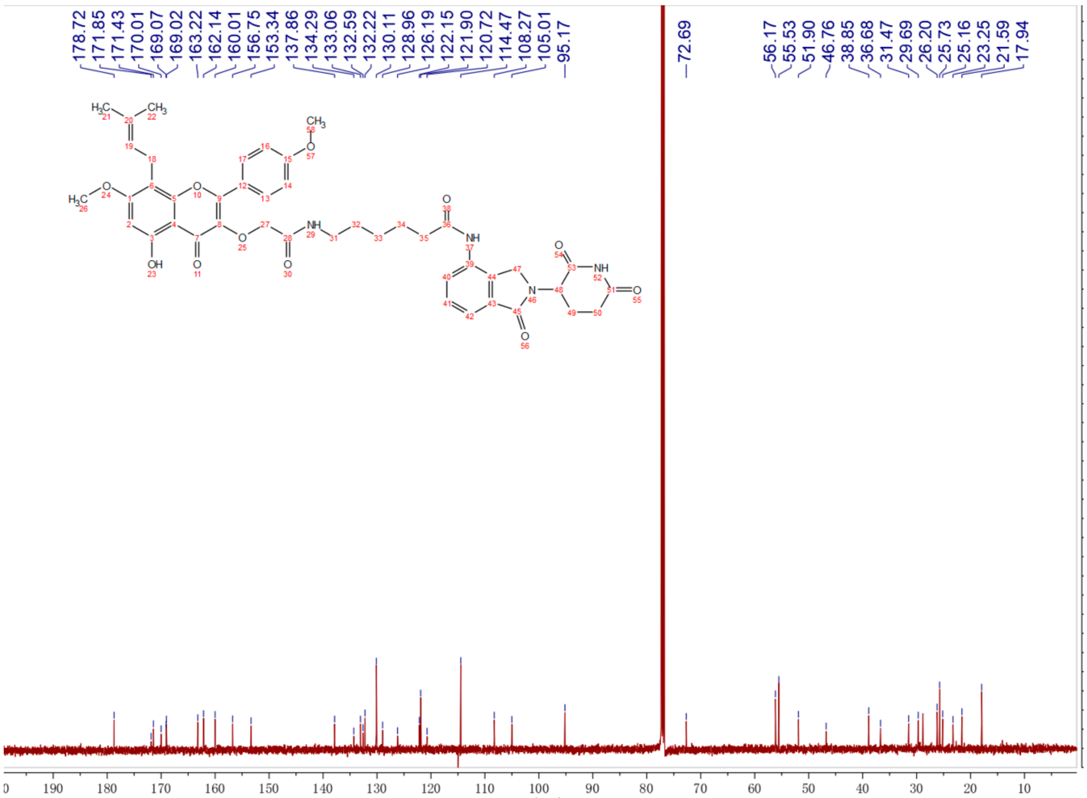

Supplementary Figure 15: 1H and 13C spectra of compounds 13

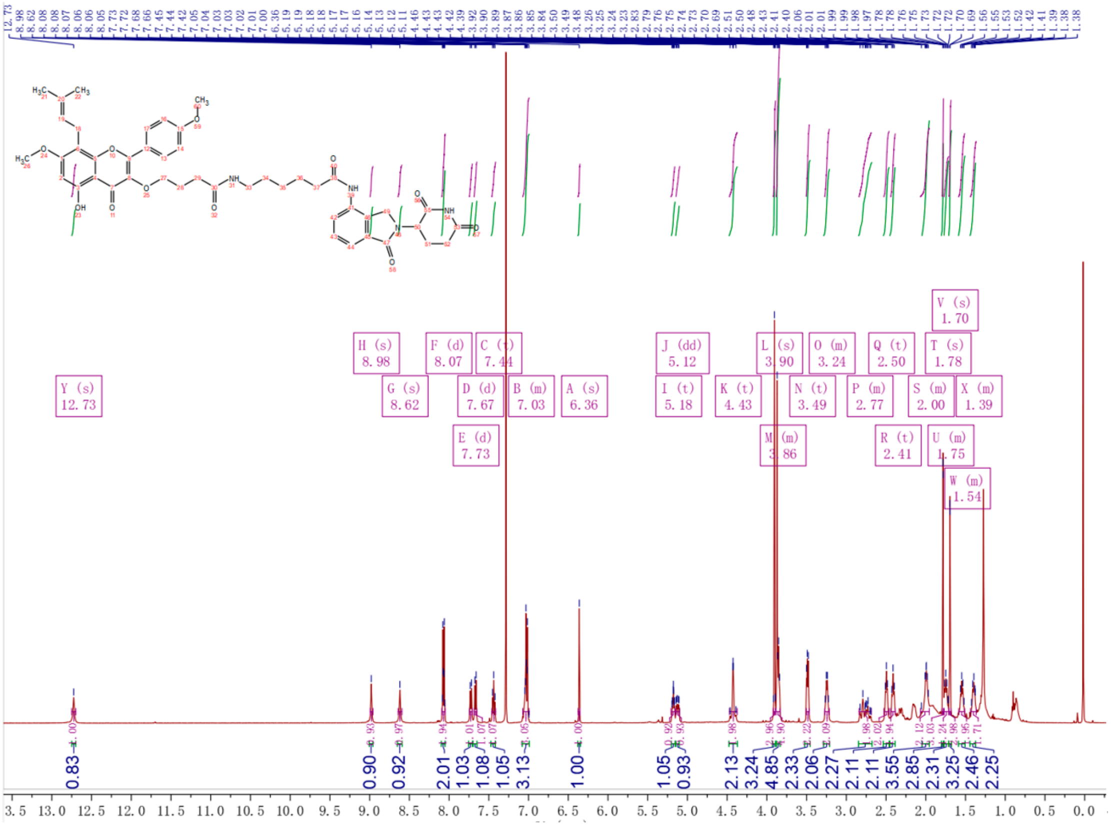

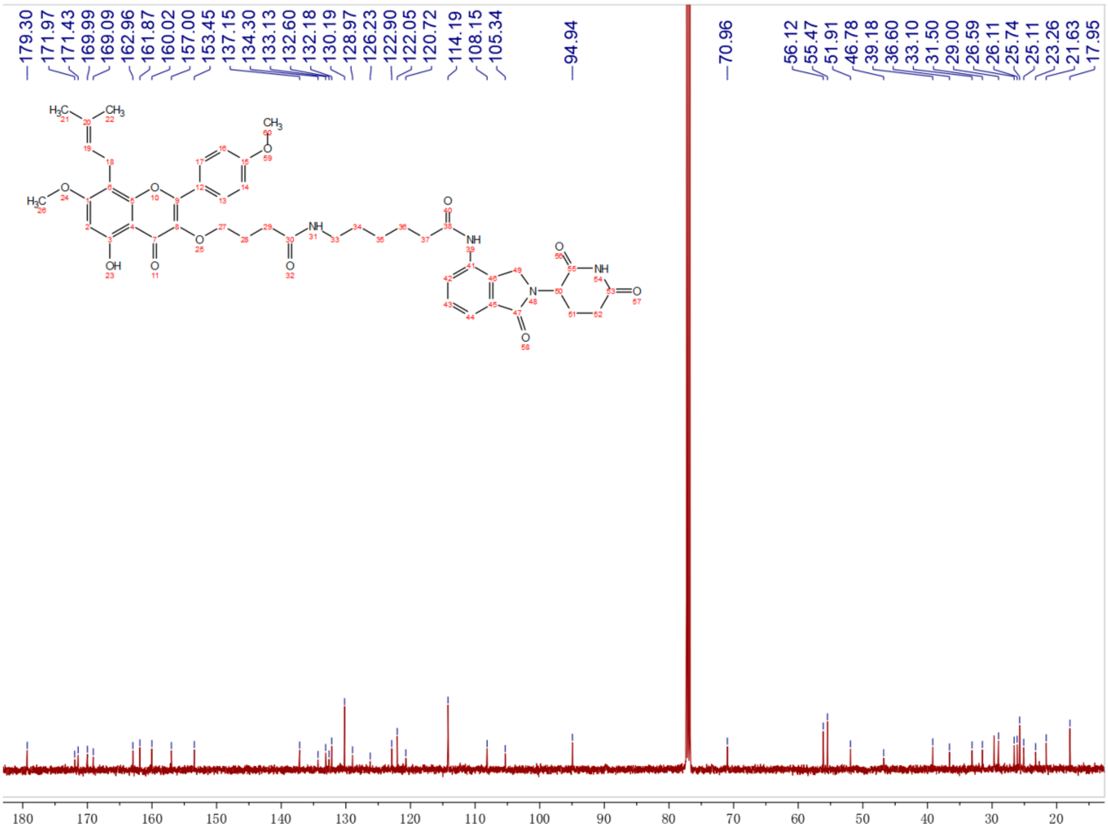

Supplementary Figure 16: 1H and 13C spectra of compounds 14

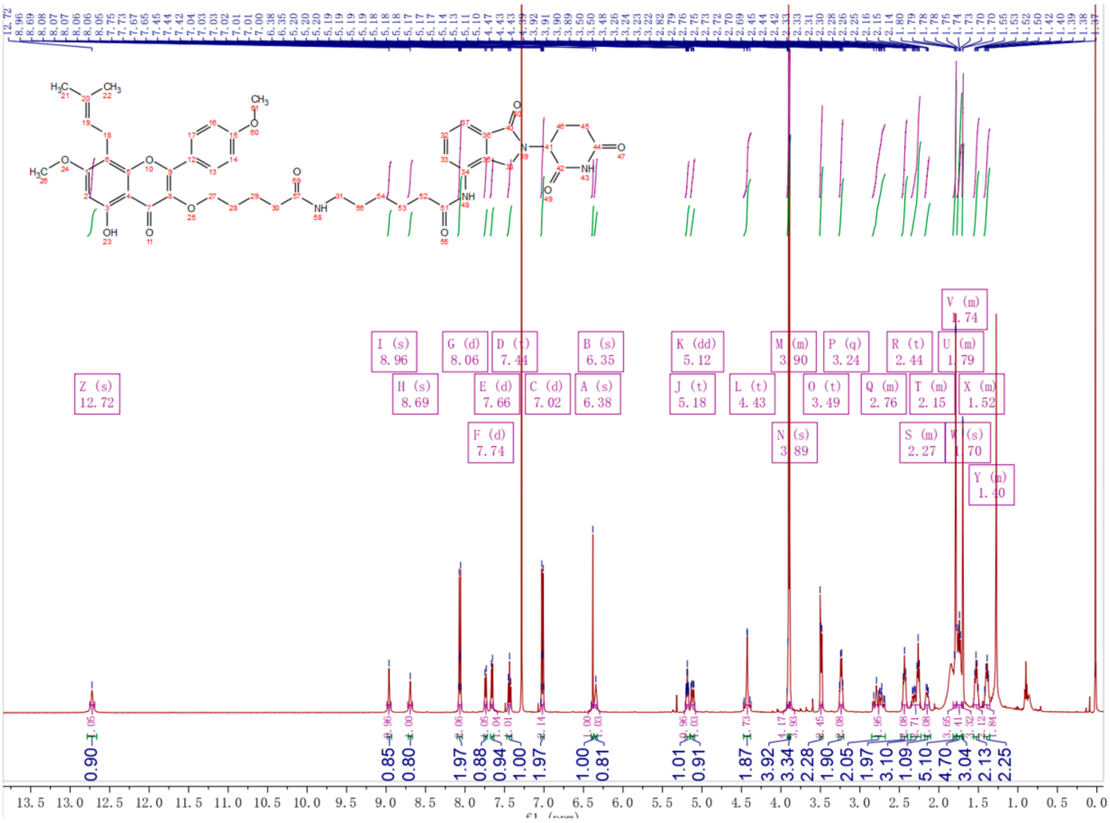

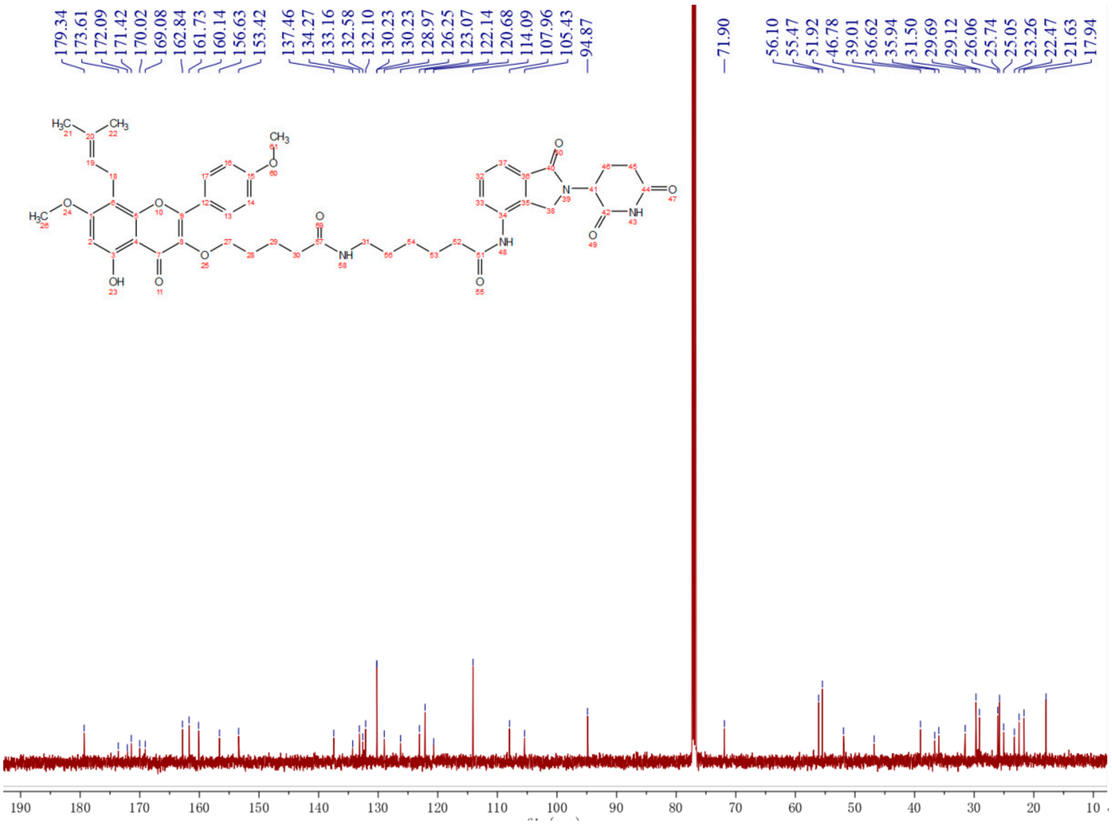

Supplementary Figure 17: 1H and 13C spectra of compounds 15

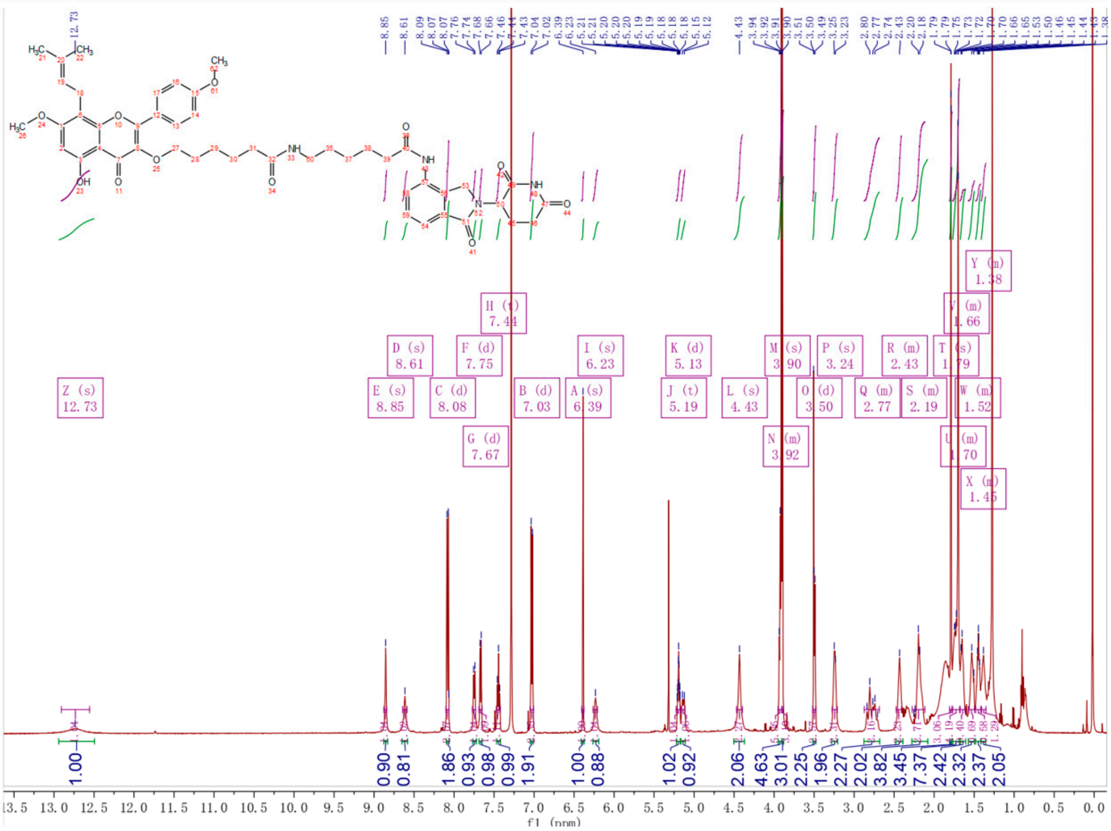

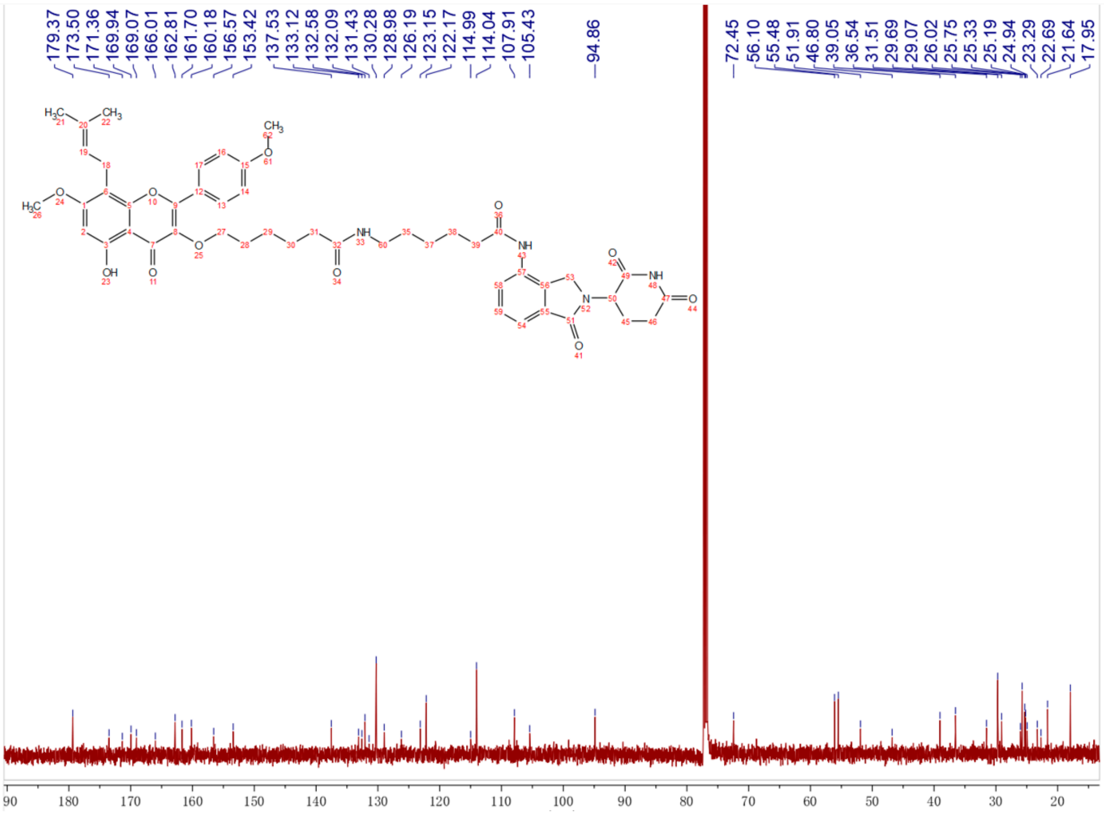

Supplementary Figure 18: 1H and 13C spectra of compounds 16

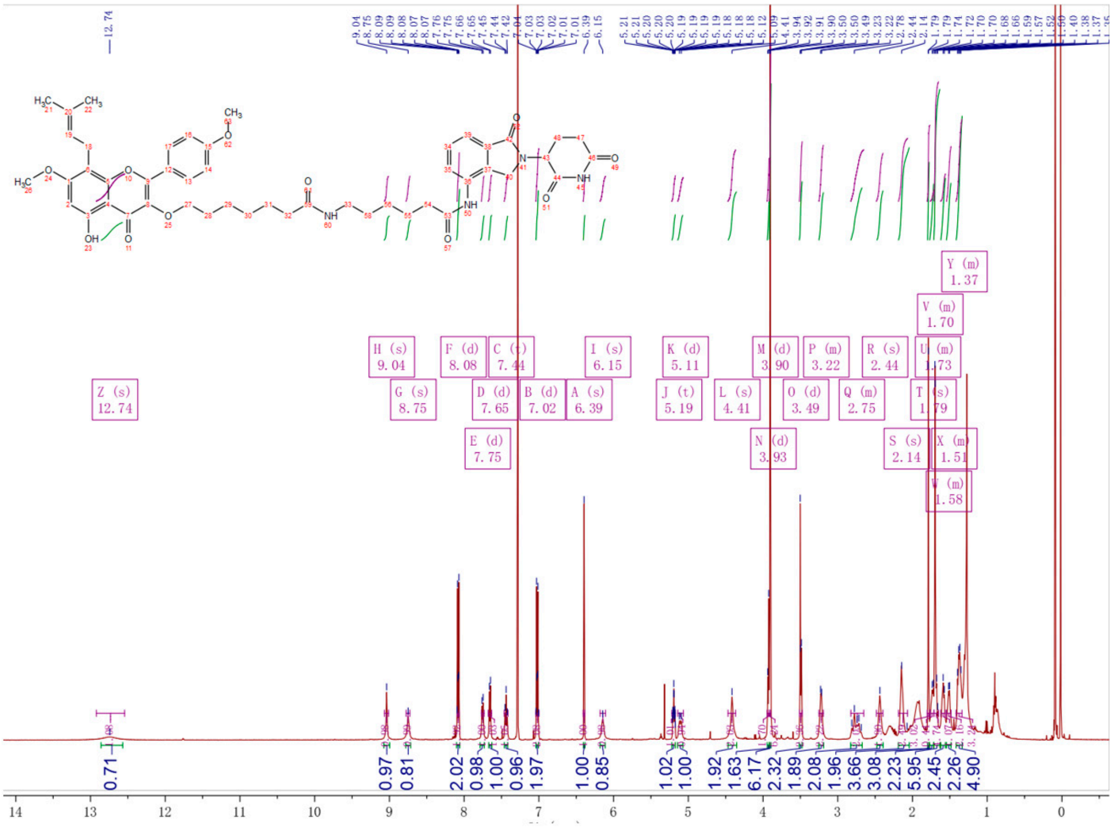

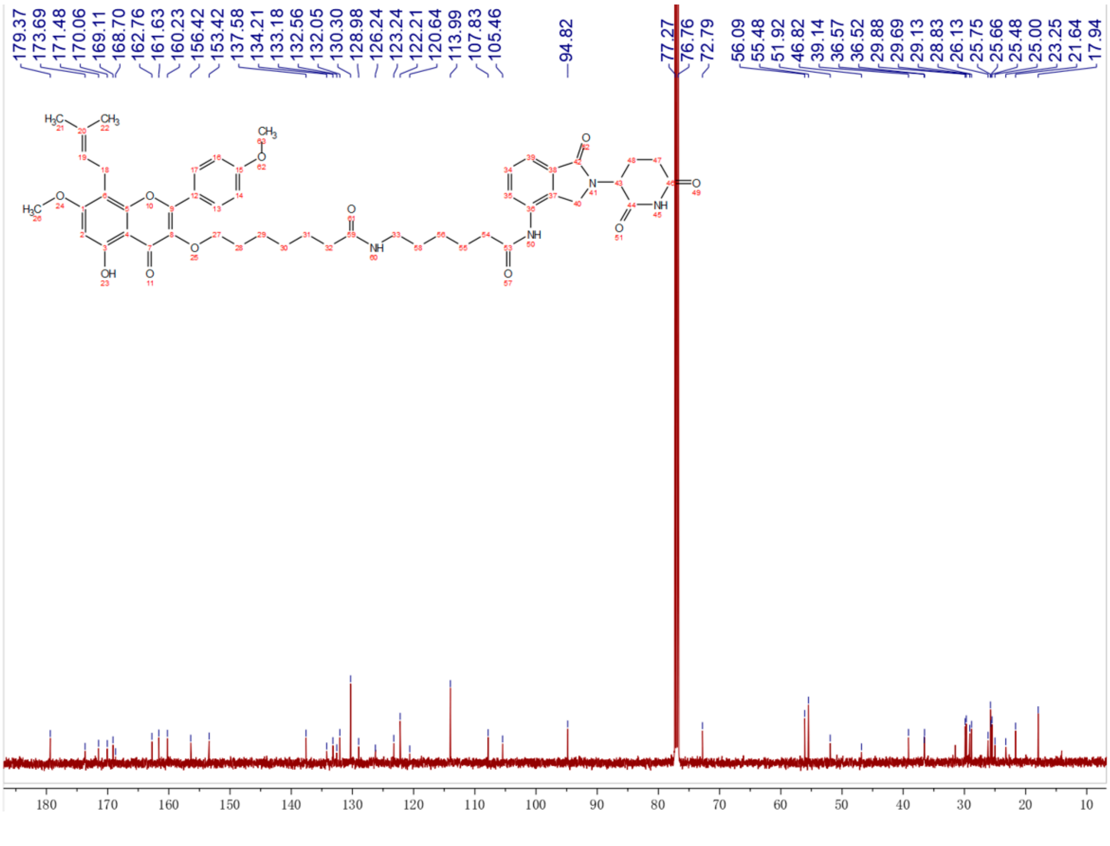

Supplementary Figure 19: 1H and 13C spectra of compounds 17

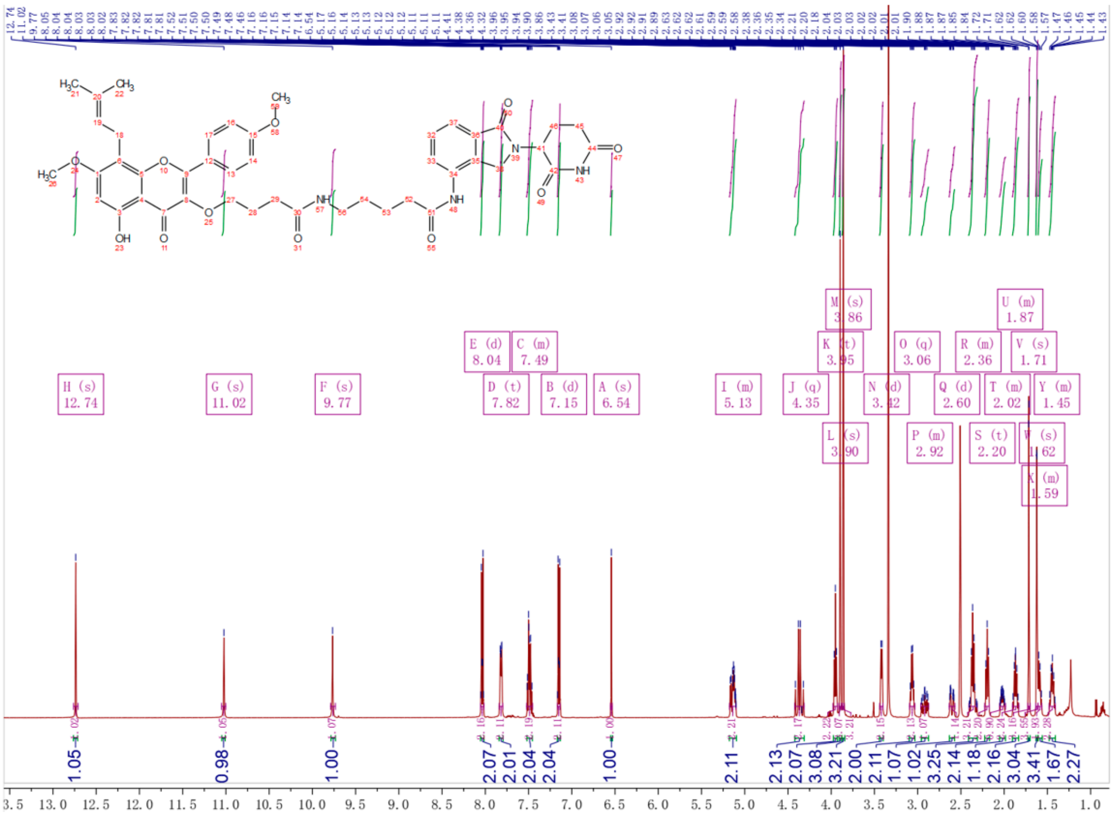

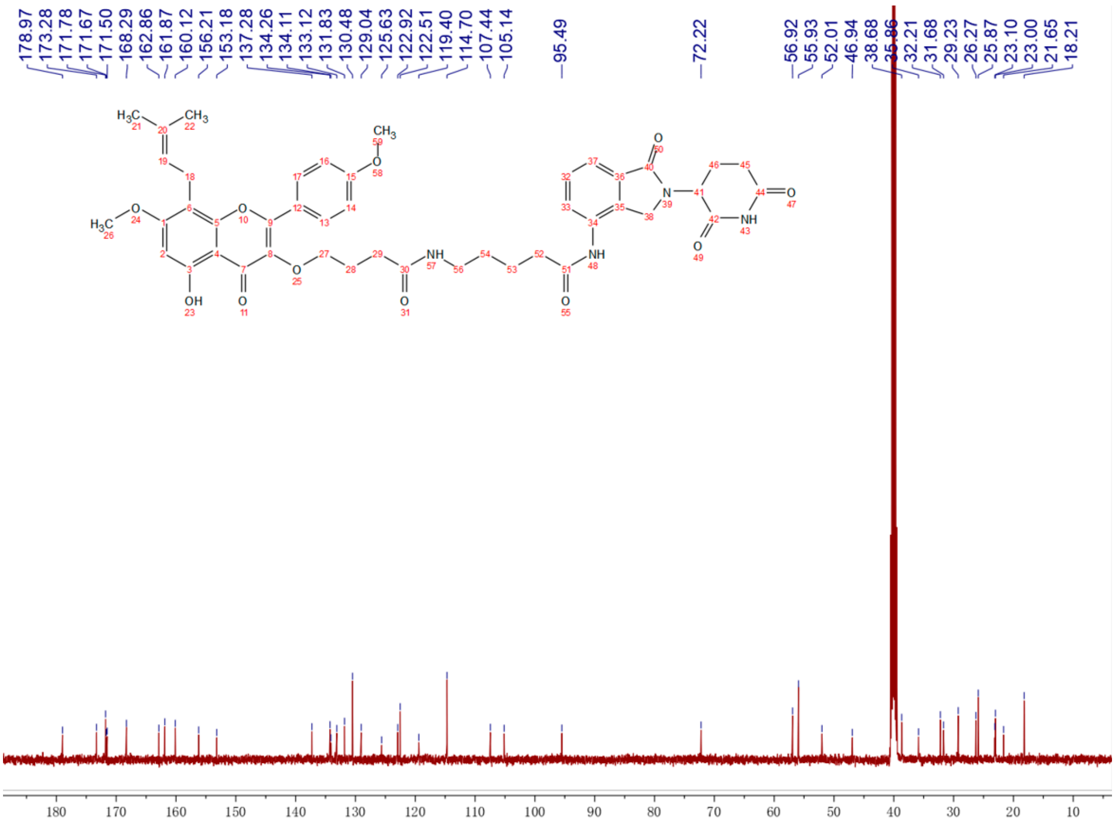

Supplementary Figure 20: 1H and 13C spectra of compounds 18

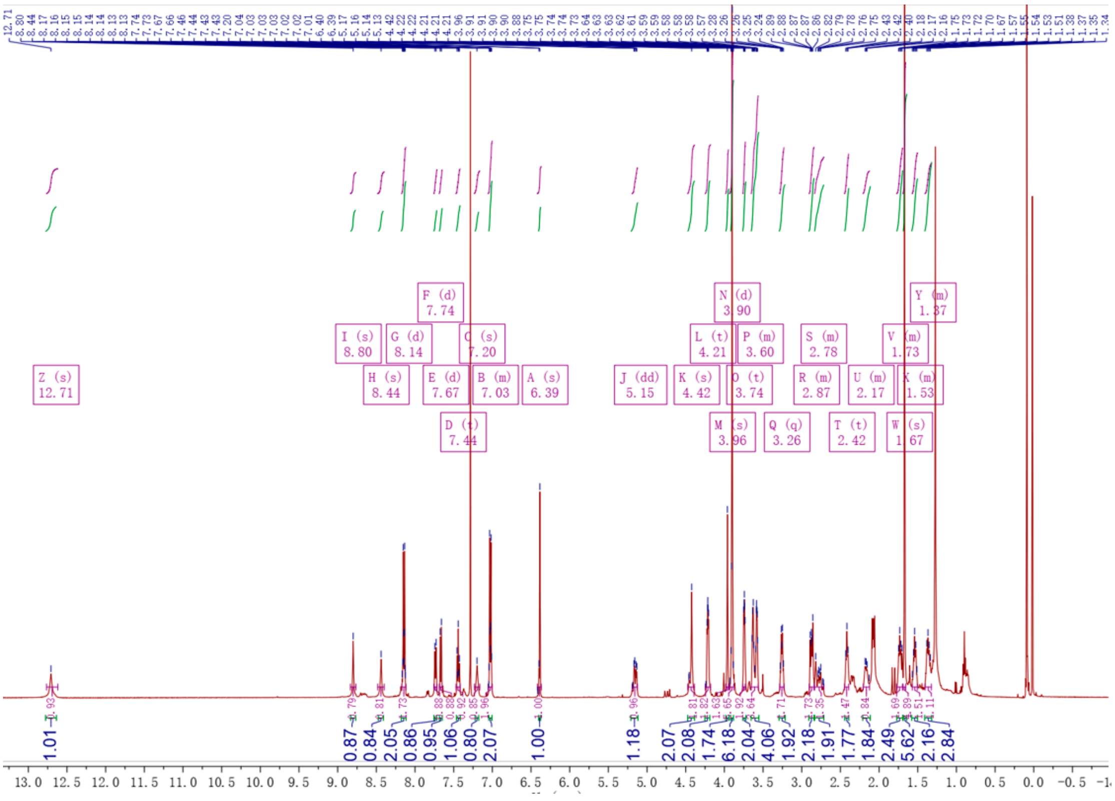

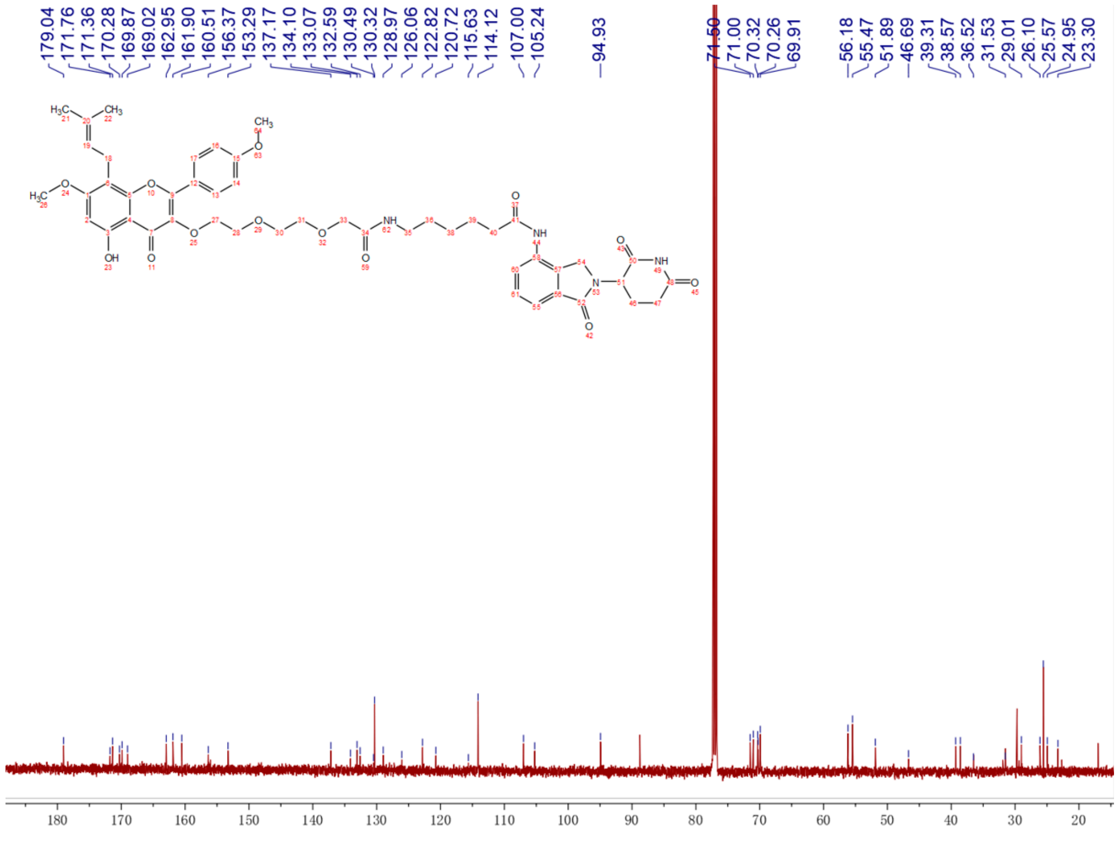

Supplementary Figure 21: 1H and 13C spectra of compounds 19

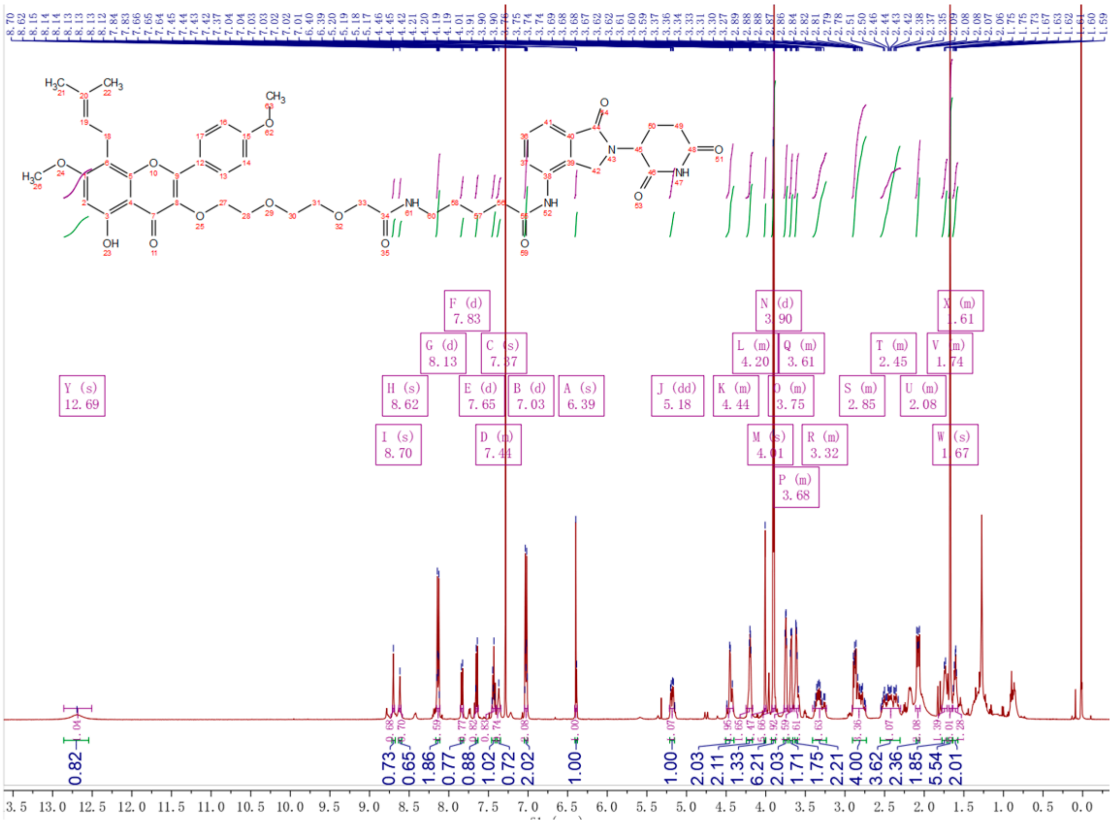

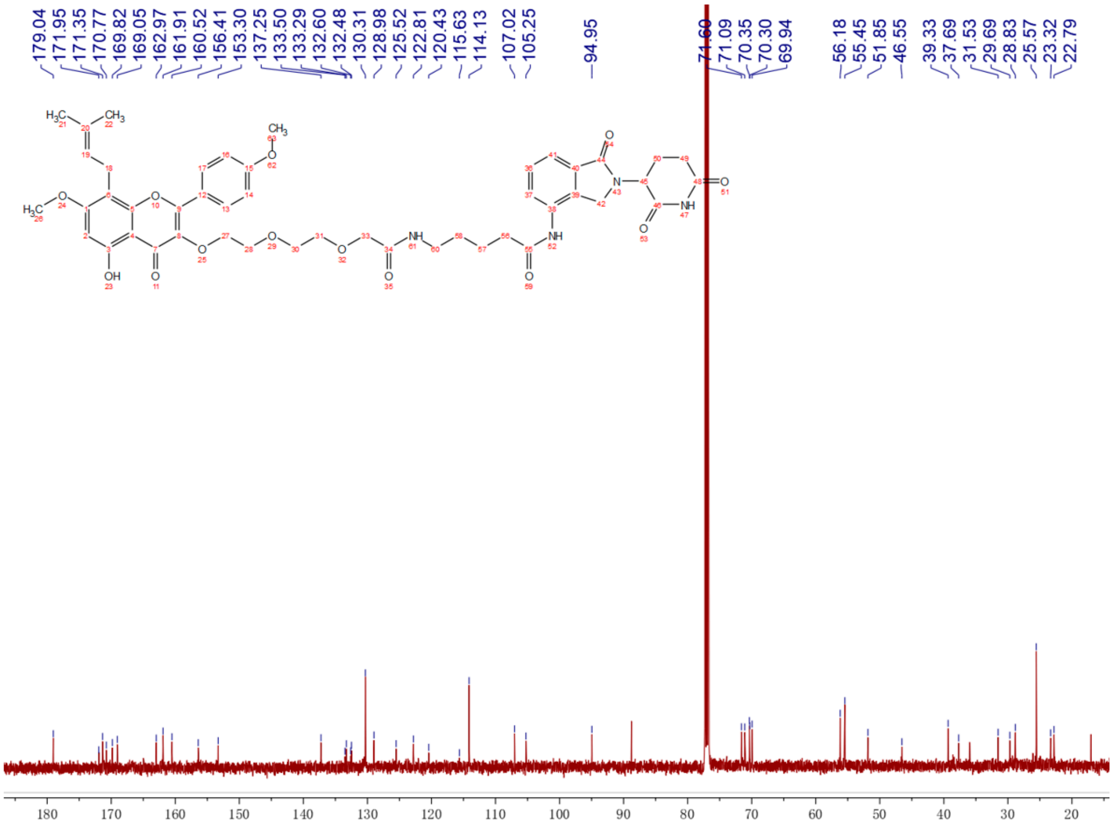

Supplementary Figure 22: 1H and 13C spectra of compounds 20

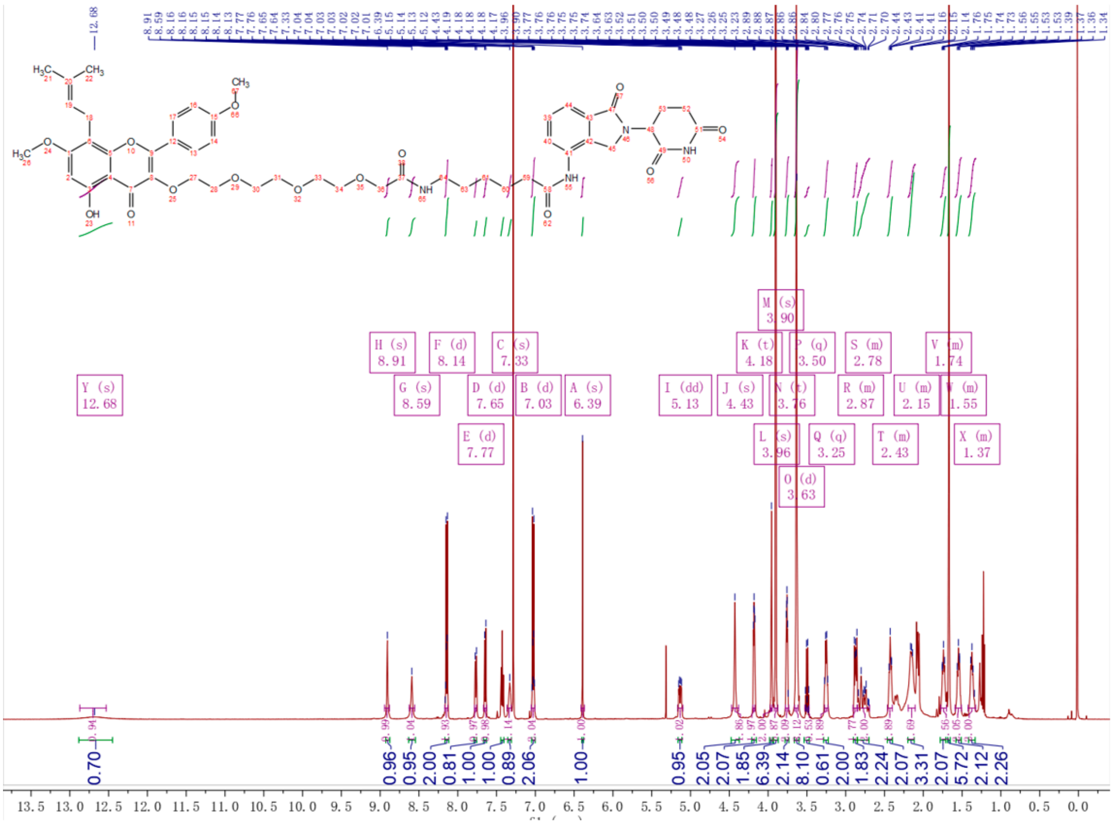

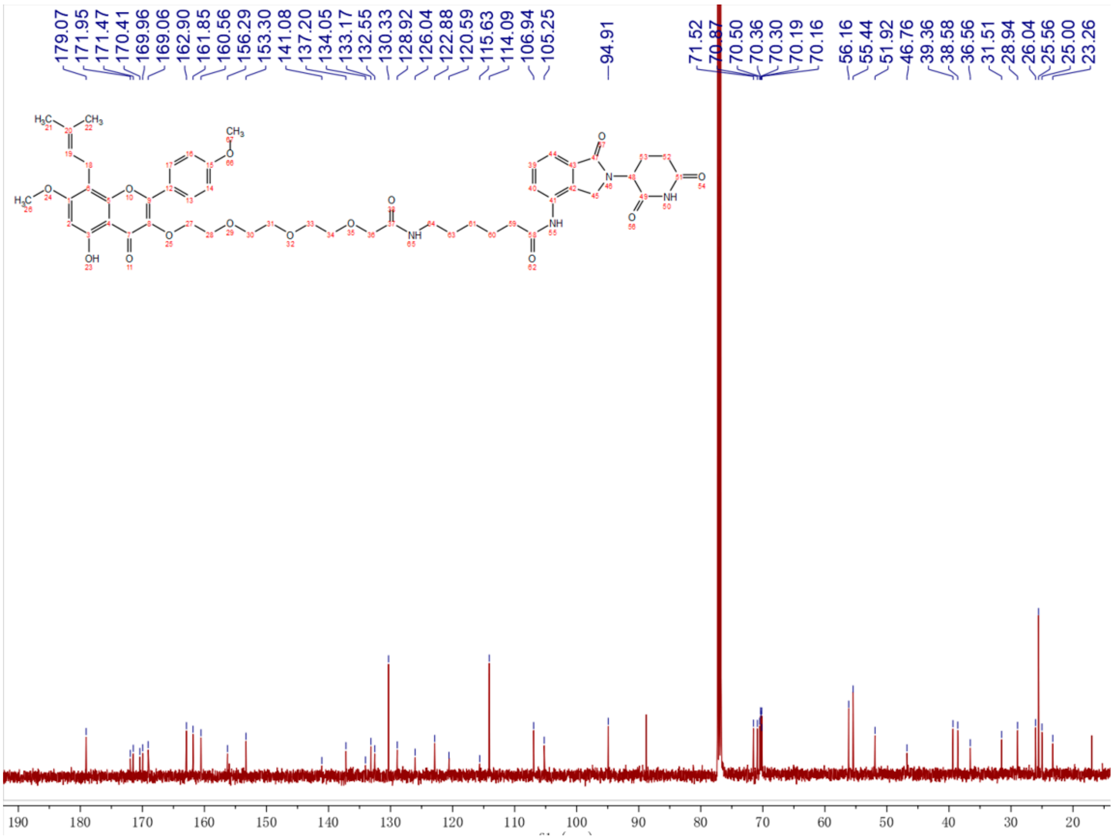

Supplementary Figure 23: 1H and 13C spectra of compounds 21

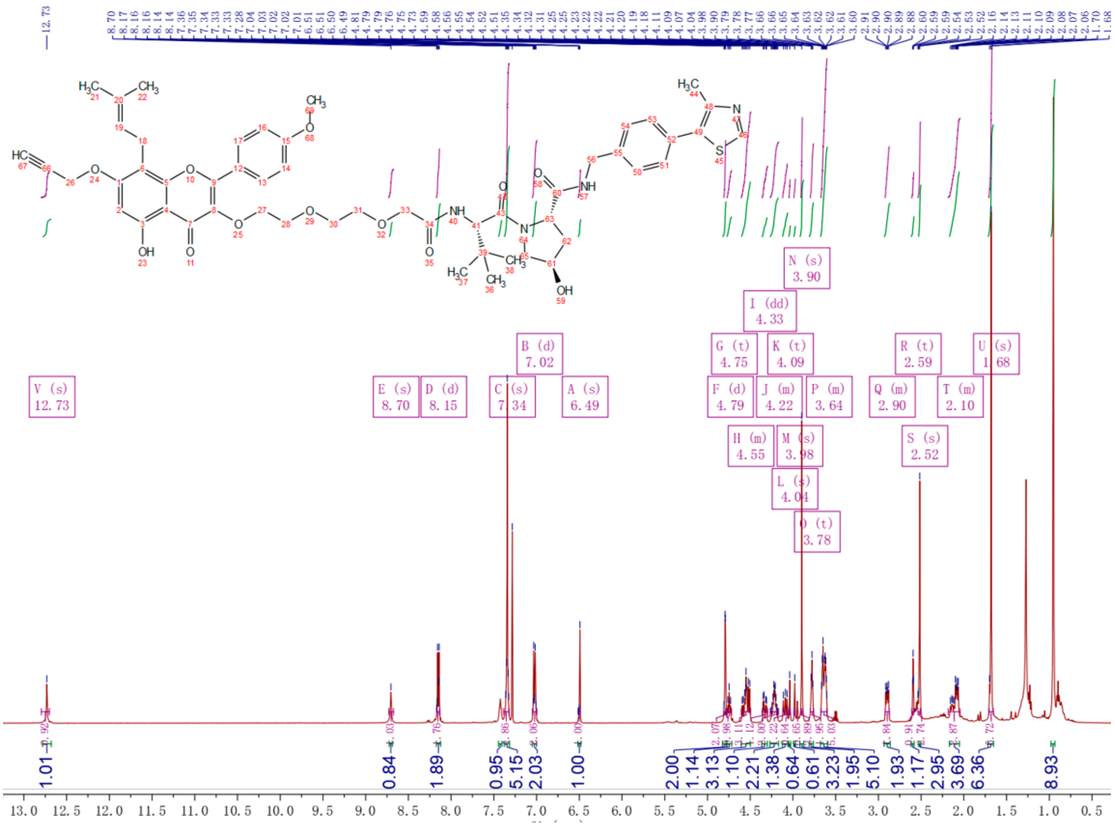

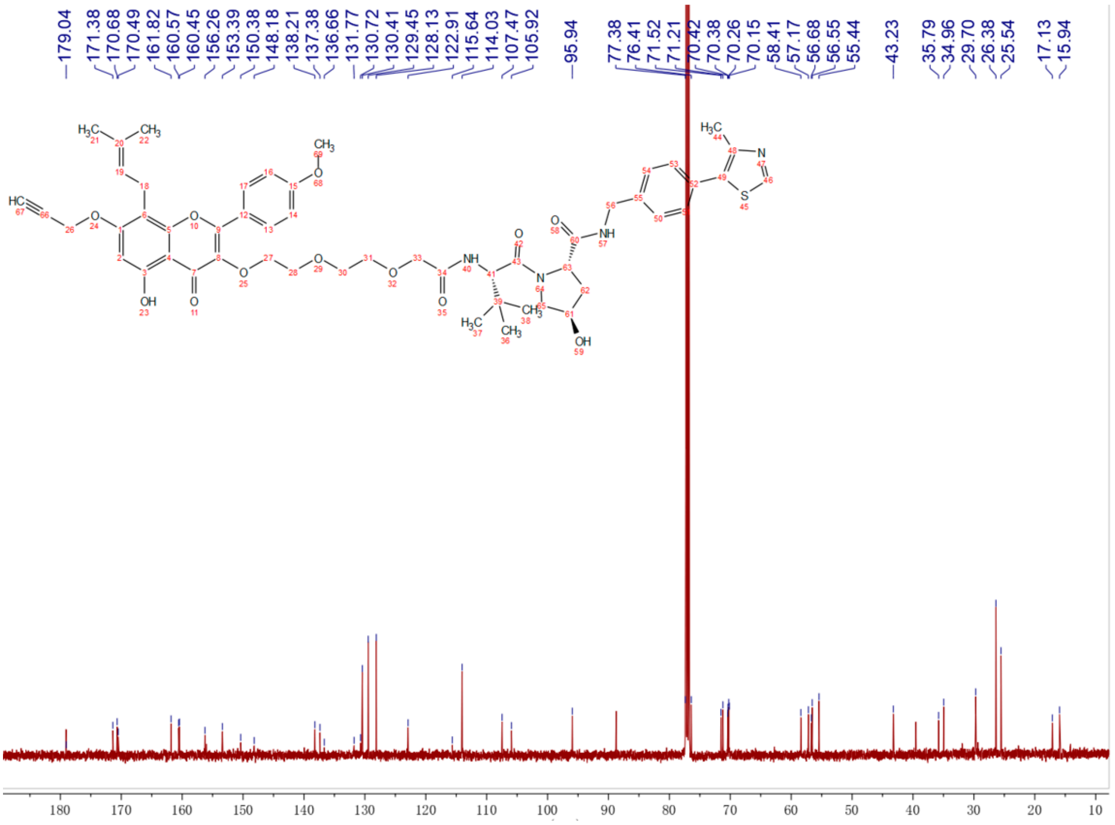

Supplementary Figure 24: 1H and 13C spectra of compounds 22

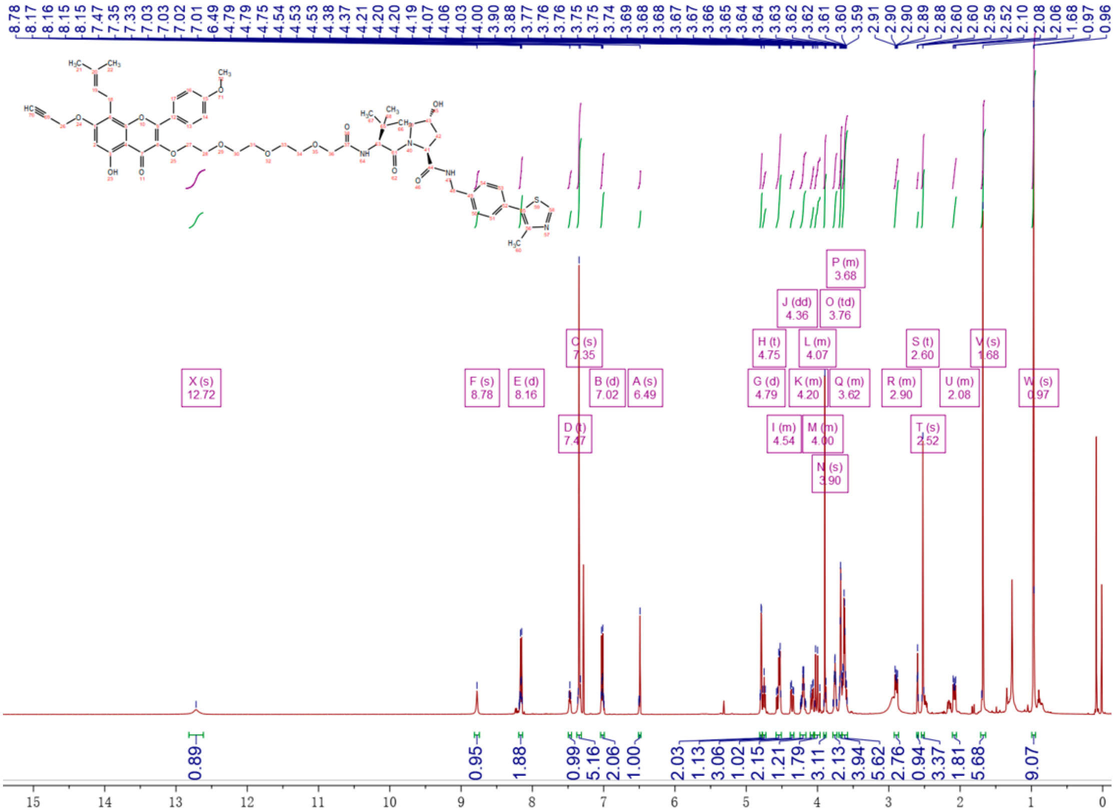

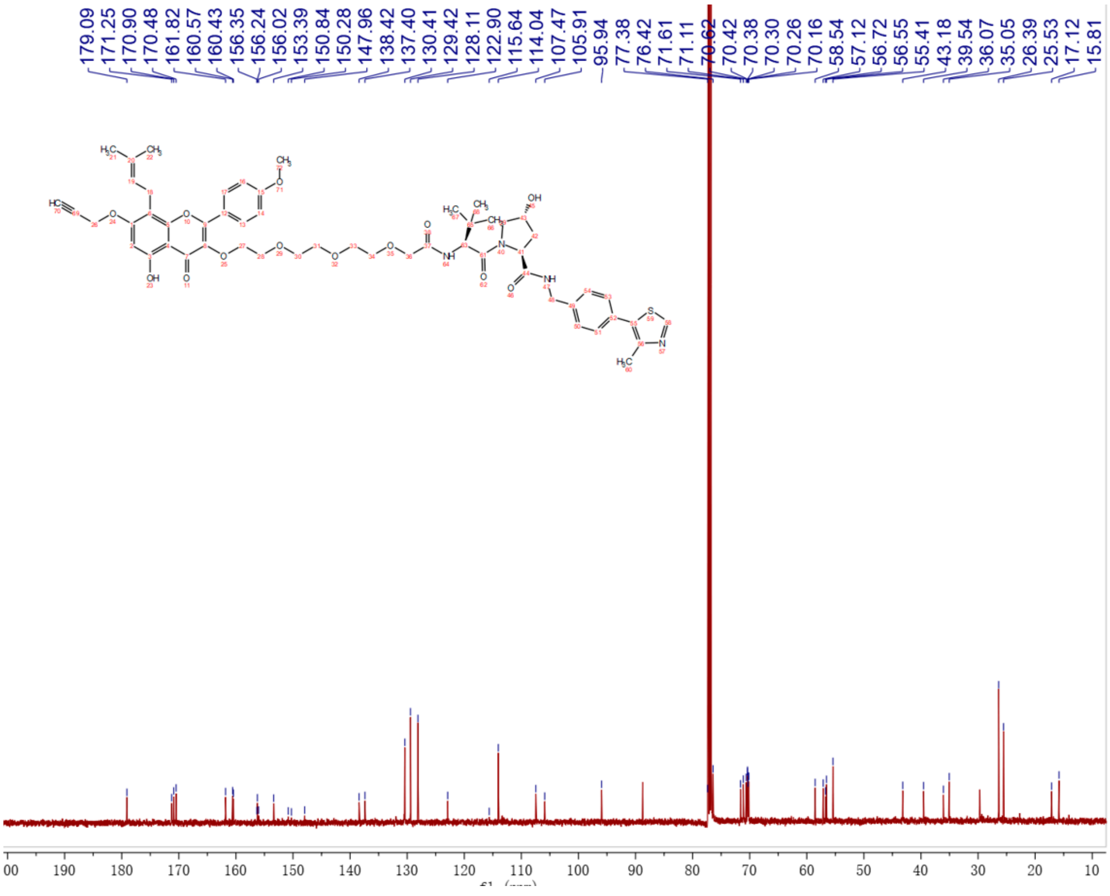

Supplementary Figure 25: 1H and 13C spectra of compounds 23

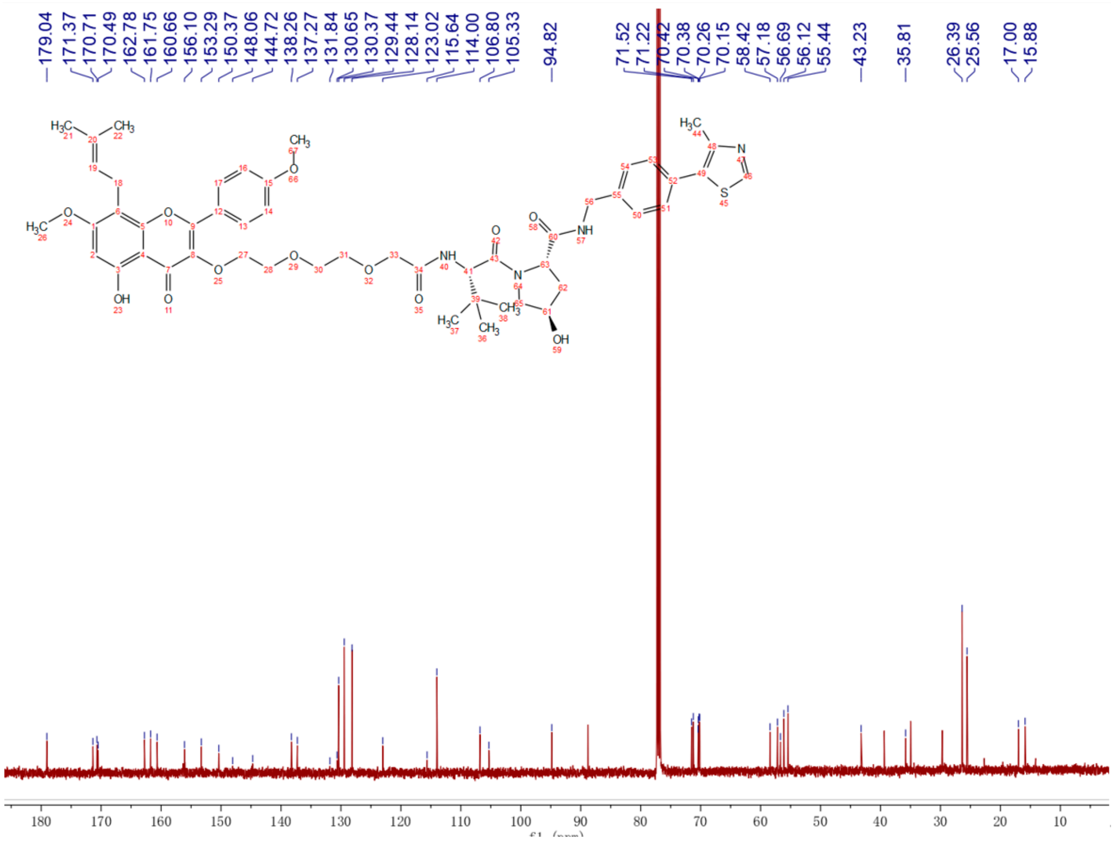

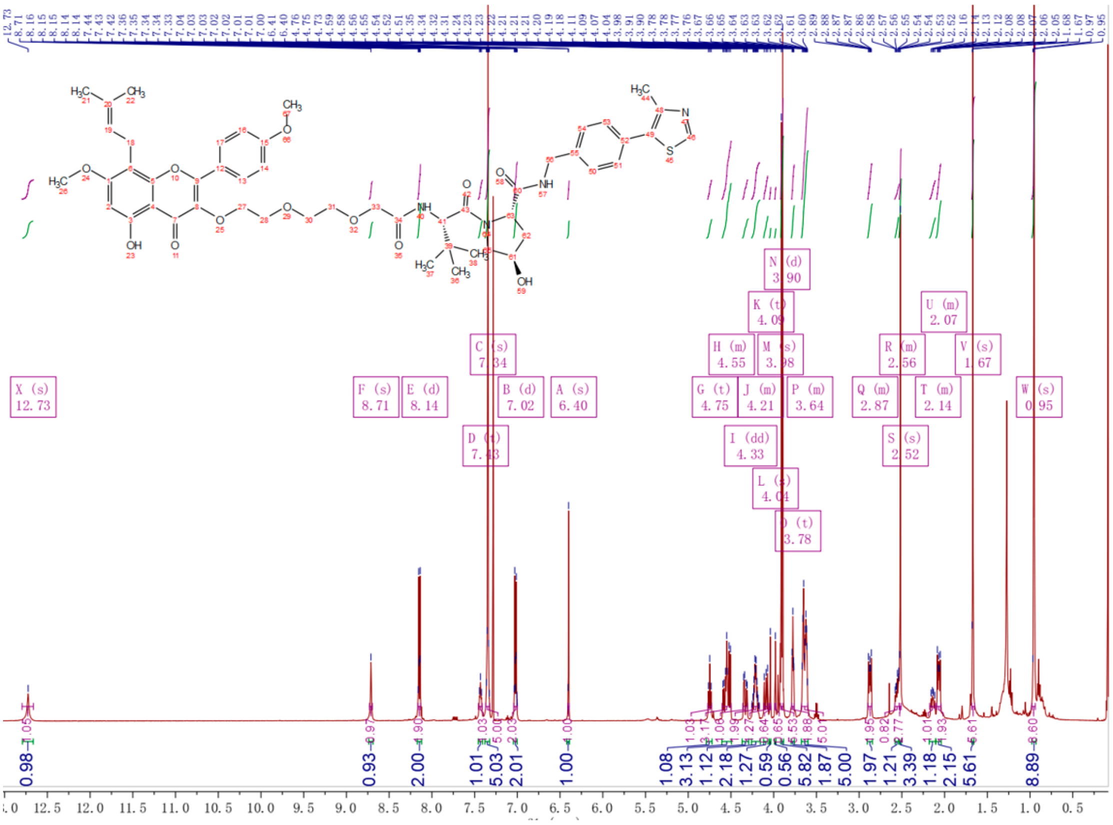

Supplementary Figure 26: 1H and 13C spectra of compounds 24

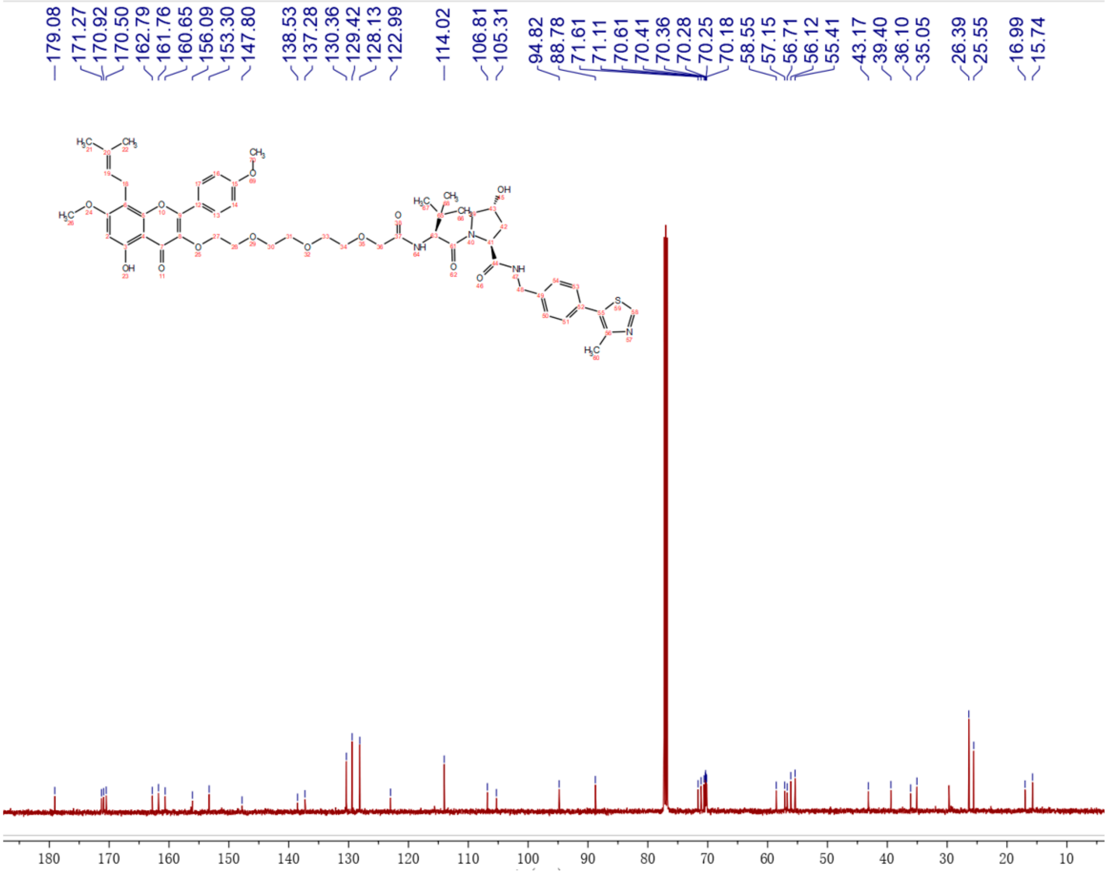

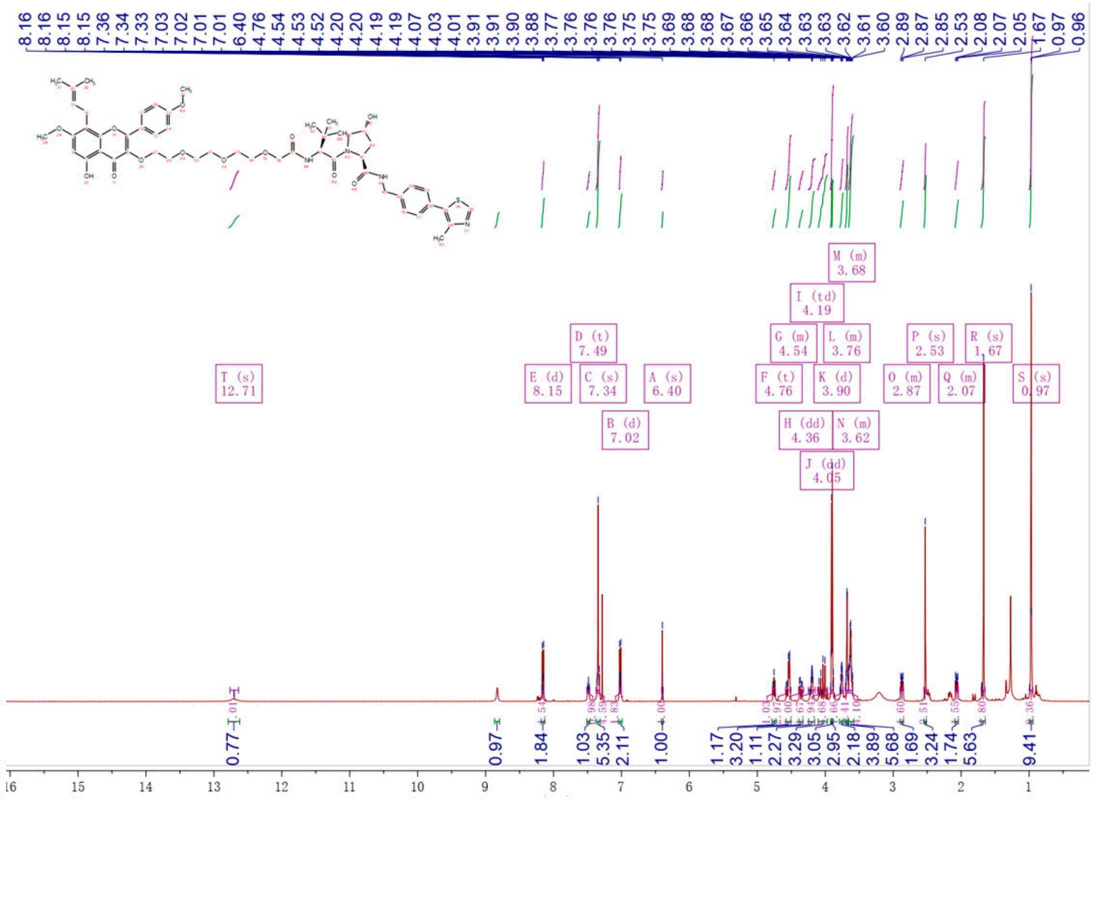

Biological experiment

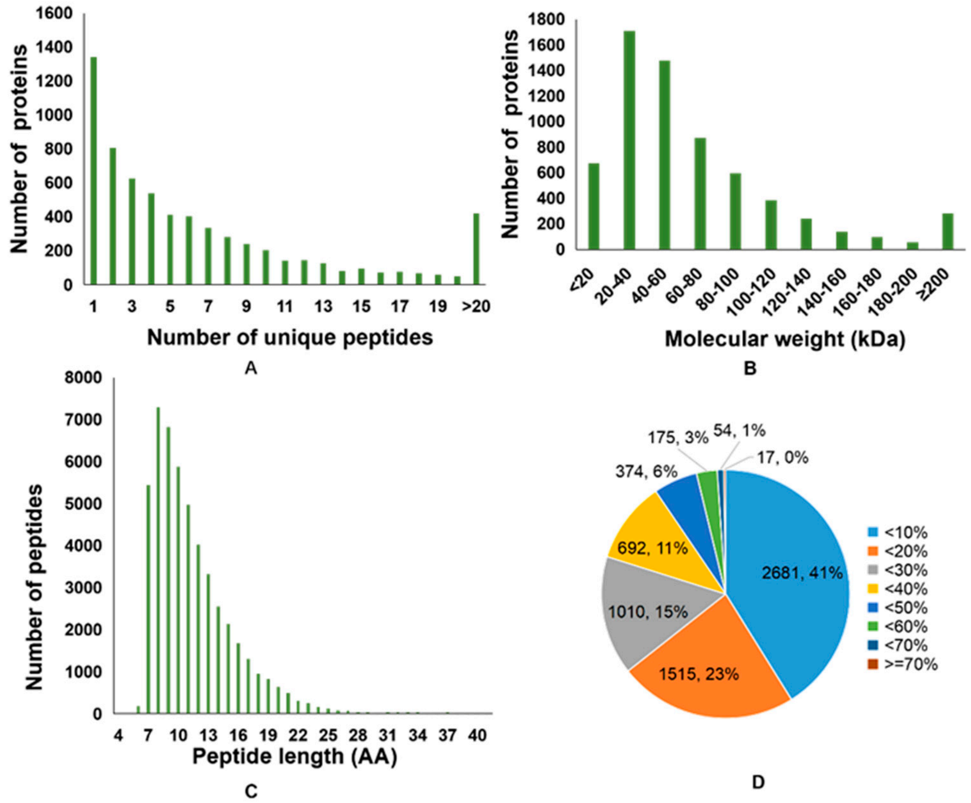

Supplementary Figure 27. (A) Distribution of protein peptide identification numbers, (B) Distribution of protein molecular weights, (C) Distribution of peptide lengths, and (D) Distribution of peptide coverage of protein identifications

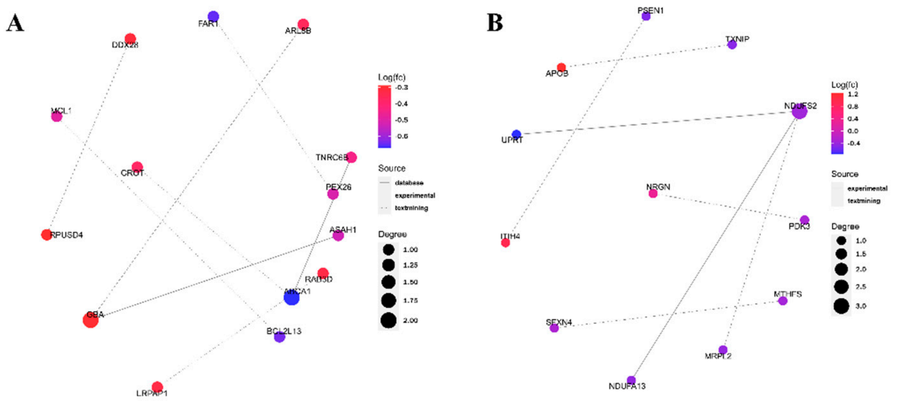

Supplementary Figure 28. (A) The Protein-Protein Interaction Network Analysis after 48 h treatment of a or DMSO control, (B) The Protein-Protein Interaction Network Analysis after 48 h treatment of LJ-41 or DMSO control

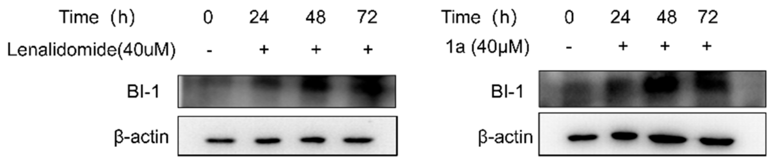

Supplementary Figure 29. (A) single 1a was administrated and showed no degradation effects on BI-1 within 72 h in CA-46 cell. (B) single Lenalidomide was administrated and showed no degradation effects on BI-1 within 72 h in CA-46 cell.
